# Small Molecule Drug Conjugate Hybrids of Naphthalene Sulfonamide and Phospholipid Conjugates Are Microtubule-Disrupting Antitumor Agents

**DOI:** 10.3390/pharmaceutics18091064

**Published:** 2026-08-26

**Authors:** Noelia Fernández-Ceballos, Laura Gallego-Yerga, Rafael Peláez

**Affiliations:** 1 Laboratorio de Química Orgánica y Farmacéutica, Departamento de Ciencias Farmacéuticas, Universidad de Salamanca, Campus Miguel de Unamuno, E-37007 Salamanca, Spain; nferceb@usal.es (N.F.-C.); gallego@usal.es (L.G.-Y.); 2 Instituto de Investigación Biomédica de Salamanca (IBSAL), Facultad de Farmacia, Universidad de Salamanca, Campus Miguel de Unamuno, E-37007 Salamanca, Spain; 3 Centro de Investigación de Enfermedades Tropicales de la Universidad de Salamanca (CIETUS), Facultad de Farmacia, Universidad de Salamanca, Campus Miguel de Unamuno, E-37007 Salamanca, Spain

**Keywords:** tubulin, alkylphospholipids, antitumor, hybrids, small-molecule drug conjugates, mechanism of action, mitosis, MDR, docking

## Abstract

**Background**: Antimitotic agents are very successful antitumor therapies, but lack tumor selectivity, causing toxicity. Antitumor alkylphospholipids (APLs) selectively accumulate in tumor cells but display low potencies. **Hypothesis:** Incorporating APL moieties onto antimitotic *N*-trimethoxyphenyl naphthalene sulfonamides (TMNS) might afford SMDCs with the potency of antimitotics and the tumor selectivity of APLs. **Methods:** 24 new TMNSs with spacers of different lengths (4 to 9 atoms) and nature (alkanes or ethers) on the sulfonamide nitrogen and capped with phosphorus-containing groups such as diethylphosphonates, phosphonic acids, and hydrogenophosphonate esters of aminoalcohols (diethylaminopropanol, choline, or dimethylaminoethanol) were designed and synthesized. Their antiproliferative effects against several cancer cell lines and their cotreatment with verapamil to assess whether they are substrates of MDR pumps were evaluated. The mechanism of action was studied: cell cycle effects, apoptosis induction, and immunofluorescence microscopy. Computational studies considered binding to tubulin and pharmacokinetics. **Results:** Diethyl phosphonates and phosphonic acids are antiproliferative in the micromolar to submicromolar range. P-gp inhibitor verapamil renders inactive compounds active, suggesting that efflux, not binding, removes activity. Mechanistic studies agree with an antimitotic action. Proposed binding to tubulin is similar to TMNS, with the phospholipid-like substituent projecting towards the interdimer space. **Conclusions:** Hybridization of TMNSs with potentially tumor–targeting APLs yields microtubule-disrupting antitumor compounds. However, the modifications assayed turn the compounds into substrates of MDR. These compounds are a proof of concept of the strategy that might succeed if future modifications avoid MDR and might target the compounds towards cancer cells.

## 1. Introduction

Despite recent advances in targeted therapies, traditional cytotoxic chemotherapy, mainly represented by alkylating agents, antimetabolites, antimitotics, and topoisomerase inhibitors, continues to play an essential role in the clinical battle against cancer due to its unparalleled efficacy in killing cancer cells [1,2,3]. Their low selectivity, however, severely limits their applicability and results in suboptimal cure rates due to the difficulty in achieving a balance between therapeutic efficacy and systemic toxicity and the ensuing development of resistance. In recent years, selective targeting of otherwise unselective highly cytotoxic agents to cancer cells by means of conjugation to selective specific vectors (drug vectoring) has become a promising strategy in the development of new “magic bullets”, e.g., antibody-drug conjugates (ADCs), peptide-drug conjugates (PDCs), and small molecule-drug conjugates (SMDCs) [4,5]. However, their conjugate nature consists of a targeting moiety (either an antibody, a peptide, or a small molecule) that binds specifically to a cancer-specific receptor, a cytotoxic payload, and a linker to connect them, resulting in large molecules, particularly in the case of ADCs and PDCs, with unfavorable pharmacokinetic, immunogenic, and cell-penetrating properties and poor solid tumor penetration. Furthermore, tumor heterogeneity often results in different degrees of target expression, thus compromising delivery.

Many tumor-cell types have altered lipid metabolism and plasmatic membranes with abnormal lipid compositions, often accompanied by changes in the surface proteins that contribute to their phenotypic plasticity [6]. Lipid drug conjugates are therefore being explored for enhanced drug delivery, mostly through incorporation in nanoparticles, but less frequently for direct targeting [7]. Phospholipid ethers (PLEs) such as phosphocholine lipid ethers are more abundant in human cancer cells than in normal cells, and endogenous lipid ethers promote tumor aggressiveness [8]. Analogously, synthetic PLEs such as edelfosine and aryl PLEs and alkyl phosphocholine (APC) [9] accumulate in cancer cells and could be used as anticancer agents or as tumor-targeting vehicles [10,11,12,13,14]. Many efforts are being devoted to establishing the structural requirements for tumor retention of PLE analogues [9,15,16]. The incorporation of iodine isotopes in their structures has yielded targeted radiotherapy agents such as CLR131 or [^131^I]Iopofosine (carrying ^131^I) and diagnostic agents such as CLR124 (carrying ^124^I) [16] and glycophospholipids such as ohmline, acting as a dual agent [13]. These studies have shown that significant structural modifications to PLEs and APLs are allowed, including the incorporation of aromatic moieties as in the fluorescent analogues LAURDAN or Di-4-ANEPPDHQ, used as fluorescent membrane probes of fluidity and for the detection of lipid rafts [17,18,19].

**Figure 1:**
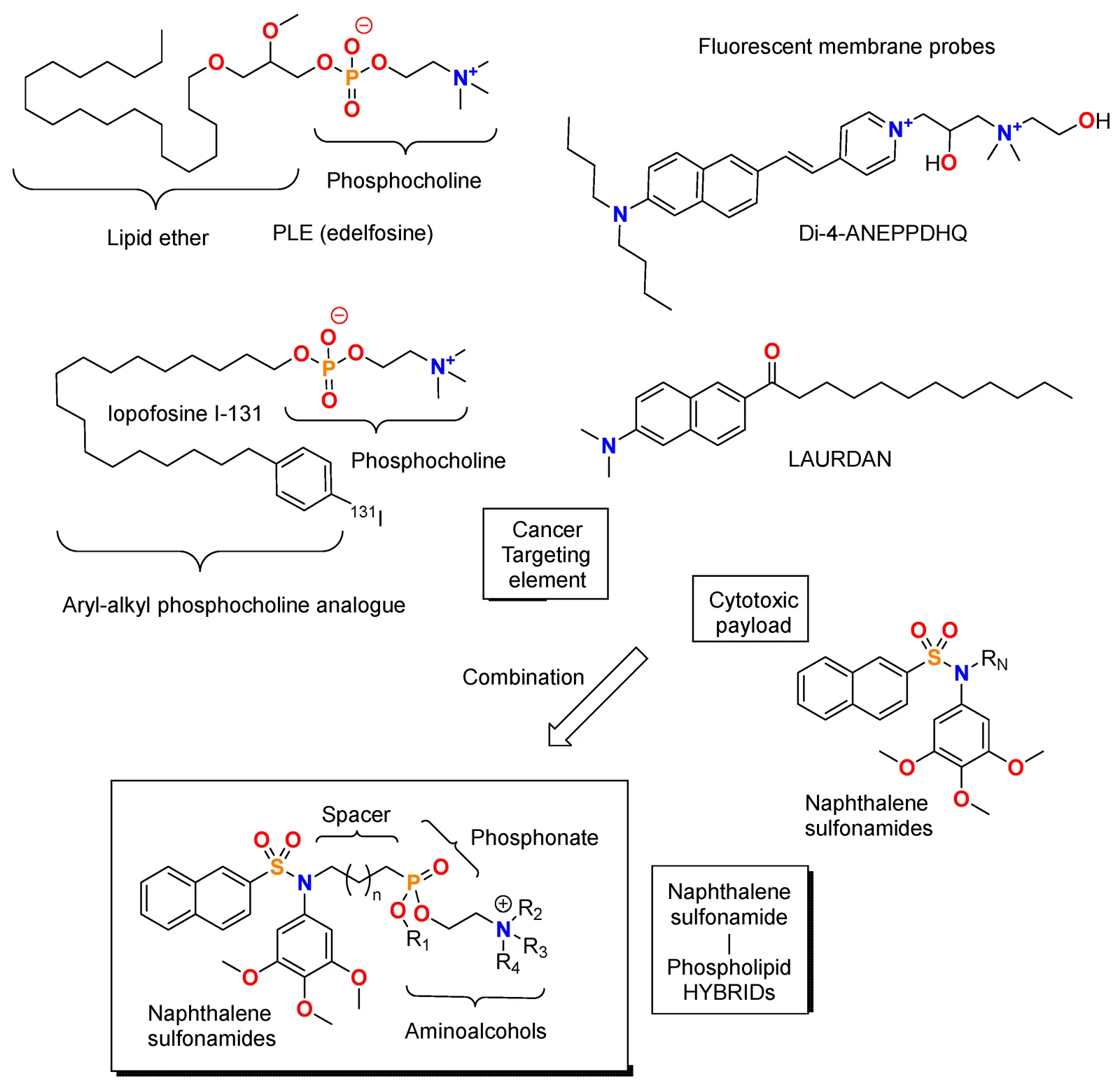
Design of the naphthalene sulfonamide—phospholipid hybrids. The structural elements mentioned in the main text are indicated.

Tubulin drugs are one of the most successful classes of antimitotic compounds, with many representatives of clinically approved drugs, including paclitaxel, docetaxel, vincristine, eribulin, colchicine, and tirbanubulin [1,2,3,20]. They bind to the many drug-binding sites found in tubulin [20,21], being classified as either polymerizing or depolymerizing agents depending on their effects on microtubule mass at high concentrations. However, they show poor selectivity against tumor cells and therefore their application is limited by their toxicity. Ligands of the colchicine binding site are structurally simpler than ligands binding to most of the other sites, and are therefore more suitable for conjugation purposes [22,23]. In a related rationale, lipid conjugates of allocolchicinoids have been explored by Fedorov’s group mostly in the context of controlled drug release by enzyme-responsive liposomes [24]. We have recently described diaryl sulfonamides with potent antitumor activity that might be suitably modified for conjugation [25,26,27,28,29]. Considering the structures of fluorescent membrane probes with naphthalene moieties, naphthalene sulfonamides stand out as a good possibility for conjugation with lipids (Figure 1). Furthermore, they are not substrates of multidrug resistance pumps (MDR), one of the most frequent resistance mechanisms of tumor cells. The colchicine domain is often subdivided into three sub-pockets known as Zones I, II, and III [21,30,31]. Naphthalene sulfonamides are proposed to occupy Zones I and II, with the sulfonamide sitting between them and pointing toward the interfacial space, which might accommodate the phospholipid.

In this work, we have explored the effect of introducing alkyl phospholipid-like substituents on the sulfonamide nitrogen of naphthalene sulfonamides (Figure 1). Small hydrophobic substitutions on the sulfonamides, such as methyl, cyanomethyl, and alkoxycarbonylmethyls, result in highly potent cytotoxic compounds [32], but here we have extended these substitutions to alkylphosphonates, alkylphosphonic acids, and different esters with aminoalcohols that have been shown to produce more potent antiproliferative results in PLEs to explore if they can presumedly occupy the tubulin interdimer space, inhibit tubulin polymerization, and arrest cell proliferation in human tumor cell lines. We assayed the antiproliferative effects of the PLE—naphthalenesulfonamide hybrids against several tumorigenic human cancer cell lines of diverse origin, their effects on cell distribution across cell cycle phases, and their ability to induce cell death. We also assessed whether they are sensitive to MDR pump efflux, a problem in APLs. The effects of the most active compounds on the microtubules of cells were studied by immunostaining experiments. We ran docking simulations to propose the binding modes and interactions with tubulin.

## 2. Results and Discussion

- **Chemical synthesis**

**Scheme 1:**
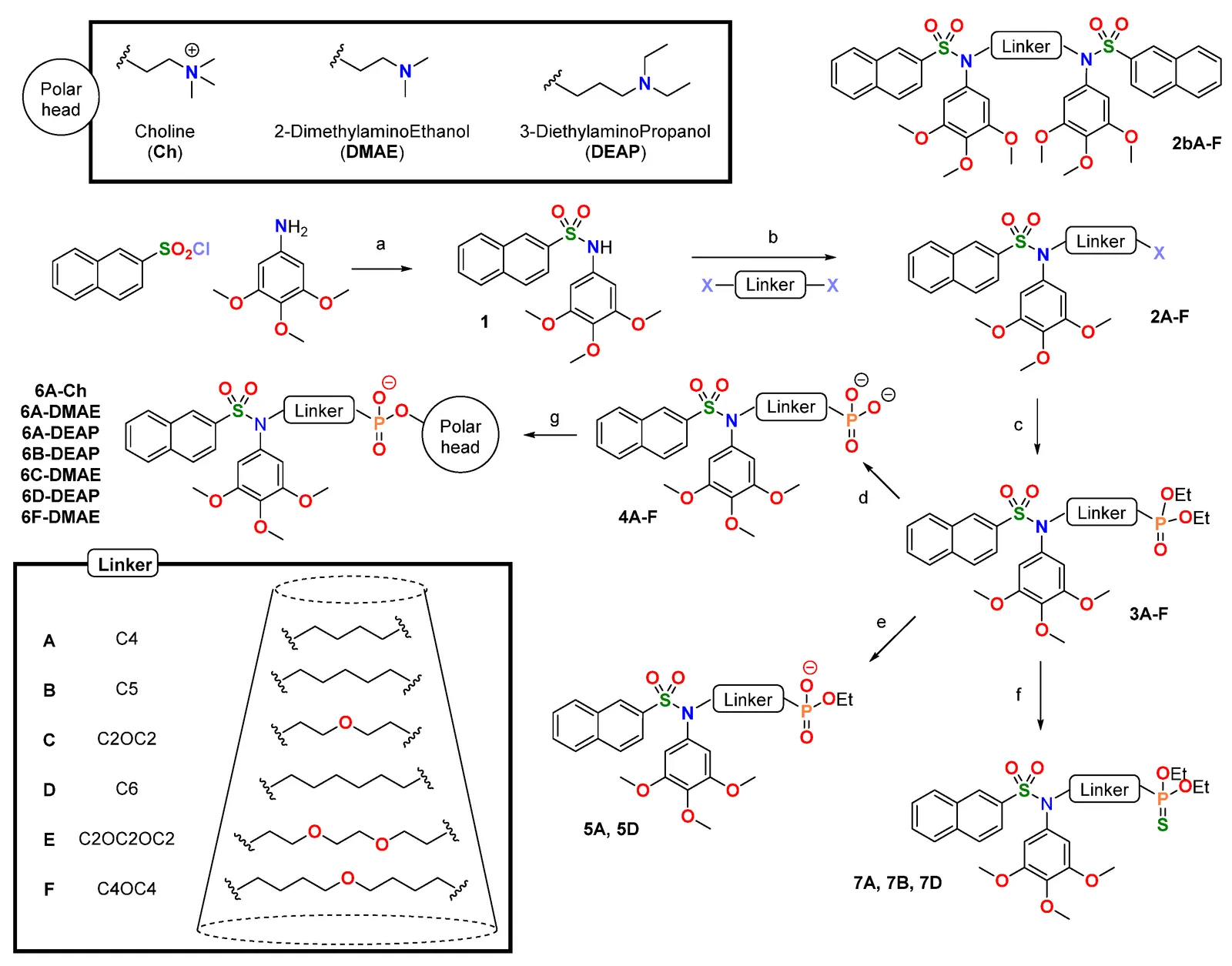
Synthesis of sulfonamides. Reagents and conditions: (**a**) Pyridine, CH_2_Cl_2_, rt, 24 h, 76%; (**b**) alkylating agent, KI, Cs_2_CO_3_, CH_3_CN, if X = Br, rt, 72 h, 33–50% for monosubstituted (**2A, B, D**) and 5–23% for disubstituted (**2bA, B, D**) compounds; if X = Cl, reflux, 24 h, 57–67% for monosubstituted (**2C, E, F**) and 1–13% for disubstituted compounds (**2bC, E, F**); (**c**) Triethylphosphite, 150 °C, 24 h, 38–83%; (**d**) DMF, NaBr, TMS-Cl, 75 °C, 18 h, 24–74%; (**e**) NaI, acetone, 100 °C, 18 h, 99% or N_2_H_4_ H_2_O, 130 °C, 24 h, 61%; (**f**) Lawesson’s reagent, toluene, 120 °C, 24 h, 39–71%; (**g**) Alcohol, DMAP, EDC, CH_2_Cl_2_, reflux, 24 h, 21–46%.

Seven naphthalene sulfonamide—phospholipid hybrids with different spacer lengths and polarity (hydrocarbons or ethers) and amino alcohol phosphonates (2-dimethylaminoethanol, 3-diethylaminopropanol, or choline) were prepared according to synthetic Scheme 1. The synthetic route involved assembly of the sulfonamide (a), followed by nitrogen alkylation (b) and introduction of a terminal phosphonic acid (c,d), which finally yielded the phosphonates by esterification with amino alcohols (g). Partial hydrolysis of the intermediate diethyl phosphonates (e) and conversion to the thiophosphonates (f) on selected derivatives to study their effect on the activity were performed.

Naphthalene sulfonamide **1** was obtained in good yield by reacting trimethoxyaniline with naphthalene-2-sulfonyl chloride as described [32]. Alkylation of the sulfonamide nitrogen with dihalogenated spacers, in situ converted into the iodides by the Finkelstein reaction with KI, was performed under basic catalysis with cesium carbonate to activate the sulfonamide nitrogen to achieve alkylated sulfonamides (**2A**-**E**) as mixtures of the possible terminal halogens (iodine and bromine or chlorine coming from either the KI or the dihalogenated reagent). Despite using a large excess (3–5 moles per mole of sulfonamide) of the dihalogenated reagent, products of the double substitution (**2bA**-**F**) were obtained in proportions exceeding the stoichiometric expectation. The proportion varies with the linker, thus suggesting structure–dependent reactivity. The sulfonamide nitrogen was substituted with alkyls or alkyl ethers of different lengths (A-F: from four to nine atoms) to explore the effect of these variations on the biological activity.

The diethyl phosphonates were prepared in moderate yields by means of a Michaelis–Arbuzov rearrangement, heating the halogen-capped alkyl sulfonamides in neat triethylphosphite. Subsequent hydrolysis with trimethylsilyl bromide (TMSBr), generated in situ with TMSCl and NaBr, gave the phosphonic acids in varied yields, mainly dependent on the isolation difficulties. Partial hydrolysis was carried out with NaI in acetone (**7A**) [33] or hydrazine hydrate (**7D**) [34] with good to excellent yields. Reaction of the diethylphosphonates with Lawesson’s reagent yielded the diethylthiophosphonates in moderate (**7A** and **7B**) to good (**7D**) yields. The naphthalene sulfonamide—APL hybrids were synthesized by esterification of the phosphonic acids with the aminoalcohols using a carbodiimide (EDC) to activate the acid. The isolated yields were low due to the difficulty in purifying the desired phosphonates from the intermediate phosphonyl isoureas, which do not fully react to the desired phosphonates due to steric hindrance and the low nucleophilicity of the alcohols. The purification is further complicated by the formation of salts of 4-DMAP. Therefore, several chromatographic separations and solid-liquid extractions with different solvents are required to achieve the pure products for the biological assays (Supplementary Material: Supplementary Figures S16–S38).

b. **Biology**
  - **Phosphonate—capped naphthalene sulfonamides, except the aminoalcohol esters, are antiproliferative agents.**

The antiproliferative effect of the phosphorus-containing naphthalene sulfonamides was evaluated against the human cancer cell lines HeLa (cervix epithelioid carcinoma), a sensitive cell-line towards anti-tubulin sulfonamides [21,26,28,29,35], and HT-29 (colon adenocarcinoma), that shows much less sensitivity to colchicine-site agents such as combretastatin A-4 [36] but is sensitive to alkylphospholipids such as edelfosine [37], after 72 h of treatment at a concentration of 10 µM using the MTT method. Compounds that inhibited proliferation by more than 40% compared with the untreated control in three independent experiments were assayed at different concentrations, and their IC_50_ values were calculated (Table 1). The diethyl phosphonates reach potencies in the low micromolar to the submicromolar range for both cell lines (HeLa: 1.24–0.26 µM, HT-29: 1.54–0.34 µM), well under the value for miltefosine (7.26 µM and 2.61 µM for HeLa and HT-29, respectively), and in the same order as the antimitotic sulfonamide ABT-751 (0.20 µM and 0.25 µM for HeLa and HT-29, respectively) used as a reference. The phosphonic acids with a butyl or a 5-oxanonyl spacer have IC_50_ values comparable to miltefosine in HeLa cells, whereas the remaining acids do not show antiproliferative potency under the chosen cutoff. Against HT-29, only the first one is active. The thiophosphonates are less potent than their phosphonate pairs but still active in the low micromolar range for HeLa cells, while only the five-carbon spacer analogue is active against HT-29. The potencies against HeLa are slightly but systematically higher than against HT-29, and these IC_50_ increases in this cell line probably push them above the selected cutoff of 10 µM. The partially hydrolysed products are also inactive, even if, for the butyl derivative, both the diethylphosphonate and the phosphonic acid are active. None of the aminoalcohol ethers (also having a partially hydrolysed phosphonate) is active.

Those compounds that showed IC_50_ values below 10 µM against HeLa or HT-29 cells were assayed against the human glioblastoma cancer cell line U87 MG, as glioblastomas are very chemoresistant cell lines that have recently been shown to be specially sensitive to microtubule-targeting agents [38,39] and in particular anti-tubulin sulfonamides, and the MCF7 (human breast adenocarcinoma) that is especially sensitive to some types of sulfonamides [28]. As before, an initial screen of cell proliferation inhibition at 10 μM was followed by the establishment of the IC_50_ values. Antiproliferative assays on HEK-293 (human embryonic kidney), a non-tumorigenic cancer cell line, as a surrogate for normal cells, were performed. ABT-751, a representative of colchicine-site sulfonamide in the clinic, and miltefosine, a PLE, were used as positive controls (Table 1, Supplementary Material: Supplementary Table S1, ST1 and Supplementary Figure S1, SF1).

Overall, all the cell lines show similar sensitivities towards the compounds, with modest differences in their respective IC_50_ values. MCF7 shows sensitivities similar to HeLa for phosphonates and slightly higher IC_50_ values for phosphonic acids and thiophosphonates. U87 behaves similarly, with higher increases in IC_50_ values for phosphonic acids and thiophosphonates. HEK-293 cells show similar sensitivities as HeLa cells, with low selectivity indexes, except for phosphonic acid **4F**, which shows a selectivity index comparable to miltefosine. On the other hand, sulfonamide ABT-751 shows similar potencies against all the cell lines, including HEK-293, and therefore has poor selectivity indexes.

These results suggest that the naphthalene sulfonamides tolerate substitutions on the sulfonamide nitrogen, incorporating spacers capped with phosphorus groups of different polarity. Intriguingly, the partial hydrolysis of diethyl phosphonates abrogates potency, whereas complete hydrolysis to the phosphonates does not. The zwitterionic species **6** do not show antiproliferative potency below 10 μM against any of the cell lines. Amongst the active series, the highest potency is observed for the 5-carbon spacer, with the four- and six-carbon ones following, in what seems an odd-even effect. Introducing an oxygen atom in the 5-carbon spacer substantially reduces potency against all cell lines. Longer (8- or 9-atom) oxygenated spacers are well tolerated, with lower IC_50_ values than the four- and six-carbon spacers. The four-carbon spacer is less effective for the phosphonates, suggesting it is not long enough. The potency modulation by the spacers is maintained over all the active capping groups, suggesting that they reflect binding to a common pocket, which somehow accommodates end groups of different polarity, except for the zwitterionic species.

  ii. **MDR pumps are an important determinant of the activity of the modified naphthalene sulfonamides**

The multidrug-resistant (MDR) phenotype is one of the main mechanisms by which cancer cells exert resistance against chemotherapy, both innate and induced [40]. This mechanism is also one of the main ways cancer cells develop resistance in the clinic against microtubule-targeting drugs [41], thus reducing their efficacy. The MDR phenotype is due to membrane pumps that transfer drugs from inside to outside the cells, thus lowering their intracellular concentrations, reducing their binding to their targets, and their anticancer activity [40,42,43]. We have undertaken a pharmacological approach to study the effect of MDR pumps on the activity of the compounds, comparing the IC_50_ values of the treatments with the compounds in cotreatments with verapamil (VP) at concentrations that are not toxic to the cell line (i.e., HT-29) with those obtained in its absence (Table 1). Verapamil is a P-glycoprotein 1 and multidrug resistance protein 1 (MRP1) [44,45,46] inhibitor; therefore, if the compound is an MDR substrate, its IC_50_ is significantly reduced upon cotreatment with VP. We quantified the effect of verapamil cotreatment as the quotient of the IC_50_ value in the absence of verapamil over the IC_50_ value in the presence of verapamil (Relative sensitivity, RS) and used a three-fold decrease as the cutoff, indicating that the compound is a substrate of the efflux pumps. RS = (IC_50_ non-cotreatment/IC_50_ cotreated with verapamil).

**Scheme 2:**
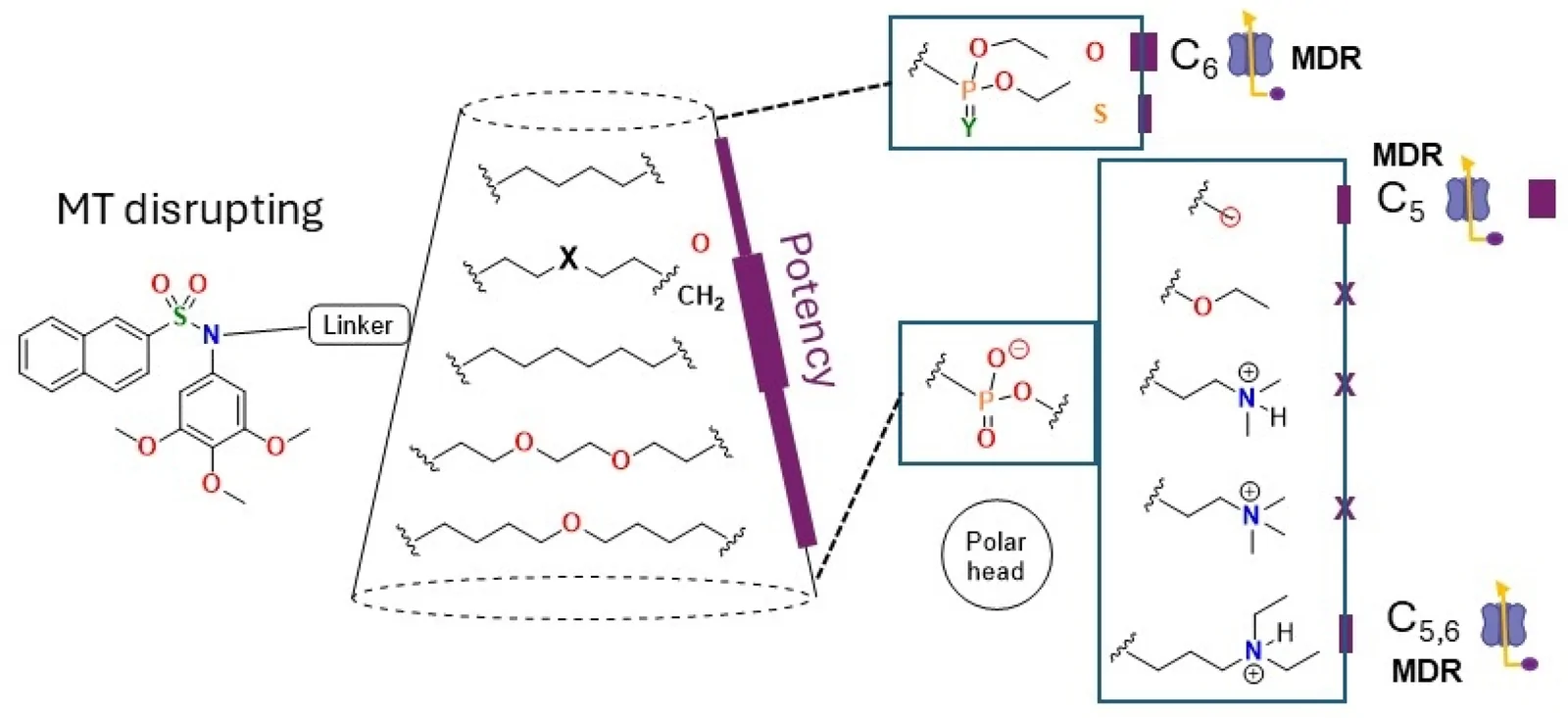
SAR of the anti-proliferative effect of the compounds. The purple bars indicate the relative potency of the compounds (wider bars imply higher potency).

The cotreatment with verapamil results in changes in the potency of many compounds (Scheme 2), turning some inactive compounds (with IC_50_ values > 10 µM) into active ones with IC_50_ values < 10 µM and improving the potency of some already active ones, but not all, thus indicating a structure-dependent effect. This is quite different from what is observed for our previously described naphthalene sulfonamides with *N*-substituents of a different nature, which are not substrates of the MDR efflux pumps. Amongst the diethyl phosphonates, the cotreatment increases the potency, but only significantly for the analogue with a six-carbon spacer **3D**, which then equals the potency of the five-carbon analogue **3B**. This suggests that the apparent odd-even effect suggested by the antiproliferative data alone is indeed a reflection of two different effects: the four-carbon spacer is too short, and the six-carbon spacer results in enhanced efflux, therefore reducing the potency with respect to the five-carbon spacer. The similar potency of the five- and six-carbon spacers suggests that the optimal length is in this range.

The co-treatment with verapamil has a dramatic effect on the potencies of the phosphonic acids, turning all of them, except the one with the oxygenated, nine-atom spacer **4F**, active (with IC_50_ values < 10 µM). In this case, the effect on **4B,** with the five-carbon spacer, stands out, becoming again the most potent of the series with a submicromolar IC_50_ value after a more than one-fold potency improvement. In this case, the improvement of the six-carbon spacer just puts it at the limit, being much less potent than the five-carbon analogue. Again, the oxygen atom in **4C** results in a lower potency, which does not match that of **4B**, thus suggesting the detrimental effect of the oxygen atom is due to the binding to the target. For the thiophosphonates, there is no substantial potency increase upon treatment with VP, thus suggesting that their lower potency compared with the phosphonates is due to the interaction with the target and not to the uptake. The same applies to the ethyl hydrogenophosphonates, which do not improve upon verapamil co-treatment. Finally, a significant recovery is seen for some of the amino alcohol esters with a diethylaminopropanol cap (**6B-DEAP** and **6D-DEAP**), with the five (again most potent) and six-carbon spacers upon verapamil co-treatment. This suggests that the zwitterions can bind to the target, presumably at the interfacial space of the tubulin dimer, but they are rendered inactive by efflux proteins. These results highlight the difficulties in obtaining small-molecule drug conjugates, requiring not only compliance with the pharmacophoric requirements of the target(s) but also avoiding escape from the efflux pumps.

  iii. **Cell cycle**

**Figure 2:**
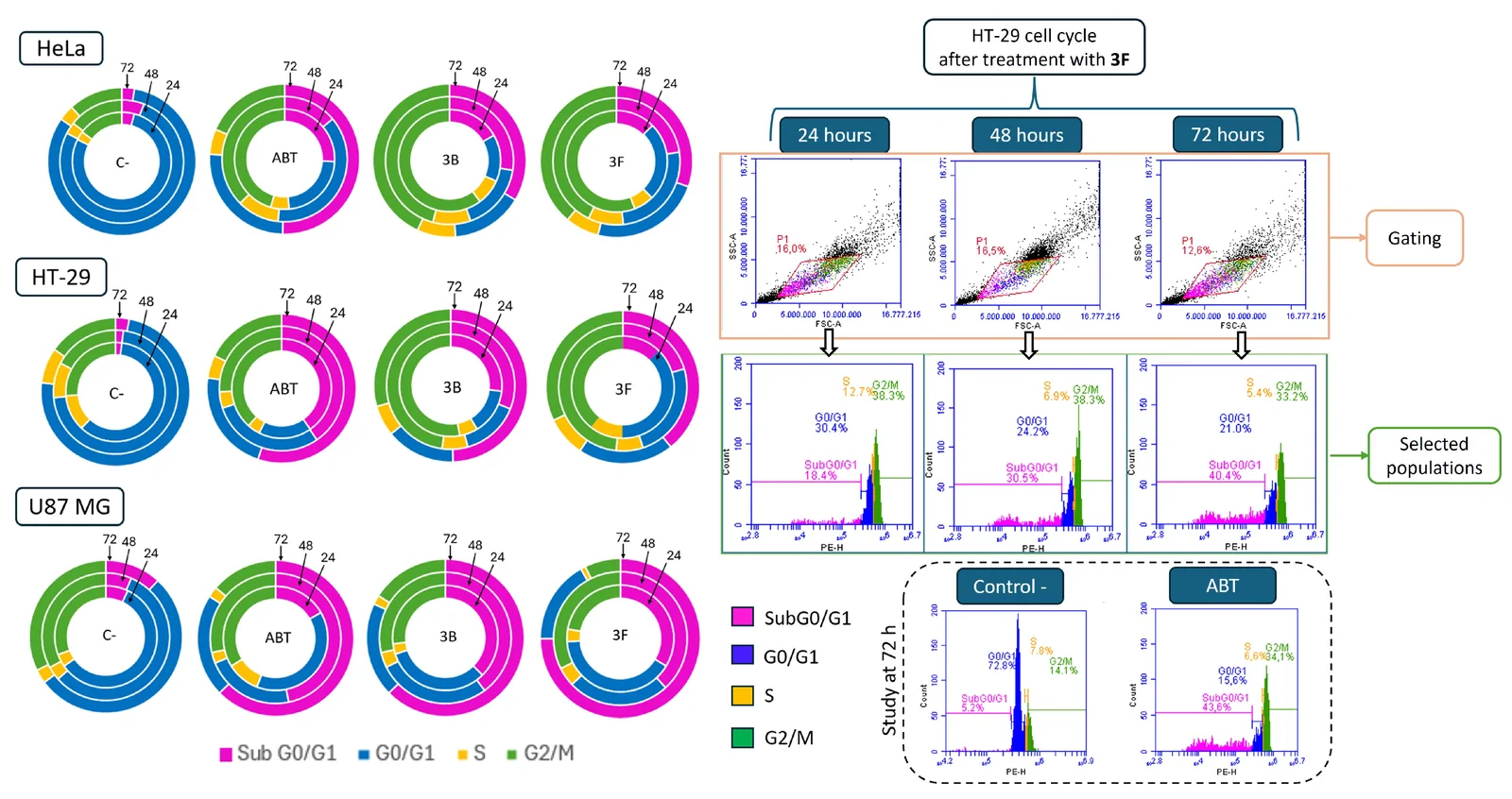
Left: Circle graph representation of the distribution of cells within the cell cycle populations (in order of DNA content, SubG_0_/G_1_ in pink, G_0_/G_1_ in blue, S in orange, and G_2_/M in green) for HeLa (upper lane), HT-29 (center lane), and U-87 MG (lower lane) cells after 24, 48, or 72 h of treatment with 0.5 µM ABT-751, 1 µM **3B**, and 1 µM **3F**. Cells treated with the same final volume of DMSO as the treatments were run in parallel as negative controls (C-). Right: Flow cytometry results and histograms for the treatment of HT-29 cells with compound **3F**.

With the aim of characterizing the mechanism of action of the most active compounds, the phosphonates **3B** and **3F**, we studied their effect on the distribution of cells in the different populations along the cell cycle. We assessed the effect by flow cytometric analysis of the DNA content of HeLa, HT-29, and U-87 MG cells at 24 h, 48 h, and 72 h post-treatment. ABT-751 was a positive control, representative of antimitotic sulfonamides binding to the colchicine site of tubulin (Figure 2, Supplementary Material: Supplementary Tables S2–S7, ST2–ST7).

The assay is based on the fluorescence intensity enhancement that results from the Propidium Iodide’s (PI) intercalation between the DNA bases, that allows quantification of the DNA within each cell and its assignment to populations based on their DNA contents: cells with a 2N DNA content are assigned to the G_0_/G_1_ phases, those with 4N to the G_2_/M phases, cells with DNA amounts in between 2N and 4N are assigned to the S phase, and DNA contents less than 2N to the subG_0_/G_1_ population, usually considered as apoptotic cells [47]. **3B** was tested at 1 µM for the three cell lines, **3F** at 1 µM for HeLa and HT-29, and 1.5 µM for U87-MG, and ABT-751 at 0.5 µM for HeLa and HT-29 and 1 µM for U87-MG. Negative controls were treated with the same volume of DMSO used for the treatments.

The cell populations of HeLa cells treated with solvent (negative controls) remain similar over time: G_0_/G_1_ (77–82%), G_2_/M (12–15%), S (3.0–3,5%), and subG_0_/G_1_ (3–6%). Treatment with ABT-751 arrests HeLa cells at G_2_/M at 24 h, with a reduction at 48 h and close to control at 72 h (45–38–19%, respectively). These changes are accompanied by an initial increase (26%) of the subG_0_/G_1_ population that falls to half (14%) at 48 h, then sharply increases at 72 h, when it accounts for 50% of the cells. **3B** and **3F** show similar profiles, with a larger 24 h G_2_/M arrest than ABT-751 (61 and 56%, respectively), which are slightly reduced at 48 h (46 and 43%, respectively) and 72 h (44 and 39%, respectively). These are accompanied by progressive increases in subG_0_/G_1_ populations (17–28–34% for **3B** and 13–23–31% for **3F**). The positive antimitotic control and the compounds show quite similar trends, with **3B** and **3F** arresting more at G_2_/M and ABT-751 inducing more apoptosis.

Untreated HT-29 cells distribute similarly to HeLa cells, with slightly larger G_2_/M (15–25%) and smaller G_0_/G_1_ populations (62–74%). ABT-751 modestly arrests cells in G_2_/M at 24 h, then brings it back to control levels at 48 h and 72 h (38–26–18%, respectively). Cell counts in subG_0_/G_1_ rise at 24 h and 48 h up to 40%, with a final boost to 55% at 72 h. **3B** initially arrests cells at G_2_/M (52%), sustains it at 48 h (49%), and falls to 29% at 72 h. **3F** shows a slower increase in G_2_/M at 24 h (39%) than **3B**, which continues to increase at 48 h to 48% and ends up with an abrupt fall (32%) at 72 h. This apparent earlier start of **3B** is also observed for the SubG_0_/G_1_ populations, which increase at 24 h (27% for **3B** and 12% for **3F**) to proportionately progress with time (31% at 48 h and 49% at 72 h for **3B** vs. 20% at 48 h and 39% at 72 h for **3F**). The overall trends for the ABT-751 control and the compounds are similar, with subtle kinetic differences.

The untreated U-87 MG cells show higher basal G_2_/M levels (30–34%) than HeLa or HT-29, and lower G_0_/G_1_ (56–57%). ABT-751 treatment in this cell line does not arrest cells at G_2_/M (control-like values of 34% and 29% at 24 h and 48 h, respectively), with a fall to 14% at 72 h, while the subG_0_/G_1_ populations steeply increase over time (17–47–63%). Similar effects are observed for the treatments with **3B** and **3F**, with equal to control G_2_/M levels (no arrest) at 24 h, maintained for **3B** and slightly increased for **3F**, to fall in both of them in a similar way to the ABT-751 treatment. These changes are accompanied by a persistent increase in subG_0_/G_1_ at 24 h and 48 h, larger than the ABT-751 treatment, that finishes with a large increase at 72 h. These differences can well be explained by small kinetic differences in the onset of the cell effects.

The two compounds behave similarly, and differences across the three cell lines are few, mostly arising from kinetic differences in the evolution of the events. These results are consistent with the uniformity of anti-proliferative potencies of the compounds across the cell lines and suggest a common mechanism of action.

  iv. **3B and 3F induce cell death 72 h post-treatment**

**Figure 3:**
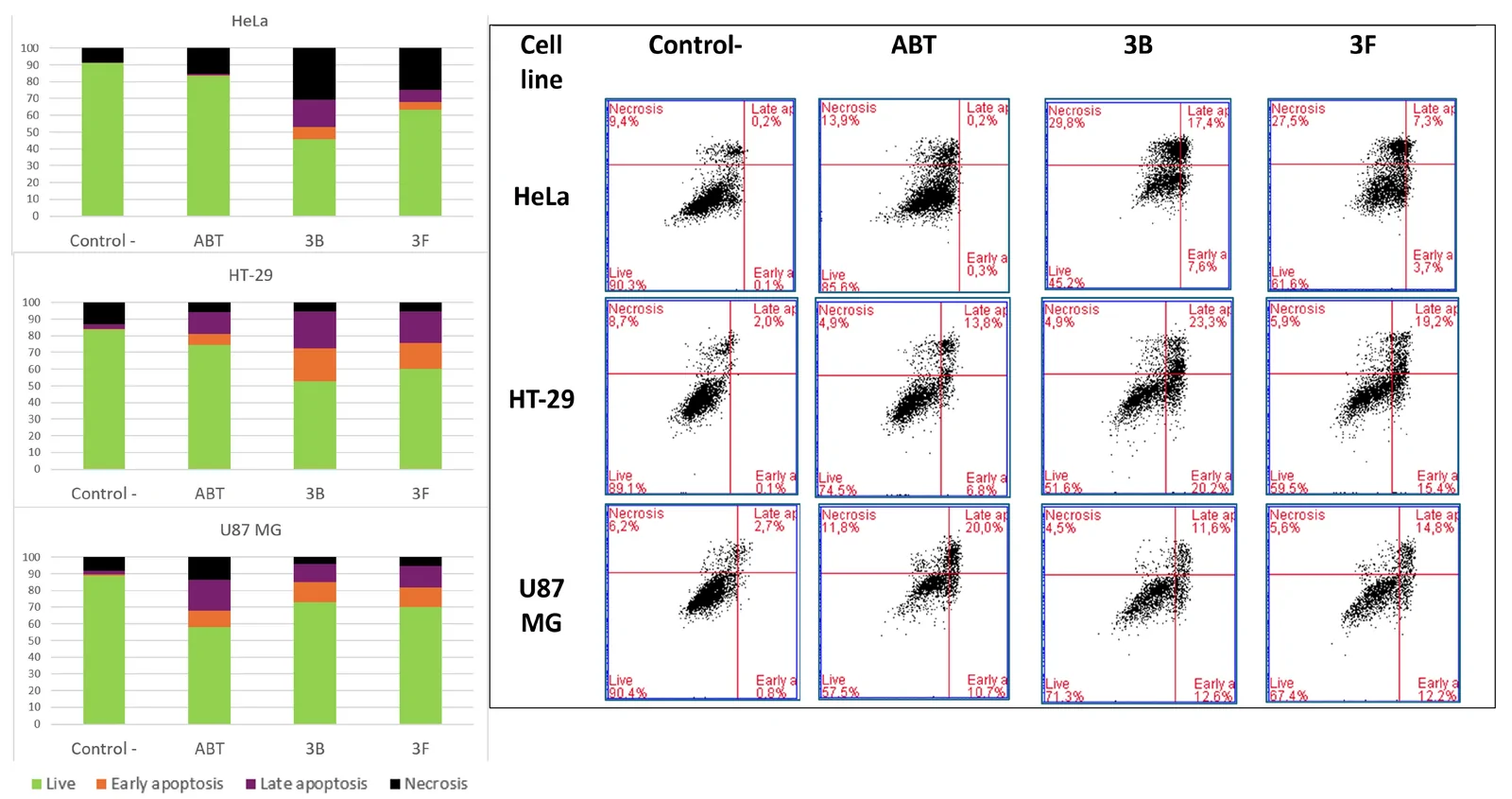
Bar plots of the healthy, early and late apoptotic, and necrotic populations obtained by bidimensional flow cytometry on HeLa, HT-29, and U-87 MG cells 72 h post-treatment with ABT-751 and compounds **3B** and **3F** at the indicated concentrations. Cells treated with DMSO were run in parallel as negative controls.

Aiming to better characterize the mechanism of action we studied the cell death induced in the HeLa, HT-29, and U87-MG cells after 72 h of treatment with **3B** and **3F** at the same concentrations used in the cell cycle experiments (i.e., **3B** was tested at 1 µM for the three cell lines, **3F** at 1 µM for HeLa and HT-29, and 1.5 µM for U87-MG). ABT-751 at 0.5 µM for HeLa and HT-29 and 1 µM for U87-MG were positive controls. Cells were stained with propidium iodide (PI) and fluorescein isothiocyanate-labeled Annexin V (AnV) (Figure 3, Supplementary Material: Supplementary Tables S8 and S9, ST8 and ST9) and subjected to bidimensional flow cytometry. Phosphatidylserine is translocated to the outer leaflet of the cell membrane in apoptotic cells, which are therefore labelled by fluorescent Annexin V (AnV^+^). PI is only taken up by cells in late apoptosis or necrotic stages with damaged cellular membranes (PI^+^). Accordingly, cells were classified by dual-channel flow cytometry into four populations depending on the labeling with the two stains as healthy (PI^−^, AnV^−^), early apoptotic (PI^−^, AnV^+^), late apoptotic (PI^+^, AnV^+^), or necrotic (PI^+^, AnV^−^) [48].

In HeLa cells, treatments with **3B** and **3F** induce cell death substantially above the levels of the untreated control and of those treated with ABT-751. ABT increases apoptosis through the necrotic population, while **3B** increases necrotic and late apoptotic cells to a lesser degree, and even fewer early apoptotic cells. **3F** is similar to **3B** but with a lesser increase in every apoptotic group. **3F** increases cell death compared to ABT-751, and **3B** does so to a larger extent.

For HT-29 cells, increased apoptosis, early and late, is observed for ABT-751, and more in both **3B** and **3F**, which are similar to each other. **3B** and **3F** show a large increase in early and late apoptosis; in both cases, more for **3B**.

In U-87 MG cells, the effects of ABT-751 are larger than those of **3B** and **3F**. The ABT-751 increase is due, in increasing order, to late apoptosis, early apoptosis, and necrosis. For **3B** and **3F**, there is almost no increase in necrotic cells, with smaller increases in late apoptosis than for ABT-751 and larger early apoptosis. Contrary to the other two cell lines, **3F** is here the more potent inducer of the naphthalene-sulfonamides.

As observed for the cell cycle, the compounds and the control behave qualitatively similarly, with the main differences arising between the cell lines. This suggests similar underlying mechanisms for the three, and the differences might arise from kinetic differences.

  v. **3B and 3F disrupt the microtubule network of tumor cell lines**

**Figure 4:**
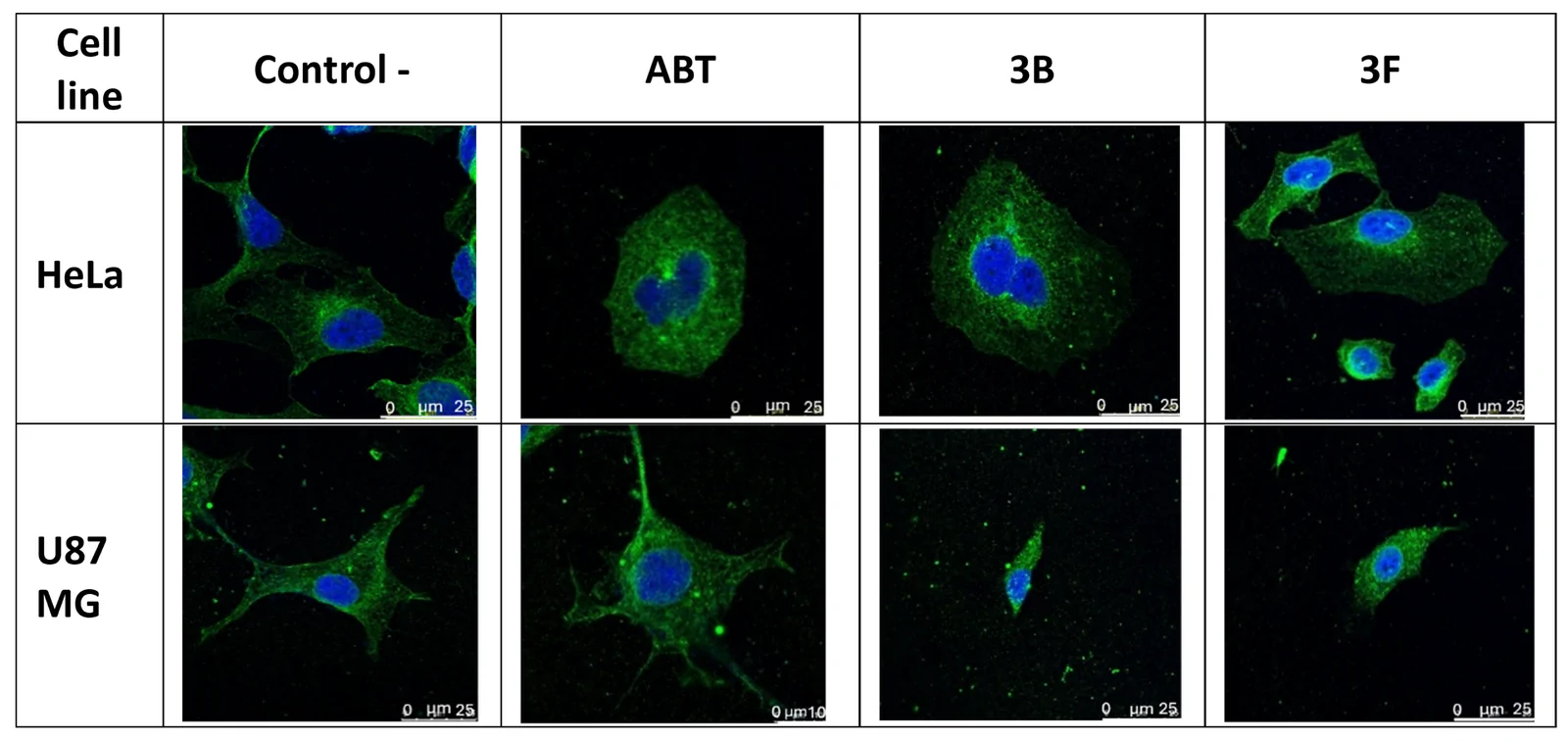
Confocal microscopy images of HeLa (upper row) and U-87 MG (lower row) showing, from left to right, the effects of 0.1% DMSO (Ctrl -), ABT-751, **3B**, and **3F** after 24 h. Nuclei were stained in blue with DAPI and α-Tubulin in green. Scale bar: 25 and 10 µm.

Trying to determine whether the compounds act on the microtubule network, we carried out immunostaining experiments with anti-alpha-tubulin antibodies and DAPI for staining the nuclei (Figure 4). HeLa and U-87 MG cells were treated for 24 h with ABT-751 (at 0.5 µM for HeLa and at 1 µM for U-87 MG), **3B** (at 1 µM), and **3F** (at 1 µM for HeLa and 1.5 µM for U-87 MG) and stained.

The two naphthalene sulfonamides result in apparent changes in cell shape, with cells losing their typical shapes and becoming round, a characteristic phenotype of cytoskeleton disassembly that occurs early during apoptosis. Microtubule network integrity is lost upon treatment in both cell lines, with cells having smaller cytoplasms and many with multilobed nuclei. The effects of ABT-751, a typical microtubule inhibitor, are very similar to those of **3B** and **3F,** thus suggesting a common mechanism of action involving microtubule disorganization inside the cells, reinforcing their proposed mechanism of action as antimitotic agents.

  vi. **3B and 3F impair the migration of HeLa cells**

Metastasis is the main cause of cancer-related deaths, with migration being critical for cancer cell invasion and metastasis. In the in vitro wound-healing assay, cells are grown in monolayers, scratched, and images are taken at regular intervals to measure the gap closure, a surrogate of cell migration. Microtubules play an essential role in cell migration through their involvement in signaling, cell mechanics, intracellular trafficking, and interactions with actin. Tubulin inhibitors are, therefore, potential migration and metastasis inhibitors. The alkylphospholipids, involved in modifying lipid membrane fluidity, are also considered to have anti-migratory effects. Therefore, we set out to study the antimigratory effect of compounds **3B** and **3D** by scratch healing assays on monolayers of HeLa cells.

**Figure 5:**
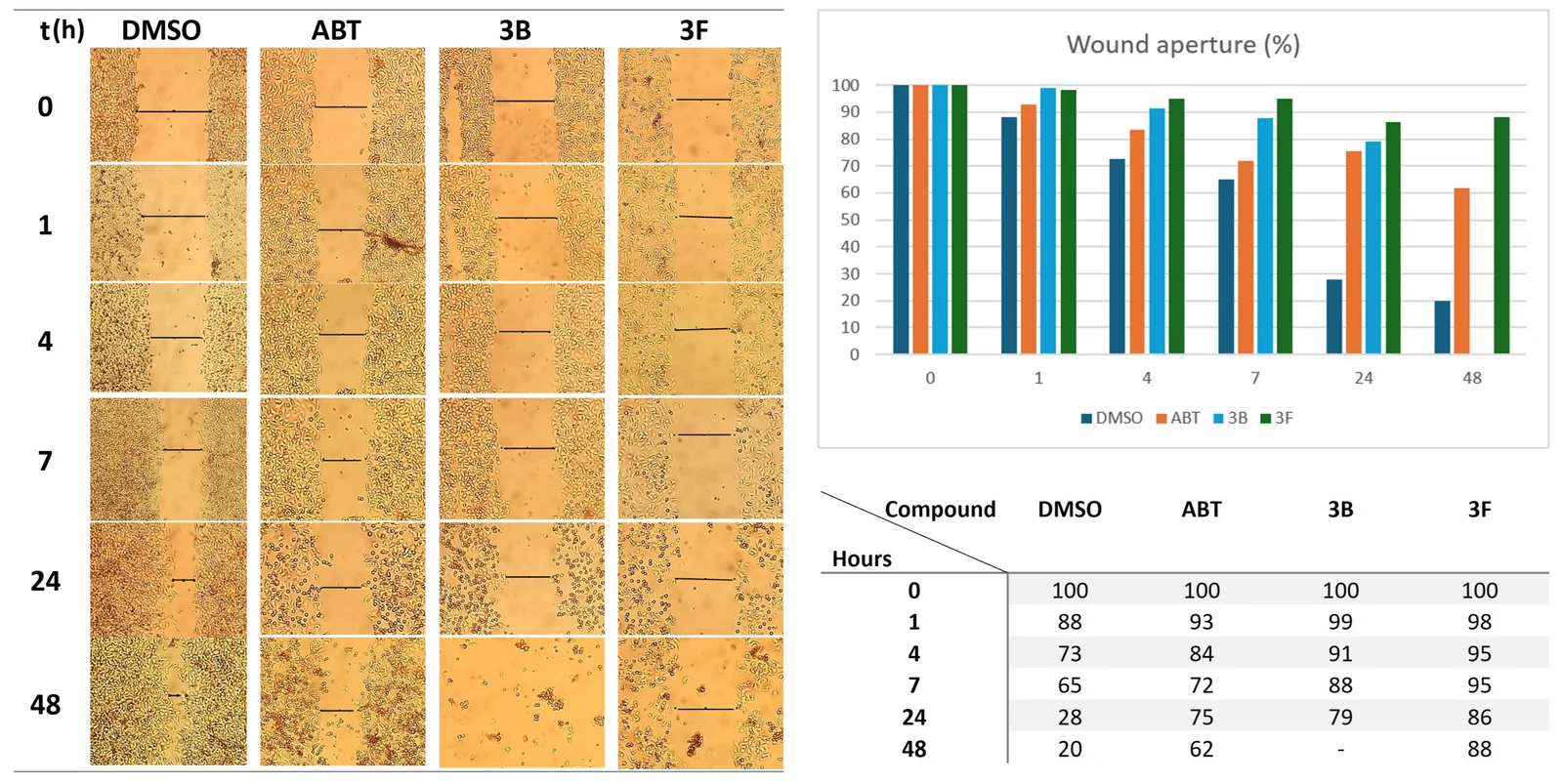
Left: Time course of the closing of wounds, indicated by a black bar, in HeLa monolayers after treatment with 0.1% DMSO (Ctrl), and 0.5 µM ABT-751, 0.5 µM **3B**, and 0.5 µM **3F**. Right: Bar graph (up) and values (down) for the remaining wound aperture at the indicated time points after treatments.

In this assay, HeLa cells are allowed to grow up to confluence, and then a scratch is made, thus generating a cell-free gap. Then, the compounds are added at the desired concentration, and pictures are taken at different intervals (Figure 5). Quantification of the closure of the gap with time gives an estimation of the migration rate of the cells.

ABT-751 and compounds **3B** and **3F** slow down the closure of the wound compared to the untreated control, which at 48 h almost completely healed. This suggests that the three sulfonamides are able to impair cell migration at the tested concentration. **3B** and **3F** show larger migration inhibitory effects than ABT-751, although **3B** causes a large cell detachment at the final (48 h) time point. It is noteworthy that **3F**, at this concentration lower than its IC_50_ value against HeLa cells (0.64 µM), maintains the gap mostly open at this time.

c. **In silico studies**
  - ***Conformational analysis***

*N*-trimethoxyphenyl naphthalene sulfonamides are moderately flexible compounds with conformations arising from rotations about the sulfonamide (S-N) bond, the 2-naphthyl—sulfur and phenyl-nitrogen single bonds, and the aromatic methoxy groups. We will refer to this common substructure of compounds **1–7** as the naphthalene sulfonamide, whose conformational preferences determine the possibility of binding to the different subpocket combinations of the colchicine site of tubulin, which require different relative dispositions of the rings due to the geometrical arrangements in the site. Binding to the AB zones requires a cisoid (as a matter of fact, one of the many possible cisoid dispositions) naphthalene sulfonamide to put the two aryl rings in a close, non-coplanar arrangement. The *N*-substituted naphthalene sulfonamides show a clear conformational preference for these cisoid dispositions of the aromatic rings. The spacers on the sulfonamide nitrogen greatly increase the conformational possibilities of the compounds, more so as they become longer. The all-carbon spacers show preferentially anti-dispositions, whereas the oxygen-containing spacers show an increased tendency towards gauche dispositions along the chain, thus enhancing the conformational variability (Supplementary Material: Supplementary Figure S2, SF2).

  ii. ***Docking experiments***

**Figure 6:**
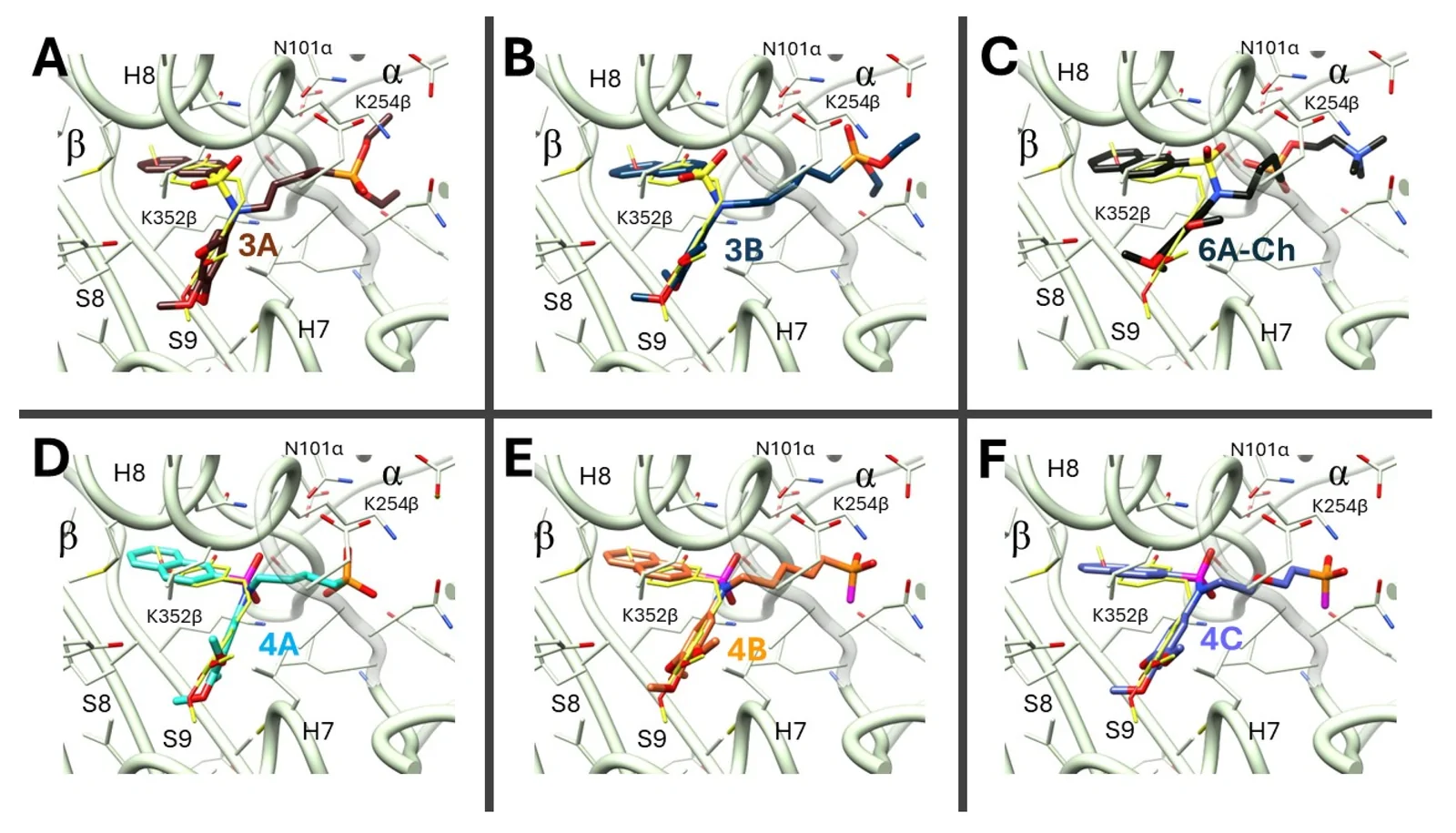
Docking poses for compounds **3A** (**A**), **3B** (**B**), **6A-Ch** (**C**), **4A** (**D**), **4B** (**E**), and **4C** (**F**) in complex with tubulin (PDB ID code 5XAG). The secondary structural elements of the colchicine site are indicated, and the interacting side chains are shown. Combretastatin A4 is shown in thin yellow sticks to assist in the allocation of zones I and II and to facilitate the comparison.

The chemical structure of the compounds resembles that of colchicine-site binders, and therefore the putative binding of the synthesized compounds at the colchicine site of tubulin (Figure 6) was studied by docking experiments. Ligand flexibility was explicitly accounted for by the docking programs. The flexibility of the target was accounted for by docking the ligands in many binding site arrangements, the so-called ensemble docking approach [49], found in the available X-ray crystal structures of tubulin in complex with colchicine site ligands that occupy the different binding sub-pockets (sub-sites A, B, and C) in the PDB [30,31,50]. These ligands can vary greatly in size and nature, even among structurally related ligands, and they induce changes in the corresponding subpockets that sample diverse dispositions [21,30,31]. These configurations are increased by separately considering binding sites with water molecules deemed important for binding, along with “dry” pockets [49], giving 147 models coming from X-ray crystal structures [50]. Additionally, six models representative of the main clusters of a molecular dynamics simulation of tubulin in complex with indole isocombretastatins [51] were included for a final sum of 153 models [49].

AutoDock 4.2 [52] and PLANTS [53] were the two docking programs with different scoring functions used for the docking experiments, looking for complementary exploration and scoring for each ligand binding pose. The best-scoring poses from each software that were similar to each other were selected as the binding mode(s). To compare the binding poses, each pose was assigned as binding to the different sub-pockets by a semiautomatic process that involves several steps: 1) if the shortest distance of any atom of the pose to the geometrical centre of the subpocket, as previously defined in ensemble pharmacophore docking experiments [49], was bellow a threshold, then the pose was assigned as binding to that sub-pocket; 2) the RMSDs between the poses and undecorated scaffolds placing the centroids of phenyl rings at the above mentioned centers of the A, B, or C sub-sites or the X-ray coordinates of reference ligands such as of combretastatin A-4 (PDB ID 5LYJ, A-B sites), MI-181 (PDB ID 4YJ2, A-C sites), or ABT-751 (PDB ID 3HKC, A-B-C sites) [27,30,31,50]. Every pose of all the ligands is allocated in this way to the binding site sub-pockets. The energy scores of the poses of each ligand were scaled to a relative scale between 0 (worse scored) and 1 (best scored), and their Z-scores were calculated. These values allowed us to compare the scores of the two programs. The pair of similar poses with the best possible scores for the two software was taken as the binding mode. Application of this protocol to ligands with known X-ray structures retrieved, in most cases, the correct binding modes [27].

The consensus poses for all the ligands placed the *N*-trimethoxy-2-naphthalene sulfonamide portion of the molecules within the BA sub-pockets of the colchicine site (Figure 6), with the 3,4,5-trimethoxyphenyl ring in the A zone and the naphthalene in the B zone, contacting tubulin as previously described for naphthalene sulfonamides [32]. The spacer projects towards the interfacial space of the tubulin dimer, as designed, and the hydrogen-bond-accepting atoms of the polar capping groups are placed within hydrogen-bond distances of residues of α and β tubulin in the interdimer interface. The phosphonates put the oxygen atom doubly bonded to the phosphorus within a hydrogen-bond distance of the side chains’ NH of K254β or N101α, except for the shorter four-carbon spacer, which does not reach this far and hydrogen bonds instead to the backbone NH of N249β of the T7 loop. The phosphonic acids place the negatively charged oxygens to make hydrogen bonds with the NHs of the side chains of K254β, K352β, or N101α. The charged head groups of the choline derivative **6A-Ch** and the diethylamoniumpropyl groups of **6B-DEAP** and **6D-DEAP** are placed close to the negatively charged phosphates of the GTP at the non-exchangeable site of the α subunit, and the negatively charged carboxylate of E71α; the DEAP derivatives hydrogen-bond to it with their protonated ammonium.

The docking of ligands into multiple protein structures selects the most favorable poses for each ligand but also picks some protein structures preferentially (Supplementary Material: Supplementary Table S10, ST10), reflecting the required adaptations of the target to the structural particularities of the ligands. The PDB IDs most frequently retrieved for the naphthalene sulfonamides with polar caps differ from those previously shown to be selected when shorter and less polar substituents are placed at the sulfonamide nitrogen atom [32]. The procedure selects instead proteins with T7 loop dispositions opening the gorges for the spacer, mostly associated with X-ray ligands with larger bridges between the A and B binding moieties, and mainly directing the spacer towards the interfacial space in the direction towards N101α, while maintaining A and B zones bound by moieties of similar shapes as those of *N*-3,4,5-trimethoxyphenyl naphthalene sulfonamides (e.g., 5XAF and 5XAG for AD4, with bulky β-lactams as bridges, or 6GJ4 for PLANTS, with a pyridine bridge). As previously seen, the two software programs select different proteins, a selectivity attributed to the sought-after differences in scoring, justifying the application of the ensemble docking strategy combined with consensus scoring. The docking results are in good agreement with the design hypothesis that the active ligands bind with the naphthalene in zone A and the 3,4,5-trimethoxyphenyl in zone B, with the sulfonamide bridge opening towards the interfacial space and directing the cap groups towards the polar surface of the α and β subunits at the interfacial space.

  iii. ***Properties***

The SwissADME web tool [54] (Supplementary Material: Supplementary Table S11, ST11) was used to predict physicochemical properties, pharmacokinetic parameters, and drug-likeness predictions for the compounds. All the compounds show fractions of Csp^3^ atoms well above the target of 0.25, TPSA above 100 in all cases, a good predictor of low nonspecific toxicity [55], with most above 130 (although the calculated geometries suggest that part of the polar surfaces might not be totally exposed), and moderate to poor solubilities. None of the compounds is predicted to be CNS penetrant; all have low gastrointestinal (GI) absorption, but **4C** has high. All the compounds are classified as substrates of P-glycoprotein, although our experimental results of co-treatment with verapamil only show clear P-gp efflux for **3D**, **4B**, **6B-DEAP**, and possibly for **4C**. Overall, these results suggest that the molecules are good candidates for antitumor treatment. Although P-gp is considered an efflux system mainly for hydrophobic neutral or cationic substrates, the efflux of anionic substrates, such as the anionic phosphonates coming from phosphonic acid deprotonation, has also been observed. As already mentioned, the charged oxygens are partially hidden from the molecular surface by intramolecular interactions with the polar aromatic CHs.

The sites of potential metabolic transformations by CYP1A2, CYP2C9, CYP2C19, CYP2D6, CYP3A4, and UGT were predicted by SOMP [56] and FAME3 and GLORYx (within NERDD) [57] (Supplementary Material: Supplementary Table S12, ST12). For the phosphonic acids, the main metabolic sites of transformation were oxidative hydroxylation of the phosphonate carbon, followed by hydroxylation of the methoxy groups and subsequent *O*-demethylation. For the diethyl phosphonates, the most likely metabolic transformations are phosphonate hydrolysis and hydroxylation of the phosphonate carbon, both mediated by aliphatic hydroxylation. The four-carbon atom spacer significantly protects 3A from these metabolic transformations, and the presence of oxygen atoms on the phosphonate moderately reduces the probability of these reactions. The partially hydrolyzed ethyl and amino alcohol hydrogenophosphonates mainly suffer hydroxylation of the phosphonate carbon or hydrolysis of the phosphonate ester bonds.

## 3. Methods

- **Chemistry**
  - **General chemical techniques**

Purchased reagents were used as received. We dried and stored solvents over molecular sieves. Precoated silica gel polyester plates (0.25 mm thickness) with a UV fluorescence indicator 254 (Polychrom SI F254, Merck KGaA, Darmstadt, Germany) were used for thin-layer chromatography (TLC). Preparative TLC and flash silica gel column chromatography (Kieselgel 40, 0.040–0.063 mm; Merck KGaA, Darmstadt, Germany) were eluted with the indicated mobile phase mixtures. NMR spectra were run on a Bruker (Billerica, MA, USA) SY spectrometer operating at 400 MHz for ^1^H and 100 MHz for ^13^C with the samples dissolved in CDCl_3_, CD_3_OD, or DMSO-d_6_, as indicated. DEPT-135 and DEPT-90 experiments were used to distinguish C, CH, CH_2_, and CH_3_ carbon signals. Chemical shifts (δ) are in ppm relative to the residual solvent signals. High-resolution mass spectra (HRMS) were performed with a hybrid QSTAR XL quadrupole/time-of-flight (QTOF) mass spectrometer (Agilent Technologies Inc., Santa Clara, CA, US).

  ii. **Chemical synthesis**
    1. **General procedures**
      - **General procedure a for the formation of the sulfonamide**

To a stirred solution of the aniline (1.3–2 eq) in 50 mL of CH_2_Cl_2_ and pyridine (400 µL), the corresponding sulfonyl chloride (1 eq) was slowly added. The mixture was stirred at room temperature for 24 h. After that, the reaction was washed with 2N HCl, 5% NaHCO_3,_ and brine until neutral pH. The organic layers were dried over anhydrous Na_2_SO_4_, filtered, and evaporated under reduced pressure. Crystallization or flash column chromatography afforded the sulfonamides.

      b. **General procedure b for the alkylation of the sulfonamide bridge**

KI was added to a stirred solution of the halogen derivative (4 eq) at room temperature in CH_3_CN (25 mL), and after 5 min, a solution of the sulfonamide (1 eq) in CH_3_CN (25 mL) and Cs_2_CO_3_ (2.5 eq) was added dropwise to the first one. The mixture was stirred under an Ar atmosphere at room temperature for 48 to 72 h or refluxed for 24 h. After that, the reaction was evaporated under reduced pressure, dissolved in EtOAc, and washed with brine until neutral pH. The organic layers were dried over anhydrous Na_2_SO_4_, filtered, and evaporated under reduced pressure. Flash column chromatography and/or crystallization afforded the products.

      c. **General procedure c for the introduction of diethyl phosphonates**

A stirred solution of the alkyl halide (1 eq) in 1.5 mL of triethyl phosphite was refluxed at 150 °C for 24 h. After that, the mixture was precipitated with 50 mL of hexane 3 times, and flash column chromatography, crystallization, or precipitation afforded the diethyl phosphonates.

      d. **General procedure d for the formation of phosphonic acids**

To a stirred solution of the corresponding diethyl phosphonate (1 eq) in 3 mL of DMF, NaBr (4 eq) and TMS-Cl (4 eq) were added. The solution was stirred at 75 °C for 18 h in a sealed tube. After that, 3 mL of 2N HCl was added, and the solution was stirred for 5 min at room temperature until the formation of a precipitate. The precipitate was redissolved in EtOAc, and the aqueous layer was extracted two more times with EtOAc. The organic layers were washed 5 times with brine, dried over anhydrous Na_2_SO_4_, filtered, and evaporated under reduced pressure. Precipitation, flash column chromatography, and/or crystallization afforded the products.

      e. **General procedure e for the formation of diethyl tiophosphonates**

The corresponding diethyl phosphonate (1 eq) was dissolved in 20 mL of toluene and, under stirring, Lawesson’s reagent (1.1 eq) was added to the solution. The reaction was stirred at 120 °C under an Ar atmosphere for 24 h. After that, evaporation of the reaction under reduced pressure and flash column chromatography or crystallization afforded the diethyl tiophosphonates.

      f. **General procedure f the formation of phosphonic acid esters**

To a stirred solution of the corresponding phosphonic acid (1 eq) in 25 mL of CH_2_Cl_2_, DMAP (1.6 eq) and EDC (2.3 eq) were added, and the solution was stirred at room temperature for 5 min. After that, the alcohol (1.1 eq) was added, and the solution was refluxed for 24 h under an Ar atmosphere. The reaction was then poured onto ice and carefully washed with brine until neutral pH. The organic layers were dried over anhydrous Na_2_SO_4_, filtered, and evaporated under reduced pressure. Flash column chromatography and/or precipitation afforded the phosphonates.

    2. **Synthesis and characterization of the compounds**
      - **Synthesis of N-(3,4,5-trimethoxyphenyl)naphthalene-2-sulfonamide (1)**

Following the general procedure, 3,4,5-trimethoxyaniline (1.72 g, 9.40 mmol) and naphthalene-2-sulfonyl chloride (1.979 g, 8.73 mmol) gave a white solid that was purified by crystallization to afford 2.483 g (6.65 mmol, 76%) of **1** as white crystals.

***N*****-(3,4,5-trimethoxyphenyl)naphthalene-2-sulfonamide (1): ^1^H-NMR (CDCl_3_):** 8.34 (1 H, d, *J* = 2.0 Hz); 7.93–7.87 (3 H, m); 7.73 (1 H, dd, *J* = 8.8 Hz, *J* = 0.6 Hz); 7.64 (1 H, bt, *J* = 7.0 Hz); 7.59 (1 H, bt, *J* = 7.0 Hz); 6.35 (NH, s); 6.28 (2 H, s); 3.75 (3 H, s); 3.68 (6 H, s) ppm. **^13^C-NMR (CDCl_3_):** 153.4 (2 C); 135.7 (2 C); 134.9 (C); 132.3 (C); 132.0 (C); 129.4 (CH); 129.2 (CH); 129.0 (2 CH); 127.9 (CH); 127.7 (CH); 122.9 (CH); 99.9 (2 CH); 60.9 (CH_3_); 56.0 (2 CH_3_) ppm. **M.p. (Hexane/CH_2_Cl_2_):** 161–162 °C. **HRMS:** Calculated for C_19_H_19_NO_5_SNa^+^ 396.0876, found 396.0875 (M + Na^+^).

      b. **Synthesis of N-(4-bromobutyl)-N-(3,4,5-trimethoxyphenyl)naphthalene-2-sulfonamide (2A-Br):**

Following the general procedure **b**, compound **1** (0.892 g, 2.39 mmol) was alkylated with 1,4-dibromobutane (1141 µL, 9.56 mmol), and the reaction was stirred at room temperature for 72 h. The crude was purified by flash column chromatography (hexane/EtOAc 7:3) giving 581 mg (1.11 mmol, 47%) of a mixture of bromine (**2A-Br**, 374 mg, 0.74 mmol, 31%) and iodine (**2A-I**, 207 mg, 0.37 mmol, 16%) derivatives as a white solid and 94 mg (0.12 mmol, 10%) of **2bA** as a white solid. Compounds **2A-Br** and **2A-I** co-crystallized in hexane/CH_2_Cl_2,_ giving 280 mg of white crystals with a **m.p.** of 123–125 °C.

***N*****-(4-bromobutyl)-*****N*****-(3,4,5-trimethoxyphenyl)naphthalene-2-sulfonamide (2A-Br): ^1^H-NMR (CDCl_3_):** 8.18 (1 H, s); 7.92–7.86 (3 H, m); 7.63–7.55 (3 H, m); 6.18 (2 H, s); 3.83 (6 H, s); 3.54 (3 H, s); 3.58–3.54 (2 H, m); 3.42 (2 H, t, *J* = 6.6 Hz); 1.99–1.90 (2 H, m); 1.62–1.55 (2 H, m) ppm. **^13^C-NMR (CDCl_3_):** 153.1 (2 C); 138.0 (C); 134.9 (C); 134.8 (C); 134.3 (C); 132.0 (C); 129.1 (2 CH); 128.9 (2 CH); 127.9 (CH); 127.7 (CH); 123.1 (CH); 106.4 (2 CH); 60.9 (CH_3_); 56.1 (2 CH_3_); 50.0 (CH_2_); 33.2 (CH_2_); 29.4 (CH_2_); 26.6 (CH_2_) ppm. **HRMS:** Calculated for C_23_H_26_BrNO_5_SNa^+^ 530.0607, found 530.0599 (M + Na^+^).

***N*****-(4-iodobutyl)-*****N*****-(3,4,5-trimethoxyphenyl)naphthalene-2-sulfonamide (2A-I): ^1^H-NMR (CDCl_3_):** 8.18 (1 H, s); 7.92–7.86 (3 H, m); 7.63–7.55 (3 H, m); 6.18 (2 H, s); 3.83 (6 H, s); 3.54 (3 H, s); 3.58–3.54 (2 H, m); 3.18 (2 H, t, *J* = 6.8 Hz); 1.99–1.90 (2 H, m); 1.62–1.55 (2 H, m) ppm. **^13^C-NMR (CDCl_3_):** 153.1 (2 C); 138.0 (C); 134.9 (C); 134.8 (C); 134.3 (C); 132.0 (C); 129.1 (2 CH); 128.9 (2 CH); 127.9 (CH); 127.7 (CH); 123.1 (CH); 106.4 (2 CH); 60.9 (CH_3_); 56.1 (2 CH_3_); 49.8 (CH_2_); 30.0 (CH_2_); 28.8 (CH_2_); 6.2 (CH_2_) ppm. **HRMS:** Calculated for C_23_H_26_INO_5_SNa^+^ 578.0469, found 578.0458 (M + Na^+^).

***N*****,*****N*****’-(butane-1,4-diyl)bis(*****N*****-(3,4,5-trimethoxyphenyl)naphthalene-2-sulfonamide) (2bA): ^1^H-NMR (CDCl_3_):** 8.20 (2 H, s); 7.94–7.90 (6 H, m); 7.67–7.60 (6 H, m); 6.17 (4 H, s); 3.84 (6 H, s); 3.58 (12 H, s); 3.65–3.49 (4 H, m); 1.65–1.53 (4 H, m) ppm. **^13^C-NMR (CDCl_3_):** 153.1 (4 C); 137.9 (2 C); 134.8 (4 C); 134.5 (2 C); 132.0 (2 C); 129.2 (4 CH); 128.9 (2 CH); 128.8 (2 CH); 127.8 (2 CH); 127.6 (2 CH); 123.2 (2 CH); 106.4 (4 CH); 60.8 (2 CH_3_); 56.0 (4 CH_3_); 50.1 (2 CH_2_); 25.0 (2 CH_2_) ppm. **M.p. (MeOH):** 215–216 °C.

      c. **Synthesis of N-(5-bromopentyl)-N-(3,4,5-trimethoxyphenyl)naphthalene-2-sulfonamide (2B-Br):**

Following general procedure **b**, compound **1** (1.982 g, 5.30 mmol) was alkylated using 1,5-dibromopentane (2200 µL, 16.29 mmol), and the reaction was stirred at room temperature for 72 h. The crude was purified by flash column chromatography (hexane/EtOAc 7:3) giving 1545 mg (2.90 mmol, 50%) of a mixture of bromine (**2B-Br**, 1214 mg, 2.32 mmol, 44%) and iodine (**2B-I**, 331 mg, 0.58 mmol, 11%) derivatives as a white solid and 251 mg (0.30 mmol, 12%) of **2bB** as a white solid. Compounds **2B-Br** and **2B-I** co-crystallized in hexane/EtOAc, giving 421 mg of white crystals with a **m.p.** of 115–117 °C.

***N*****-(5-bromopentyl)-*****N*****-(3,4,5-trimethoxyphenyl)naphthalene-2-sulfonamide (2B-Br): ^1^H-NMR (CDCl_3_):** 8.20 (1 H, bs); 7.93–7.89 (3 H, m); 7.65–7.58 (3 H, m); 6.19 (2 H, s); 3.83 (3 H, s); 3.60 (6 H, s); 3.53 (2 H, t, *J* = 7.0 Hz); 3.37 (2 H, t, *J* = 6.6 Hz); 1.88–1.77 (2 H, m); 1.56–1.43 (4 H, m) ppm. **^13^C-NMR (CDCl_3_):** 153.1 (2 C); 137.9 (C); 135.0 (C); 134.7 (C); 134.5 (C); 131.9 (C); 129.1 (2 CH); 128.8 (2 CH); 127.8 (CH); 127.6 (CH); 123.2 (CH); 106.4 (2 CH); 60.8 (CH_3_); 56.0 (2 CH_3_); 50.8 (CH_2_); 33.7 (CH_2_); 32.1 (CH_2_); 27.4 (CH_2_); 24.9 (CH_2_) ppm.

***N*****-(5-iodopentyl)-*****N*****-(3,4,5-trimethoxyphenyl)naphthalene-2-sulfonamide (2B-I): ^1^H-NMR (CDCl_3_):** 8.20 (1 H, bs); 7.93–7.89 (3 H, m); 7.65–7.58 (3 H, m); 6.19 (2 H, s); 3.83 (3 H, s); 3.60 (6 H, s); 3.53 (2 H, t, *J* = 7.0 Hz); 3.15 (2 H, t, *J* = 6.8 Hz); 1.88–1.77 (2 H, m); 1.56–1.47 (4 H, m) ppm. 8.15 (1 H, s); 7.89–7.83 (3 H, m); 7.61–7.53 (3 H, m); 6.16 (2 H, s); 3.79 (3 H, s); 3.55 (6 H, s); 3.53 (2 H, t, *J* = 7.0 Hz); 3.15 (2 H, t, *J* = 6.8 Hz); 1.80–1.74 (2 H, m); 1.56–1.43 (4 H, m) ppm. **^13^C-NMR (CDCl_3_):** 153.1 (2 C); 137.9 (C); 135.0 (C); 134.7 (C); 134.5 (C); 131.9 (C); 129.1 (2 CH); 128.8 (2 CH); 127.8 (CH); 127.6 (CH); 123.2 (CH); 106.4 (2 CH); 60.8 (CH_3_); 56.0 (2 CH_3_); 50.8 (CH_2_); 32.8 (CH_2_); 27.3 (CH_2_); 27.2 (CH_2_); 6.5 (CH_2_) ppm.

***N,N*****’-(pentane-1,5-diyl)bis(*****N*****-(3,4,5-trimethoxyphenyl)naphthalene-2-sulfonamide) (2bB): ^1^H-NMR (CDCl_3_):** 8.19 (2 H, s); 7.94–7.90 (6 H, m); 7.67–7.60 (6 H, m); 6.18 (4 H, s); 3.84 (6 H, s); 3.66 (12 H, s); 3.51 (4 H, t, *J* = 6.4 Hz); 1.55–1.48 (6 H, m).

      d. **Synthesis of N-(2-(2-chloroethoxy)ethyl)-N-(3,4,5-trimethoxyphenyl)naphthalene-2-sulfonamide (2C):**

Following general procedure **b**, compound **1** (1.026 g, 2.75 mmol) was alkylated with 1-chloro-2-(2-chloroethoxy)ethane (970 µL, 8.25 mmol), and the reaction was refluxed for 24 h. The crude product was purified by flash column chromatography (hexane/EtOAc 7:3), giving 840 mg (1.75 mmol, 64%) of **2C** as a white solid and 116 mg (0.14 mmol, 5%) of **2bC**, which were crystallized to afford 100 mg (0.12 mmol, 4%) of white crystals.

***N*****-(2-(2-chloroethoxy)ethyl)-*****N*****-(3,4,5-trimethoxyphenyl)naphthalene-2-sulfonamide (2C): ^1^H-NMR (CDCl_3_):** 8.24 (1 H, d, *J* = 1.8 Hz); 7.94–7.87 (3 H, m); 7.69 (1 H, dd, *J* = 8.8 Hz, *J* = 1.8 Hz); 7.63 (1 H, bt, *J* = 7.0 Hz); 7.59 (1 H, bt, *J* = 7.0 Hz); 6.25 (2 H, s); 3.82 (3 H, s); 3.77 (2 H, t, *J* = 6.0 Hz); 3.64 (2 H, t, *J* = 5.7 Hz); 3.59 (6 H, s); 3.61 (2 H, m); 3.51 (2 H, t, *J* = 6.0 Hz) ppm. **^13^C-NMR (CDCl_3_):** 153.1 (2 C); 138.0 (C); 135.4 (C); 134.8 (C); 134.8 (C); 132.0 (C); 129.2 (CH); 129.1 (CH); 128.9 (CH); 128.8 (CH); 127.8 (CH); 127.6 (CH); 123.3 (CH); 106.7 (2 CH); 71.0 (CH_2_); 68.8 (CH_2_); 60.9 (CH_3_); 56.0 (2 CH_3_); 50.8 (CH_2_); 42,8 (CH_2_) ppm. **HRMS:** Calculated for C_23_H_26_ClNO_6_SNa^+^ 502.1062, found 502.1053 (M + Na^+^).

***N*****,*****N*****’-(oxybis(ethane-2,1-diyl))bis(*****N*****-(3,4,5-trimethoxyphenyl)naphthalene-2-sulfonamide) (2bC): ^1^H-NMR (CDCl_3_):** 8.21 (2 H, s); 7.95–7.87 (6 H, m); 7.66 (2 H, dd, *J* = 8.8 Hz, *J* = 2.0 Hz); 7.64 (2 H, m); 7.59 (2 H, dt, *J* = 7.0 Hz, *J* = 1.5 Hz); 6.18 (4 H, s); 3.82 (6 H, s); 3.66 (4 H, t, *J* = 6.3 Hz); 3.56 (12 H, s); 3.50 (4 H, t, *J* = 6.3 Hz) ppm. **^13^C-NMR (CDCl_3_):** 153.1 (4 C); 138.0 (2 C); 135.2 (2 C); 134.8 (4 C); 132.0 (2 C); 129.2 (4 CH); 128.9 (2 CH); 128.8 (2 CH); 127.8 (2 CH); 127.6 (2 CH); 123.2 (2 CH); 106.5 (4 CH); 68.5 (2 CH_2_); 60.9 (2 CH_3_); 56.0 (4 CH_3_); 50.7 (2 CH_2_) ppm. **M.p. (CH_2_Cl_2_/MeOH):** 169–170 °C.

      e. **Synthesis of N-(6-bromohexyl)-N-(3,4,5-trimethoxyphenyl)naphthalene-2-sulfonamide (2D-Br):**

Following general procedure b, compound **1** (1.089 g, 2.92 mmol) was alkylated with 1,6-dibromohexane (1340 µL, 8.75 mmol), and the reaction was stirred at room temperature for 72 h. The crude was purified by flash column chromatography (hexane/EtOAc 7:3) giving 541 mg (0.98 mmol, 33%) of a mixture of bromine (**2D-Br**, 304 mg, 0.57 mmol, 20%) and iodine (**2D-I**, 237 mg, 0.41 mmol, 14%) derivatives as a white solid, and 546 mg (0.66 mmol, 46%) of **2bD** that were crystallized to afford 201 mg (0.24 mmol, 17%) of white crystals. Compounds **2D-Br** and **2D-I** co-crystallized in hexane/EtOAc, giving 113 mg (0.20 mmol, 7%) of white crystals with a **m.p.** of 125–126 °C.

***N*****-(6-bromohexyl)-*****N*****-(3,4,5-trimethoxyphenyl)naphthalene-2-sulfonamide (2D-Br): ^1^H-NMR (CDCl_3_):** 8.19 (1 H, s); 7.92–7.87 (3 H, m); 7.64 (1 H, dd, *J* = 8.6 Hz, *J* = 1.7 Hz); 7.62–7.55 (2 H, m); 6.19 (2 H, s); 3.83 (3 H, s); 3.59 (6 H, s); 3.52 (2 H, t, *J* = 6.8 Hz); 3.35 (2 H, t, *J* = 6.8 Hz); 1.81–1.36 (8 H, m) ppm. **^13^C-NMR (CDCl_3_):** 153.1 (2 C); 137.8 (C); 135.1 (C); 134.7 (C); 134.6 (C); 132.0 (C); 129.1 (2 CH); 128.8 (2 CH); 127.9 (CH); 127.6 (CH); 123.2 (CH); 106.4 (2 CH); 60.9 (CH_3_); 56.1 (2 CH_3_); 50.9 (CH_2_); 33.7 (CH_2_); 32.5 (CH_2_); 28.1 (CH_2_); 27.6 (CH_2_); 25.5 (CH_2_) ppm. **HRMS:** Calculated for C_25_H_30_BrNO_5_SNa^+^ 558.0920, found 558.0919 (M + Na^+^).

***N*****-(6-iodohexyl)-*****N*****-(3,4,5-trimethoxyphenyl)naphthalene-2-sulfonamide (2D-I): ^1^H-NMR (CDCl_3_):** 8.19 (1 H, s); 7.92–7.87 (3 H, m); 7.64 (1 H, dd, *J* = 8.6 Hz, *J* = 1.7 Hz); 7.62–7.55 (2 H, m); 6.19 (2 H, s); 3.83 (3 H, s); 3.59 (6 H, s); 3.52 (2 H, t, *J* = 6.8 Hz); 3.13 (2 H, t, *J* = 7.0 Hz); 1.81–1.36 (8 H, m) ppm.**^13^C-NMR (CDCl_3_)**: 153.1 (2 C); 137.8 (C); 135.1 (C); 134.7 (C); 134.6 (C); 132.0 (C); 129.1 (2 CH); 128.8 (2 CH); 127.9 (CH); 127.6 (CH); 123.2 (CH); 106.4 (2 CH); 60.9 (CH_3_); 56.1 (2 CH_3_); 50.9 (CH_2_); 33.2 (CH_2_); 29.9 (CH_2_); 28.1 (CH_2_); 25.3 (CH_2_); 6.98 (CH_2_) ppm.

***N*****,*****N*****’-(hexane-1,6-diyl)bis(*****N*****-(3,4,5-trimethoxyphenyl)naphthalene-2-sulfonamide) (2bD): ^1^H-NMR (CDCl_3_):** 8.19 (2 H, s); 7.95–7.87 (6 H, m); 7.67–7.56 (6 H, m); 6.18 (4 H, s); 3.83 (6 H, s); 3.58 (12 H, s); 3.50 (4 H, t, *J* = 6.9 Hz); 1.50–1.30 (8 H, m) ppm. **^13^C-NMR (CDCl_3_):** 153.1 (4 C); 137.9 (2 C); 135.1 (2 C); 134.7 (2 C); 134.6 (2 C); 132.0 (2 C); 129.2 (2 CH); 129.1 (2 CH); 128.8 (4 CH); 127.8 (2 CH); 127.6 (2 CH); 123.2 (2 CH); 106.4 (4 CH); 60.9 (2 CH_3_); 56.0 (4 CH_3_); 50.9 (2 CH_2_); 28.2 (2 CH_2_); 25.9 (2 CH_2_) ppm. **M.p. (MeOH):** 148–149 °C.

      f. **Synthesis of N-(2-(2-(2-chloroethoxy)ethoxy)ethyl)-N-(3,4,5-trimethoxyphenyl)naphthalene-2-sulfonamide (2E):**

Following general procedure b, compound **1** (1.51 g, 4.04 mmol) was alkylated with 1-chloro-2-(2-chloroethoxy)ethane (2536 µL, 16.17 mmol), and the reaction was refluxed for 24 h. The crude product was purified by flash column chromatography (hexane/EtOAc 7:3), giving 1216 mg (2.32 mmol, 57%) of **2E** as a brown oil and 451 mg (0.52 mmol, 26%) of **2bE**, which were crystallized to afford 169 mg (0.20 mmol, 10%) of white crystals.

***N*****-(2-(2-(2-chloroethoxy)ethoxy)ethyl)-*****N*****-(3,4,5-trimethoxyphenyl)naphthalene-2-sulfonamide (2E): ^1^H-NMR (CDCl_3_):** 8.23 (1 H, s); 7.94–7.88 (3 H, m); 7.67 (1 H, dd, *J* = 8.6 Hz, *J* = 1.9 Hz); 7.65 (1 H, m); 7.60 (1 H, t, *J* = 7.0 Hz); 6.23 (2 H, s); 3.83 (3 H, s); 3.76 (2 H, t, *J* = 6.4 Hz); 3.70–3.65 (2 H, m); 3.60 (6 H, s); 3.60–3.55 (8 H, m) ppm. **^13^C-NMR (CDCl_3_):** 153.1 (2 C); 138.0 (C); 135.3 (C); 134.9 (C); 134.8 (C); 132.0 (C); 129.2 (CH); 129.1 (CH); 128.9 (CH); 128.8 (CH); 127.8 (CH); 127.6 (CH); 123.2 (CH); 106.7 (2 CH); 72.5 (CH_2_); 70.4 (CH_2_); 70.3 (CH_2_); 68.8 (CH_2_); 61.7 (CH_2_); 60.9 (CH_3_); 56.0 (2 CH_3_); 50.7 (CH_2_) ppm. **HRMS:** Calculated for C_25_H_29_NO_7_SNa^+^ 546.1324, found 546.1326 (M + Na^+^).

***N*****,*****N*****’-((ethane-1,2-diylbis(oxy))bis(ethane-2,1-diyl))bis(*****N*****-(3,4,5-trimethoxyphenyl)naphthalene-2-sulfonamide) (2bE): ^1^H-NMR (CDCl_3_):** 8.22 (2 H, d, *J* = 1.8 Hz); 7.93–7.85 (6 H, m); 7.68 (2 H, dd, *J* = 8.6 Hz, *J* = 1.9 Hz); 7.64 (2 H, td, *J* = 7.0 Hz, *J* = 1.4 Hz); 7.60 (2 H, td, *J* = 7.0 Hz, *J* = 1.4 Hz); 6.22 (4 H, s); 3.82 (6 H, s); 3.74 (4 H, t, *J* = 6.4 Hz); 3.56 (12 H, s); 3.56–3.52 (4 H, m); 3.50 (4 H, bs) ppm. **^13^C-NMR (CDCl_3_):** 153.1 (4 C); 138.0 (2 C); 135.4 (2 C); 134.9 (2 C); 134.8 (2 C); 132.0 (2 C); 129.2 (2 CH); 129.1 (2 CH); 128.9 (2 CH); 128.8 (2 CH); 127.8 (2 CH); 127.6 (2 CH); 123.2 (2 CH); 106.6 (4 CH); 70.2 (2 CH_2_); 68.8 (2 CH_2_); 60.8 (2 CH_3_); 56.0 (4 CH_3_); 50.7 (2 CH_2_) ppm. **M.p. (Hexane/EtOAc):** 181–183 °C.

      g. **Synthesis of N-(4-(4-chlorobutoxy)butyl)-N-(3,4,5-trimethoxyphenyl)naphthalene-2-sulfonamide (2F):**

Following general procedure b, compound **1** (1.003 g, 2.69 mmol) was alkylated with 1-chloro-4-(4-chlorobutoxy)butane (1163 µL, 8.07 mmol) under reflux for 24 h. The crude product was purified by flash column chromatography (hexane/EtOAc 6:4), giving 974 mg (1.82 mmol, 68%) of **2F** as a white solid and 30 mg (0.03 mmol, 2%) of **2bF** that crystallized as 28 mg (0.03 mmol, 1%) of white crystals.

***N*****-(4-(4-chlorobutoxy)butyl)-*****N*****-(3,4,5-trimethoxyphenyl)naphthalene-2-sulfonamide (2F): ^1^H-NMR (CDCl_3_):** 8.21 (1 H, bs); 7.96–7.89 (3 H, m); 7.69–7.62 (2 H, m); 7.61 (1 H, bt, *J* = 7.0 Hz); 6.20 (2 H, s); 3.84 (3 H, s); 3.61 (6 H, s); 3.56 (2 H, t, *J* = 6.6 Hz); 3.54 (2 H, t, *J* = 6.6 Hz); 3.43–3.38 (4 H, m); 1.90–1.80 (2 H, m); 1.72–1.50 (6 H, m) ppm. **^13^C-NMR (CDCl_3_):** 153.1 (2 C); 137.9 (C); 135.1 (C); 134.7 (C); 134.5 (C); 132.0 (C); 129.1 (2 CH); 128.8 (2 CH); 127.8 (CH); 127.6 (CH); 123.2 (CH); 106.5 (2 CH); 70.1 (CH_2_); 69.9 (CH_2_); 60.9 (CH_3_); 56.0 (2 CH_3_); 50.8 (CH_2_); 44.9 (CH_2_); 29.5 (CH_2_); 27.0 (CH_2_); 26.5 (CH_2_); 25.2 (CH_2_) ppm. **M.p. (Hexane/CH_2_Cl_2_):** 76–77 °C. **HRMS:** Calculated for C_27_H_34_ClNO_6_SNa^+^ 558.1688, found 558.1697 (M + Na^+^).

***N*****,*****N*****’-(oxybis(butane-4,1-diyl))bis(*****N*****-(3,4,5-trimethoxyphenyl)naphthalene-2-sulfonamide) (2bF)**: **^1^H-NMR (CDCl_3_):** 8.19 (2 H, d, *J* = 1.8 Hz); 7.94–7.86 (6 H, m); 7.64 (2 H, dd, *J* = 8.6 Hz, *J* = 1.8 Hz); 7.62 (2 H, td, *J* = 7.0 Hz, *J* = 1.4 Hz); 7.61 (2 H, bt, *J* = 7.0 Hz, *J* = 1.4 Hz); 6.19 (4 H, s); 3.82 (6 H, s); 3.56 (12 H, s); 3.54 (4 H, t, *J* = 6.8 Hz); 3.37 (4 H, t, *J* = 6.3 Hz); 1.65–1.56 (4 H, m); 1.55–1.46 (4 H, m) ppm. **^13^C-NMR (CDCl_3_):** 153.1 (4 C); 137.9 (2 C); 135.1 (2 C); 134.7 (2 C); 134.5 (2 C); 132.0 (2 C); 129.1 (4 CH); 128.8 (4 CH); 127.8 (2 CH); 127.6 (2 CH); 123.2 (2 CH); 106.5 (4 CH); 70.0 (2 CH_2_); 60.9 (2 CH_3_); 56.0 (4 CH_3_); 50.8 (2 CH_2_); 26.5 (2 CH_2_); 25.1 (2 CH_2_) ppm. **M.p. (Hexane/CH_2_Cl_2_):** 142–143 °C.

      h. **Synthesis of diethyl (4-(N-(3,4,5-trimethoxyphenyl)naphthalene-2-sulfonamido)butyl)phosphonate (3A):**

Following general procedure c, a mixture of the compounds **2ABr** (662 mg, 1.30 mmol) and **2AI** (367 mg, 0.66 mmol) was reacted with triethyl phosphite, and the crude product was purified by flash column chromatography (hexane/acetone 4:6) to afford 418 mg (0.74 mmol, 38%) of **3A** as a light brown oil.

**^1^H-NMR (CDCl_3_):** 8.14 (1 H, d, *J* = 2.0 Hz); 7.91–7.81 (3 H, m); 7.62–7.52 (3 H, m); 6.14 (2 H, s); 4.08–3.95 (4 H, m); 3.78 (3 H, s); 3.55 (6 H, s); 3.50 (2 H, t, *J* = 6.8 Hz); 1.73–1.48 (6 H, m); 1.25 (6 H, t, *J* = 7.0 Hz) ppm. **^13^C-NMR (CDCl_3_):** 153.1 (2 C); 137.9 (C); 134.9 (C); 134.7 (C); 134.4 (C); 132.0 (C); 129.1 (2 CH); 128.8 (2 CH); 127.8 (CH); 127.6 (CH); 123.1 (CH); 106.3 (2 CH); 61.5 (2 CH_2_, d, *J* = 6.5 Hz); 60.8 (CH_3_); 56.0 (2 CH_3_); 50.3 (CH_2_); 28.9 (CH_2_, d, *J* = 15.7 Hz); 25.0 (CH_2_, d, *J* = 141.1 Hz); 19.4 (CH_2_, d, *J* = 5.1 Hz); 16.4 (2 CH_3_, d, *J* = 6.0 Hz) ppm. **HRMS:** Calculated for C_27_H_37_NO_8_PS^+^ 566.1972, found 566.1967 (M + H^+^).

      i. **Synthesis of diethyl (5-(N-(3,4,5-trimethoxyphenyl)naphthalene-2-sulfonamido)pentyl)phosphonate (3B):**

Following general procedure c, a mixture of compound **2BBr** (944 mg, 1.81 mmol) and **2BI** (257 mg, 0.45 mmol) was reacted with triethyl phosphite to afford 1087 mg (1.88 mmol, 83%) of a very clean crude product that was purified by crystallization to afford 95 mg (0.16 mmol, 7%) of **3B** as pale pink crystals.

**^1^H-NMR (CDCl_3_):** 8.16 (1 H, d, *J* = 1.8 Hz); 7.90–7.86 (3 H, m); 7.64–7.58 (2 H, m); 7.56 (1 H, td, *J* = 6.9 Hz, *J* = 1.3 Hz); 6.15 (2 H, s); 4.11–3.97 (4 H, m); 3.81 (3 H, s); 3.57 (6 H, s); 3.49 (2 H, t, *J* = 6.4 Hz); 1.70–1.41 (8 H, m); 1.27 (6 H, t, *J* = 7.0 Hz). **^13^C-NMR (CDCl_3_):** 153.1 (2 C); 137.9 (C); 135.0 (C); 134.7 (C); 134.5 (C); 132.0 (C); 129.1 (2 CH); 128.8 (2 CH); 127.8 (CH); 127.6 (CH); 123.2 (CH); 106.4 (2 CH); 61.4 (CH_2_, d, *J* = 6.5 Hz); 60.9 (CH_3_); 56.0 (2 CH_3_); 50.8 (CH_2_); 27.9 (CH_2_, d, *J* = 1.2 Hz); 27.4 (CH_2_, d, *J* = 17.0 Hz); 25.5 (CH_2_, d, *J* = 140.9 Hz); 22.0 (CH_2_, d, *J* = 5.1 Hz); 16.4 (2 CH_3_, d, *J* = 5.8 Hz) ppm. **M.p. (Hexane):** 82–83 °C. **HRMS:** Calculated for C_28_H_39_NO_8_PS^+^ 580.2129, found 580.2120 (M + H^+^).

      j. **Synthesis of diethyl (2-(2-(N-(3,4,5-trimethoxyphenyl)naphthalene-2-sulfonamido)ethoxy)ethyl)phosphonate (3C):**

Following general procedure c, compound **2C** (752 mg, 1.57 mmol) reacted with triethyl phosphite, and the crude product was purified by flash column chromatography (EtOAc/MeOH 95:5) to afford 604 mg (1.04 mmol, 66%) of **3C** as a brown oil.

**^1^H-NMR (CDCl_3_):** 8.20 (1 H, d, *J* = 2.2 Hz); 7.92–7.85 (3 H, m); 7.65 (1 H, dd, *J* = 1.9, 8.6 Hz); 7.63–7.54 (2 H, m); 6.19 (2 H, s); 4.09–4.00 (4 H, m); 3.80 (3 H, s); 3.72 (2 H, t, *J* = 6.2 Hz); 3.59–3.64 (2 H, m); 3.56 (6 H, s); 3.51 (2 H, t, *J* = 6.2 Hz); 1.96 (2 H, dt, *J* = 7.1, 18.4 Hz); 1.27 (6 H, t, *J* = 7.1 Hz) ppm. **^13^C-NMR (CDCl_3_):** 153.0 (2 C); 137.9 (C); 135.3 (C); 134.6 (2 C); 131.8 (C); 129.0 (CH); 128.8 (2 CH); 128.7 (CH); 127.7 (CH); 127.5 (CH); 123.0 (CH); 106.5 (2 CH); 68.1 (CH_2_); 64.7 (CH_2_); 61.5 (2 CH_2_, d, *J* = 2.4 Hz); 60.6 (CH_3_); 55.8 (2 CH_3_); 50.7 (CH_2_); 26.7 (CH_2_, d, *J* = 139.6 Hz); 16.3 (2 CH_3_, d, *J* = 6.3 Hz) ppm. **HRMS:** Calculated for C_27_H_37_NO_9_PS^+^ 582.1921, found 582.1913 (M + H^+^).

      k. **Synthesis of diethyl (6-(N-(3,4,5-trimethoxyphenyl)naphthalene-2-sulfonamido)hexyl)phosphonate (3D):**

Following general procedure c, a mixture of the compounds **2DBr** (533 mg, 0.99 mmol) and **2DI** (416 mg, 0.71 mmol) was reacted with triethyl phosphite, and the crude product was purified by flash column chromatography (hexane/acetone 4:6) to afford 595 mg (1.00 mmol, 59%) of **3D** as a light brown oil.

**^1^H-NMR (CDCl_3_):** 8.19 (1 H, s); 7.94–7.88 (3 H, m); 7.66–7.61 (2 H, m); 7.59 (1 H, dd, *J* = 7.0 Hz); 6.18 (2 H, s); 4.14–4.00 (4 H, m); 3.83 (3 H, s); 3.59 (6 H, s); 3.51 (2 H, t, *J* = 7.0 Hz); 1.76–1.33 (10 H, m); 1.30 (6 H, bt, *J* = 7.0 Hz) ppm. **^13^C-NMR (CDCl_3_):** 153.2 (2 C); 138.0 (C); 135.2 (C); 134.8 (C); 134.7 (C); 132.1 (C); 129.2 (2xCH); 128.9 (2 CH); 127.9 (CH); 127.7 (CH); 123.3 (CH); 106.5 (2 CH); 61.5 (2 CH_2_, d, *J* = 6.6 Hz); 61.0 (CH_3_); 56.1 (2 CH_3_); 51.1 (CH_2_); 30.1 (CH_2_, d, *J* = 17.0 Hz); 28.2 (CH_2_); 26.0 (CH_2_); 25.7 (CH_2_, d, *J* = 140.7 Hz); 22.5 (CH_2_, d, *J* = 5.2 Hz); 16.5 (2 CH_3_, d, *J* = 5.9 Hz) ppm. **HRMS:** Calculated for C_29_H_41_NO_8_PS^+^ 594.2285, found 594.2275 (M + H^+^).

      l. **Synthesis of diethyl (2-(2-(2-(N-(3,4,5-trimethoxyphenyl)naphthalene-2-sulfonamido)ethoxy)ethoxy)ethyl)phosphonate (3E):**

Following general procedure c, compound **2E** (1023 mg, 1.95 mmol) reacted with triethyl phosphite, and the crude product was purified by flash column chromatography (EtOAc/MeOH 95:5) to afford 394 mg (0.63 mmol, 32%) of **3E** as a brown oil.

**^1^H-NMR (CDCl_3_):** 8.23 (1 H, d, *J* = 1.6 Hz); 7.93–7.89 (3 H, m); 7.67 (1 H, dd, *J* = 8.7, *J* = 1.8 Hz); 7.64 (1 H, td, *J* = 7.0, *J* = 1.7 Hz); 7.60 (1 H, dd, *J* = 7.0, *J* = 1.6 Hz); 6.22 (2 H, s); 4.13–4.02 (4 H, m); 3.82 (3 H, s); 3.75 (2 H, t, *J* = 6.5 Hz); 3.71–3.62 (2 H, m); 3.59 (6 H, s); 3.59–3.48 (6 H, s); 2.08 (2 H, dt, *J* = 7.6; 18.8 Hz); 1.30 (6 H, t, *J* = 7.1 Hz) ppm. **^13^C-NMR (CDCl_3_):** 153.1 (4 C); 138.0 (C); 135.3 (C); 134.9 (C); 134.7 (C); 132.0 (C); 129.2 (CH); 129.1 (CH); 128.9 (CH); 128.8 (CH); 127.8 (CH); 127.6 (CH); 123.2 (CH); 106.6 (2 CH); 70.2 (CH_2_); 70.0 (CH_2_); 68.8 (CH_2_); 65.1 (CH_2_); 61.6 (2 CH_2_, d, *J* = 6.2 Hz); 60.8 (CH_3_); 56.0 (2 CH_3_); 50.7 (CH_2_); 26.9 (CH_2_, d, *J* = 139.3 Hz); 16.4 (2 CH_3_, d, *J* = 6.1 Hz) ppm. **HRMS:** Calculated for C_29_H_40_NO_10_PSNa^+^ 648.2003, found 648.1989 (M + Na^+^).

      m. **Synthesis of diethyl (4-(4-(N-(3,4,5-trimethoxyphenyl)naphthalene-2-sulfonamido)butoxy)butyl)phosphonate (3F):**

Following general procedure c, compound **2F** (1403 mg, 2.62 mmol) reacted with triethyl phosphite, and the crude product was purified by precipitation with hexane to afford 801 mg (1.26 mmol, 48%) of **3F** as a brown oil.

**^1^H-NMR (CDCl_3_):** 8.12 (1 H, d, *J* = 1.9 Hz); 7.87–7.80 (3 H, m); 7.59–7.49 (3 H, m); 6.11 (2 H, s); 4.09–3.92 (4 H, m); 3.75 (3 H, s); 3.51 (6 H, s); 3.48 (2 H, t, *J* = 6.8 Hz); 3.30 (4 H, m); 1.72–1.38 (10 H, m); 1.25 (6 H, t, *J* = 7.0 Hz) ppm. **^13^C-NMR (CDCl_3_):** 153.1 (2 C); 137.9 (C); 135.1 (C); 134.7 (C); 134.5 (C); 132.0 (C); 129.1 (2 CH); 128.8 (CH); 128.7 (CH); 127.8 (CH); 127.6 (CH); 123.2 (CH); 106.4 (2 CH); 70.1 (2 CH_2_); 61.4 (CH_2_, d, *J* = 3.5 Hz); 60.9 (CH_3_); 56.0 (2 CH_3_); 50.8 (CH_2_); 30.5 (CH_2_, d, *J* = 16.4 Hz); 26.5 (CH_2_); 25.5 (CH_2_, d, *J* = 141.1 Hz); 25.1 (CH_2_); 19.3 (CH_2_, d, *J* = 5.0 Hz); 16.4 (CH_3_, d, *J* = 6.0 Hz) ppm. **HRMS:** Calculated for C_31_H_44_NO_9_PSNa^+^ 660.2367, found 660.2355 (M + Na^+^).

      n. **Synthesis of (4-(N-(3,4,5-trimethoxyphenyl)naphthalene-2-sulfonamido)butyl)phosphonic acid (4A):**

Following the general procedure d, compound **3A** (376 mg, 0.66 mmol) was hydrolyzed, and the crude product was purified by crystallization to afford 185 mg (0.36 mmol, 55%) of **4A** as white crystals.

**^1^H-NMR (CD_3_OD):** 8.19 (1 H, d, *J* = 1.8 Hz); 8.04 (1 H, d, *J* = 8.6 Hz); 8.00–7.95 (2 H, m); 7.67 (1 H, t, *J* = 6.9 Hz); 7.64 (1 H, d, *J* = 8.6 Hz); 7.62 (1 H, t, *J* = 6.9 Hz); 6.26 (2 H, s); 3.73 (3 H, s); 3.64 (2 H, t, *J* = 6.6 Hz); 3.54 (6 H, s); 1.80–1.65 (4 H, m); 1.59–1.51 (2 H, m). **^13^C-NMR (CD_3_OD):** 153.1 (2 C); 137.8 (C); 134.9 (C); 134.7 (C); 134.6 (C); 132.1 (C); 128.9 (2 CH); 128.8 (CH); 128.7 (CH); 127.6 (CH); 127.4 (CH); 122.8 (CH); 106.5 (2 CH); 59.8 (CH_3_); 55.1 (2 CH_3_); 50.1 (CH_2_); 28.6 (CH_2_, d, *J* = 16.2 Hz); 26.1 (CH_2_, d, *J* = 138.3 Hz); 19.6 (CH_2_, d, *J* = 4.6 Hz). **M.p. (Hexane/MeOH):** 175–177 °C. **HRMS:** Calculated for C_23_H_27_NO_8_PS^−^ 508.1200, found 508.1199 (M–H^+^).

      o. **Synthesis of (5-(N-(3,4,5-trimethoxyphenyl)naphthalene-2-sulfonamido)pentyl)phosphonic acid (4B):**

Following the general procedure d, compound **3B** (1121 mg, 1.93 mmol) was hydrolyzed, and the crude product was purified by precipitation with EtOAc, giving 565 mg (1.08 mmol, 56%) of **4B,** which was crystallized to afford 208 mg (0.40 mmol, 21%) of white crystals.

**^1^H-NMR (CDCl_3_):** 8.17 (1 H, d, *J* = 1.8 Hz); 7.93–7.86 (3 H, m); 7.65–7.60 (2 H, m); 7.58 (1 H, bt, *J* = 6.9 Hz); 6.16 (2 H, s); 3.81 (3 H, s); 3.57 (6 H, s); 3.54–3.48 (2 H, m); 1.80–1.41 (8 H, m). **^13^C-NMR (CDCl_3_):** 153.1 (2 C); 137.9 (C); 135.0 (C); 134.7 (C); 134.5 (C); 132.0 (C); 129.1 (2 CH); 128.8 (2 CH); 127.8 (CH); 127.6 (CH); 123.2 (CH); 106.4 (2 CH); 60.9 (CH_3_); 56.0 (2 CH_3_); 50.9 (CH_2_); 27.8 (CH_2_); 27.2 (CH_2_, d, *J* = 16.7 Hz); 25.3 (CH_2_, d, *J* = 145.6 Hz); 21.7 (CH_2_, d, *J* = 5.7 Hz) ppm. **M.p. (EtOAc):** 178–179 °C. **HRMS:** Calculated for C_24_H_29_NO_8_PS 522.1357, found 522.1358 (M–H^+^).

      p. **Synthesis of (2-(2-(N-(3,4,5-trimethoxyphenyl)naphthalene-2-sulfonamido)ethoxy)ethyl)phosphonic acid (4C):**

Following general procedure d, compound **3C** (561 mg, 0.96 mmol) was hydrolyzed, giving 506 mg (0.96 mmol, 99%) of a very clean crude product that was purified by crystallization to afford 122 mg (0.23 mmol, 24%) of **4C** as white crystals.

**^1^H-NMR (CDCl_3_):** 8.22 (1 H, d, *J* = 1.8 Hz); 7.95–7.86 (3 H, m); 7.68–7.56 (3 H, m); 7.68 (1 H, d, *J* = 8.6 Hz); 7.63 (1 H, t, *J* = 7.0 Hz); 7.58 (1 H, t, *J* = 7.3 Hz); 6.23 (2 H, s); 3.81 (3 H, s); 3.80–3.68 (4 H, m); 3.57 (6 H, s); 3.58–3.48 (2 H, m); 2.21–2.05 (2 H, m). **^13^C-NMR (CDCl_3_):** 153.2 (2 C); 138.0 (C); 135.1 (C); 134.8 (C); 134.5 (C); 132.0 (C); 129.2 (2 CH); 128.9 (2 CH); 127.8 (CH); 127.6 (CH); 123.1 (CH); 106.7 (2 CH); 68.1 (2 CH_2_); 65.0 (CH_2_); 60.8 (CH_3_); 56.0 (2 CH_3_); 50.9 (CH_2_); 27.3 (CH_2_, d, *J* = 138.5 Hz). **M.p. (Hexane/CHCl_3_):** 152–154 °C. **HRMS:** Calculated for C_23_H_27_NO_9_PS^−^ 524.1150, found 524.1151 (M–H^+^).

      q. **Synthesis of (6-(N-(3,4,5-trimethoxyphenyl)naphthalene-2-sulfonamido)hexyl)phosphonic acid (4D):**

Following the general procedure d, compound **3D** (489 mg, 0.82 mmol) was hydrolyzed, and the crude product was purified by precipitation with diethyl ether, giving 266 mg (0.49 mmol, 60%) of **4D,** which was crystallized to afford 51 mg (0.09 mmol, 11%) of pale brown crystals.

**^1^H-NMR (CDCl_3_):** 8.19 (1 H, d, *J* = 1.8 Hz); 7.94–7.87 (3 H, m); 7.67–7.50 (3 H, m); 6.18 (2 H, s); 3.83 (3 H, s); 3.59 (6 H, s); 3.55–3.47 (2 H, m); 1.90–1.40 (4 H, m); 1.50–1.26 (6 H, m).**^13^C-NMR (CDCl_3_):** 153.1 (2 C); 137.9 (C); 135.1 (C); 134.7 (C); 134.6 (C); 132.0 (C); 129.1 (2 CH); 128.8 (2 CH); 127.8 (CH); 127.6 (CH); 123.2 (CH); 106.4 (2 CH); 60.9 (CH_3_); 56.0 (2 CH_3_); 51.0 (CH_2_); 33.8 (CH_2_); 32.4 (CH_2_); 28.1 (2 CH_2_); 27.0 (CH_2_, d, *J* = 130.0 Hz); 25.9 (CH_2_) ppm. **M.p. (Hexane/CH_2_Cl_2_):** 172–175 °C. **HRMS:** Calculated for C_25_H_31_NO_8_PS^−^ 536.1513, found 536.1510 (M-H^+^).

      r. **Synthesis of (4-(4-(N-(3,4,5-trimethoxyphenyl)naphthalene-2-sulfonamido)butoxy)butyl)phosphonic acid (4F):**

Following general procedure d, compound **3F** (801 mg, 1.26 mmol) was hydrolyzed, giving 578 mg (0.99 mmol, 79%) of a very clean crude that was purified by crystallization to afford 48 mg (0.08 mmol, 6%) of **4F** as white crystals.

**^1^H-NMR (CDCl_3_):** 8.19 (1 H, d, *J* = 1.8 Hz); 7.93–7.98 (3 H, m); 7.65–7.59 (3 H, m); 6.18 (2 H, s); 3.82 (3 H, s); 3.58 (6 H, s); 3.54 (2 H, t, *J* = 6.8 Hz); 3.38 (4 H, m); 1.79–1.49 (10 H, m). **^13^C-NMR (CD_3_OD):** 153.0 (2 C); 137.8 (C); 135.0 (C); 134.7 (C); 134.5 (C); 132.0 (C); 129.1 (2 CH); 128.8 (2 CH); 127.8 (CH); 127.6 (CH); 123.1 (CH); 106.4 (2 CH); 70.2 (CH_2_); 70.1 (CH_2_); 60.8 (CH_3_); 56.0 (2 CH_3_); 50.8 (CH_2_); 30.2 (CH_2_, d, *J* = 17.2 Hz); 26.3 (CH_2_); 25.0 (CH_2_, d, *J* = 145.9 Hz); 19.0 (CH_2_, d, *J* = 5.0 Hz). **M.p. (Hexane/EtOAc):** 93–95 °C. **HRMS:** Calculated for C_27_H_35_NO_9_PS^−^ 580.1776, found 580.1778 (M–H^+^).

      s. **Synthesis of ethyl hydrogen (4-(N-(3,4,5-trimethoxyphenyl)naphthalene-2-sulfonamido)butyl)phosphonate (5A):**

To a stirred solution of compound **3A** (520 mg, 0.92 mmol) in 5 mL of acetone, NaI (140 mg, 0.92 mmol) was added, and the solution was stirred at 100 °C in a sealed tube for 18 h. The crude product was purified by flash column chromatography (EtOAc/MeOH 8:2), giving 496 mg (0.92 mmol, 99%) of **5A** as a white solid.

**^1^H-NMR (CDCl_3_):** 8.19 (1 H, d, *J* = 1.8 Hz); 8.05 (1 H, d, *J* = 8.6 Hz); 8.02–7.97 (2 H, m); 7.70–7.61 (3 H, m); 6.25 (2 H, s); 3.91–3.80 (2 H, m); 3.73 (3 H, s); 3.62 (2 H, t, *J* = 6.9 Hz); 3.55 (6 H, s); 1.70–1.58 (2 H, m); 1.56–1.46 (4 H, m); 1.21 (6 H, t, *J* = 7.0 Hz) ppm. **^13^C-NMR (CD_3_OD):** 153.1 (2 C); 137.8 (C); 135.0 (C); 134.9 (C); 134.8 (C); 132.2 (C); 129.0 (CH); 128.9 (CH); 128.8 (2 CH); 127.7 (CH); 127.5 (CH); 123.0 (CH); 106.5 (2 CH); 59.8 (CH_3_); 59.3 (CH_2_, d, *J* = 5.7 Hz); 55.2 (2 CH_3_); 50.4 (CH_2_); 29.4 (CH_2_, d, *J* = 15.8 Hz); 26.3 (CH_2_, d, *J* = 135.4 Hz); 20.5 (CH_2_, d, *J* = 4.6 Hz); 15.9 (CH_3_, d, *J* = 6.6 Hz) ppm. **HRMS:** Calculated for C_25_H_33_NO_8_PS^+^ 538.1659, found 538.1652 (M + H^+^).

      t. **Synthesis of ethyl hydrogen (6-(N-(3,4,5-trimethoxyphenyl)naphthalene-2-sulfonamido)hexyl)phosphonate (5D):**

A solution of compound **3D** (244 mg, 0.41 mmol) in hydrazine hydrate (8 eq) was refluxed at 130 °C for 24 h. The crude product was purified by flash column chromatography (EtOAc/MeOH 1:1), giving 140 mg (0.25 mmol, 61%) of **5D** as a white solid.

**^1^H-NMR (CD_3_OD):** 8.18 (1 H, d, *J* = 1.8 Hz); 8.00 (1 H, d, *J* = 8.8 Hz); 7.94 (1 H, d, *J* = 8.8 Hz); 7.92 (1 H, d, *J* = 8.8 Hz); 7.68–7.55 (3 H, m); 6.25 (2 H, s); 4.00–3.91 (2 H, m); 3.70 (3 H, s); 3.59 (2 H, t, *J* = 6.3 Hz); 3.52 (6 H, s); 1.67–1.50 (4 H, m); 1.43–1.27 (6 H, m); 1.25 (3 H, t, *J* = 7.0 Hz) ppm. **^13^C-NMR (CD_3_OD):** 153.1 (2 C); 137.8 (C); 135.0 (C); 134.9 (C); 134.7 (C); 132.0 (C); 128.9 (3 CH); 128.8 (CH); 127.7 (CH); 127.5 (CH); 122.9 (CH); 106.5 (2 CH); 60.2 (CH_2_, d, *J* = 5.9 Hz); 59.8 (CH_3_); 55.2 (2 CH_3_); 50.7 (CH_2_); 29.9 (CH_2_, d, *J* = 16.2 Hz); 27.8 (CH_2_); 25.9 (CH_2_, d, *J* = 137.6 Hz); 25.7 (CH_2_); 22.7 (CH_2_, d, *J* = 4.9 Hz); 15.7 (CH_3_, d, *J* = 5.9 Hz) ppm. **HRMS:** Calculated for C_27_H_35_NO_8_PS^−^ 564.1826, found 564.1815 (M–H^+^).

      u. **Synthesis of O,O-diethyl (4-(N-(3,4,5-trimethoxyphenyl)naphthalene-2-sulfonamido)butyl)phosphonothioate (7A):**

Following the general procedure e, compound **3A** (104 mg, 0.18 mmol) reacted with Lawesson’s reagent, giving a crude product that was purified by flash column chromatography (hexane/EtOAc 8:2), giving 43 mg (0.07 mmol, 39%) of **7A,** which was crystallized to afford 6 mg (0.01 mmol, 6%) of white crystals.

**^1^H-NMR (CDCl_3_):** 8.20 (1 H, d, *J* = 1.8 Hz); 7.95–7.89 (3 H, m); 7.67–7.62 (2 H, m); 7.60 (1 H, td, *J* = 6.9 Hz, *J* = 1.3 Hz); 6.19 (2 H, s); 4.17–3.98 (4 H, m); 3.84 (3 H, s); 3.60 (6 H, s); 3.54 (2 H, t, *J* = 7.0 Hz); 1.98–1.88 (2 H, m); 1.74–1.68 (2 H, m); 1.60–1.52 (2 H, m); 1.27 (6 H, t, *J* = 7.0 Hz) ppm. **^13^C-NMR (CDCl_3_):** 153.1 (2 C); 137.9 (C); 134.9 (C); 134.8 (C); 134.4 (C); 132.0 (C); 129.2 (CH); 129.1 (CH); 128.8 (2 CH); 127.8 (CH); 127.6 (CH); 123.2 (CH); 106.4 (2 CH); 60.9 (CH_3_); 62.3 (2 CH_2_, d, *J* = 7.0 Hz); 56.0 (2 CH_3_); 50.5 (CH_2_); 33.9 (CH_2_, d, *J* = 112.2 Hz); 28.8 (CH_2_, d, *J* = 17.2 Hz); 19.9 (CH_2_, d, *J* = 3.2 Hz); 16.2 (2 CH_3_, d, *J* = 6.9 Hz) ppm. **M.p. (Hexane/CH_2_Cl_2_):** 79–80 °C. **HRMS:** Calculated for C_27_H_37_NO_7_PS_2_^+^ 582.1744, found 582.1739 (M + H^+^). White solid.

      v. **Synthesis of O,O-diethyl (5-(N-(3,4,5-trimethoxyphenyl)naphthalene-2-sulfonamido)pentyl)phosphonothioate (7B):**

Following the general procedure e, compound **3B** (113 mg, 0.19 mmol) reacted with Lawesson’s reagent, giving a crude product that was purified by flash column chromatography (hexane/EtOAc 8:2) to afford 55 mg (0.09 mmol, 47%) of **7B** as a white solid.

**^1^H-NMR (CDCl_3_):** 8.19 (1 H, d, *J* = 1.8 Hz); 7.94–7.87 (3 H, m); 7.66–7.61 (2 H, m); 7.59 (1 H, t, *J* = 7.0 Hz); 6.18 (2 H, s); 4.18–3.98 (4 H, m); 3.83 (3 H, s); 3.59 (6 H, s); 3.52 (2 H, t, *J* = 6.4 Hz); 1.96–1.85 (2 H, m); 1.68–1.56 (2 H, m); 1.53–1.41 (4 H, m); 1.28 (6 H, t, *J* = 7.0 Hz) ppm. **^13^C-NMR (CDCl_3_):** 153.0 (2 C); 137.8 (C); 135.0 (C); 134.6 (C); 134.5 (C); 131.9 (C); 129.0 (2 CH); 128.7 (2 CH); 127.7 (CH); 127.5 (CH); 123.1 (CH); 106.3 (2 CH); 62.2 (2 CH_2_, d, *J* = 7.0 Hz); 60.8 (CH_3_); 55.9 (2 CH_3_); 50.8 (CH_2_); 34.3 (CH_2_, d, *J* = 111.8 Hz); 27.8 (CH_2_); 27.0 (CH_2_, d, *J* = 18.4 Hz); 22.4 (CH_2_, d, *J* = 3.7 Hz); 16.1 (2 CH_3_, d, *J* = 6.9 Hz) ppm. **HRMS:** Calculated for C_28_H_39_NO_7_PS_2_^+^ 596.1900, C_28_H_39_NO_7_PS_2_Na^+^ 618.1720, found 596.1893 (M + H^+^), 618.1710 (M + Na^+^).

      w. **Synthesis of O,O-diethyl (6-(N-(3,4,5-trimethoxyphenyl)naphthalene-2-sulfonamido)hexyl)phosphonothioate (7D):**

Following general procedure e, compound **3D** (123 mg, 0.21 mmol) reacted with Lawesson’s reagent, and the crude product was purified by flash column chromatography (hexane/EtOAc 8:2), giving 90 mg (0.15 mmol, 71%) of **7D** as a white solid.

**^1^H-NMR (CDCl_3_):** 8.19 (1 H, d, *J* = 1.8 Hz); 7.94–7.88 (3 H, m); 7.66–7.61 (2 H, m); 7.59 (1 H, t, *J* = 7.0 Hz); 6.18 (2 H, s); 4.19–3.99 (4 H, m); 3.83 (3 H, s); 3.59 (6 H, s); 3.51 (2 H, t, *J* = 6.9 Hz); 1.95–1.85 (2 H, m); 1.66–1.53 (2 H, m); 1.50–1.40 (2 H, m); 1.41–1.32 (4 H, m); 1.28 (6 H, t, *J* = 7.0 Hz) ppm. **^13^C-NMR (CDCl_3_):** 153.0 (2 C); 137.8 (C); 135.0 (C); 134.7 (C); 134.5 (C); 131.9 (C); 129.1 (CH); 129.0 (CH); 128.7 (2 CH); 127.8 (CH); 127.5 (CH); 123.1 (CH); 106.4 (2 CH); 62.2 (2 CH_2_, d, *J* = 7.1 Hz); 60.8 (CH_3_); 56.0 (2 CH_3_); 50.9 (CH_2_); 34.3 (CH_2_, d, *J* = 111.6 Hz); 29.6 (CH_2_, d, *J* = 18.1 Hz); 28.0 (CH_2_); 25.9 (CH_2_, d, *J* = 1.2 Hz); 22.6 (CH_2_, d, *J* = 3.9 Hz); 16.1 (2 CH_3_, d, *J* = 6.9 Hz) ppm. **HRMS:** Calculated for C_29_H_41_NO_7_PS_2_^+^ 610.2057, C_29_H_40_NO_7_PS_2_Na^+^ 632.1868, found 610.2050 (M + H^+^), 632.1876 (M + Na^+^).

      x. **Synthesis of 2-(dimethylamino)ethyl hydrogen (4-(N-(3,4,5-trimethoxyphenyl)naphthalene-2-sulfonamido)butyl)phosphonate (6A-DMAE):**

Following the general procedure f, compound **4A** (110 mg, 0.22 mmol) reacted with 2-(dimethylamino)ethan-1-ol (24 µL, 0.24 mmol). The crude product was purified by flash column chromatography (EtOAc/MeOH 1:9) to afford 58 mg (0.10 mmol, 45%) of **6A-DMAE** as a colorless oil.

**^1^H-NMR (CDCl_3_):** 8.14 (1 H, d, *J* = 1.7 Hz); 7.89–7.84 (3 H, m); 7.63–7.51 (3 H, m); 6.13 (2 H, s); 4.92 (1 H, s); 4.02–3.90 (2 H, m); 3.77 (3 H, s); 3.55–3.51 (2 H, m); 3.51 (6 H, s); 2.69 (2 H, t, *J* = 5.3 Hz); 2.39 (6 H, s); 1.71–1.43 (6 H, m). **^13^C-NMR (CDCl_3_):** 153.0 (2 C); 137.8 (C); 134.9 (C); 134.7 (C); 134.5 (C); 132.0 (C); 129.2 (CH); 129.1 (CH); 128.8 (2 CH); 127.8 (CH); 127.5 (CH); 123.1 (CH); 106.5 (2 CH); 60.8 (CH_3_); 60.5 (CH_2_, d, *J* = 5.2 Hz); 59.4 (CH_2_, d, *J* = 6.4 Hz); 56.0 (2 CH_3_); 50.8 (CH_2_); 44.8 (2 CH_3_); 29.5 (CH_2_, d, *J* = 16.9 Hz); 26.2 (CH_2_, d, *J* = 136.0 Hz); 20.2 (CH_2_, d, *J* = 2.6 Hz). **HRMS:** Calculated for C_27_H_36_N_2_O_8_PS^−^ 579.1935, found 579.1936 (M–H^+^).

      y. **Synthesis of 2-(dimethylamino)ethyl hydrogen (2-(2-(N-(3,4,5-trimethoxyphenyl)naphthalene-2-sulfonamido)ethoxy)ethyl)phosphonate (6C-DMAE):**

Following the general procedure f, compound **4C** (141 mg, 0.27 mmol) reacted with 2-(dimethylamino)ethan-1-ol (30 µL, 0.30 mmol). The crude product was purified by flash column chromatography (EtOAc/MeOH 2:8) and precipitation with EtOAc to afford 58 mg (0.10 mmol, 36%) of **6C-DMAE** as a colorless oil.

**^1^H-NMR (CDCl_3_):** 8.21 (1 H, d, *J* = 1.8 Hz); 7.97–7.89 (3 H, m); 7.69 (1 H, d, *J* = 8.8 Hz); 7.66 (1 H, t, *J* = 7.5 Hz); 7.60 (1 H, t, *J* = 7.5 Hz); 6.19 (2 H, s); 4.34–4.26 (2 H, m); 3.81 (3 H, s); 3.74 (2 H, d, *J* = 6.0 Hz); 3.72–3.61 (2 H, m); 3.57 (6 H, s); 3.50 (2 H, d, *J* = 6.0 Hz); 3.24–3.17 (2 H, m); 2.85 (6 H, s); 2.04–1.92 (2 H, m) ppm. **^13^C-NMR (CDCl_3_):** 153.1 (2 C); 138.0 (C); 135.2 (C); 134.7 (C); 134.6 (C); 132.0 (C); 129.2 (2 CH); 128.9 (2 CH); 127.8 (CH); 127.6 (CH); 123.1 (CH); 106.6 (2 CH); 67.8 (CH_2_); 66.9 (CH_2_); 60.8 (CH_3_); 59.2 (CH_2_, d, *J* = 4.0 Hz); 59.0 (CH_2_, d, *J* = 4.3 Hz); 56.0 (2 CH_3_); 50.9 (CH_2_); 44.2 (2 CH_3_); 28.0 (CH_2_, d, *J* = 133.2 Hz) ppm. **HRMS:** Calculated for C_27_H_36_N_2_O_9_PS^−^ 595.1885, found 595.1888 (M–H^+^).

      z. **Synthesis of 2-(dimethylamino)ethyl hydrogen (4-(4-(N-(3,4,5-trimethoxyphenyl)naphthalene-2-sulfonamido)butoxy)butyl)phosphonate (6F-DMAE):**

Following the general procedure f, compound **4F** (113 mg, 0.19 mmol) reacted with 2-(dimethylamino)ethan-1-ol (22 µL, 0.21 mmol). The crude product was purified by flash column chromatography (EtOAc/MeOH 2:8) and precipitation with EtOAc to afford 40 mg (0.06 mmol, 32%) of **6F-DMAE** as a white solid.

**^1^H-NMR (CDCl_3_):** 8.15 (1 H, d, *J* = 1.8 Hz); 7.92–7.85 (3 H, m); 7.64–7.59 (2 H, m); 7.56 (1 H, td, *J* = 7.0 Hz, *J* = 1.3 Hz); 6.16 (2 H, s); 3.91–3.84 (2 H, m); 3.80 (3 H, s); 3.55 (6 H, s); 3.53 (2 H, t, *J* = 7.0 Hz); 3.37–3.30 (4 H, m); 2.51 (2 H, t, *J* = 5.3 Hz); 2.36 (6 H, s); 1.64–1.44 (10 H, m) ppm. **^13^C-NMR (CDCl_3_):** 153.0 (2 C); 137.9 (C); 135.0 (C); 134.7 (C); 134.5 (C); 132.0 (C); 129.1 (2 CH); 128.8 (2 CH); 127.8 (CH); 127.6 (CH); 123.2 (CH); 106.4 (2 CH); 70.6 (CH_2_); 70.1 (CH_2_); 61.0 (CH_2_, d, *J* = 5.3 Hz); 60.9 (CH_3_); 59.8 (CH_2_, d, *J* = 6.2 Hz); 56.0 (2 CH_3_); 50.9 (CH_2_); 45.1 (2 CH_3_); 31.3 (CH_2_, d, *J* = 16.5 Hz); 26.7 (CH_2_, d, *J* = 134.3 Hz); 26.6 (CH_2_); 25.1 (CH_2_); 20.7 (CH_2_, d, *J* = 4.1 Hz) ppm. **HRMS:** Calculated for C_31_H_46_N_2_O_9_PS^+^ 653.2656, found 653.2646 (M + H^+^).

      aa. **Synthesis of 3-(diethylamino)propyl hydrogen (4-(N-(3,4,5-trimethoxyphenyl)naphthalene-2-sulfonamido)butyl)phosphonate (6A-DEAP):**

Following the general procedure f, compound **4A** (195 mg, 0.38 mmol) reacted with 3-(diethylamino)propan-1-ol (62 µL, 0.42 mmol). The crude product was purified by flash column chromatography (EtOAc/MeOH 2:8) to afford 67 mg (0.11 mmol, 29%) of **6A-DEAP** as a colorless oil.

**^1^H-NMR (CDCl_3_):** 8.12 (1 H, d, *J* = 1.8 Hz); 7.89–7.82 (3 H, m); 7.60 (1 H, dd, *J* = 8.8 Hz, *J* = 1.8 Hz); 7.58 (1 H, m); 7.53 (1 H, td, *J* = 7.0 Hz, *J* = 1.3 Hz); 6.12 (2 H, s); 3.91 (2 H, bq, *J* = 6.7 Hz); 3.76 (3 H, s); 3.51 (6 H, s); 3.43–3.54 (2 H, m); 2.96–2.78 (6 H, m); 1.88 (2 H, quint, *J* = 6.8 Hz); 1.66–1.48 (6 H, m); 1.15 (6 H, t, *J* = 7.2 Hz). **^13^C-NMR (CDCl_3_):** 153.0 (2 C); 137.8 (C); 135.0 (C); 134.7 (C); 134.6 (C); 131.9 (C); 129.1 (CH); 129.0 (CH); 128.8 (2 CH); 127.8 (CH); 127.5 (CH); 123.1 (CH); 106.4 (2 CH); 61.1 (CH_2_, d, *J* = 5.2 Hz); 60.8 (CH_3_); 56.0 (2 CH_3_); 50.9 (CH_2_); 49.1 (CH_2_); 45.7 (2 CH_2_); 29.6 (CH_2_, d, *J* = 15.9 Hz); 26.7 (CH_2_, d, *J* = 135.7 Hz); 26.3 (CH_2_, d, *J* = 4.3 Hz); 21.0 (CH_2_, d, *J* = 4.7 Hz); 9.3 (2 CH_3_) ppm. **HRMS:** Calculated for C_30_H_44_N_2_O_8_PS^+^ 623.2551, found 623.2539 (M + H^+^).

      bb. **Synthesis of 3-(diethylamino)propyl hydrogen (5-(N-(3,4,5-trimethoxyphenyl)naphthalene-2-sulfonamido)pentyl)phosphonate (6B-DEAP):**

Following the general procedure f, compound **4B** (170 mg, 0.32 mmol) reacted with 3-(diethylamino)propan-1-ol (55 µL, 0.37 mmol). The crude product was purified by flash column chromatography (EtOAc/MeOH 5:95) to afford 46 mg (0.07 mmol, 22%) of **6B-DEAP** as a colorless oil.

**^1^H-NMR (CDCl_3_):** 8.14 (1 H, d, *J* = 1.8 Hz); 7.90–7.84 (3 H, m); 7.59 (1 H, dd, *J* = 8.6 Hz, *J* = 1.9 Hz); 7.59 (1 H, m); 7.54 (1 H, td, *J* = 7.0 Hz, *J* = 1.2 Hz); 6.13 (2 H, s); 3.97–3.90 (2 H, m); 3.79 (3 H, s); 3.54 (6 H, s); 3.48 (2 H, t, *J* = 6.7 Hz); 3.04–2.88 (6 H, m); 1.93 (2 H, quint, *J* = 6.5 Hz); 1.59–1.33 (8 H, m); 1.21 (6 H, t, *J* = 7.2 Hz). **^13^C-NMR (CDCl_3_):** 153.0 (2 C); 137.8 (C); 135.1 (C); 134.7 (C); 134.6 (C); 132.0 (C); 129.1 (CH); 129.0 (CH); 128.8 (CH); 128.7 (CH); 127.8 (CH); 127.5 (CH); 123.2 (CH); 106.4 (2 CH); 60.8 (CH_2_, d, *J* = 5.5 Hz); 60.8 (CH_3_); 56.0 (2 CH_3_); 51.1 (CH_2_) 49.0 (CH_2_); 45.5 (2x CH_2_); 28.1 (CH_2_); 27.9 (CH_2_, d, *J* = 16.6 Hz); 25.7 (CH_2_, d, *J* = 4.8 Hz); 27.3 (CH_2_, d, *J* = 136.1 Hz); 23.6 (CH_2_, d, *J* = 4.5 Hz); 8.7 (2 CH_3_) ppm. **HRMS:** Calculated for C_31_H_46_N_2_O_8_PS^+^ 637.2707, found 637.2695 (M + H^+^).

      cc. **Synthesis of 3-(diethylamino)propyl hydrogen (6-(N-(3,4,5-trimethoxyphenyl)naphthalene-2-sulfonamido)hexyl)phosphonate (6D-DEAP):**

Following general procedure f, compound **4D** (149 mg, 0.28 mmol) reacted with 3-(diethylamino)propan-1-ol (45 µL, 0.31 mmol). The crude product was purified by flash column chromatography (EtOAc/MeOH 5:95) to afford 38 mg (0.06 mmol, 21%) of **6D-DEAP** as a colorless oil.

**^1^H-NMR (CDCl_3_):** 8.15 (1 H, d, *J* = 1.8 Hz); 7.92–7.84 (3 H, m); 7.63–7.57 (2 H, m); 7.56 (1 H, bt, *J* = 7.0 Hz); 6.14 (2 H, s); 3.98–3.88 (2 H, m); 3.79 (3 H, s); 3.55 (6 H, s); 3.47 (2 H, t, *J* = 7.0 Hz); 2.98 (2 H, t, *J* = 7.0 Hz); 2.92 (4 H, q, *J* = 7.2 Hz); 1.92 (2 H, quint, *J* = 6.6 Hz); 1.57–1.45 (4 H, m); 1.45–1.36 (2 H, m); 1.36–1.25 (4 H, m); 1.20 (6 H, t, *J* = 7.2 Hz). **^13^C-NMR (CDCl_3_):** 153.0 (2 C); 137.8 (C); 135.1 (C); 134.7 (C); 134.6 (C); 132.0 (C); 129.1 (2 CH); 128.8 (2 CH); 127.8 (CH); 127.6 (CH); 123.2 (CH); 106.4 (2 CH); 61.0 (CH_2_, d, *J* = 5.4 Hz); 60.8 (CH_3_); 56.0 (2 CH_3_); 51.1 (CH_2_); 49.1 (CH_2_); 45.6 (2 CH_2_); 30.6 (CH_2_, d, *J* = 16.8 Hz); 28.2 (CH_2_); 27.2 (CH_2_, d, *J* = 135.5 Hz); 26.1 (CH_2_); 25.9 (CH_2_, d, *J* = 2.4 Hz); 23.8 (CH_2_, d, *J* = 4.1 Hz); 8.8 (2 CH_3_) ppm. **HRMS:** Calculated for C_32_H_48_N_2_O_8_PS^+^ 651.2864, found 651.2853 (M + H^+^).

      dd. **Synthesis of 2-((hydroxy(4-(N-(3,4,5-trimethoxyphenyl)naphthalene-2-sulfonamido)butyl)phosphoryl)oxy)-N,N,N-trimethylethan-1-aminium (6A-Ch):**

Following general procedure f, compound **4A** (61 mg, 0.12 mmol) reacted with 2-hydroxy-*N*,*N*,*N*-trimethylethan-1-aminium hydrochloride (19 mg, 0.13 mmol). The crude product was purified by precipitation with hexane and preparative thin-layer chromatography (MeOH 100%) to afford 7 mg (0.01 mmol, 8%) of **6A-Ch** as a colorless oil.

**^1^H-NMR (CDCl_3_):** 8.18 (1 H, d, *J* = 1.6 Hz); 8.05 (1 H, d, *J* = 8.7 Hz); 8.02–7.98 (2 H, m); 7.71–7.62 (3 H, m); 6.24 (2 H, s); 4.22 (2 H, m); 3.81 (3 H, s); 3.64 (2 H, t, *J* = 6.6 Hz); 3.62–3.59 (2 H, m); 3.55 (6 H, s); 3.22 (9 H, s); 1.70–1.50 (6 H, m) ppm. **HRMS:** Calculated for C_28_H_40_N_2_O_8_PS^+^ 595.2238, found 595.2230 (M + H^+^).

b. **Biology**
  - **Cell Lines and Cell Culture Conditions**

HeLa (human cervical carcinoma), MCF7 (human breast carcinoma), HT-29 (human colorectal adenocarcinoma), U87 (human glioblastoma), and HEK-293 (human embryonic kidney cells) cell lines were purchased from ATTC (Manassas, VA, USA). They were cultured in GlutaMAX™ Dulbecco’s Modified Eagle’s Medium (DMEM) (Gibco, Thermo Fisher Scientific, Grand Island, NY, USA) containing 10% (*v*/*v*) heat-inactivated fetal bovine serum (HIFBS) (Lonza-Cambrex, Karlskoga, Sweden) and 1% streptomycin-penicillin (Sigma-Aldrich, St. Louis, MO, USA) and incubated at 37 °C in 95% humid air and 5% CO_2_.

  ii. **Cell Proliferation Assay**

The proliferation in all cell lines, when treated with the corresponding compounds, was determined using MTT (3-(4,5-dimethylthiazol-2-yl)-2,5-diphenyl-2H-tetrazolium bromide) (Sigma-Aldrich, St. Louis, MO, USA). MTT in Phosphate Buffered Saline (PBS) (5 mg/mL) was added to cells (10 µL/well in 96-well plates; total volume of 120 µL/well). After 4 h of incubation, the medium was aspirated, and formazan violet crystals were dissolved in dimethyl sulfoxide (DMSO) (Sigma- Aldrich, St. Louis, MO, USA) (100 µL/well). Absorbance was measured at 570 nm in a microplate reader (ASYS^®^ UVM-340, connected to DigiRead software).

To determine cell viability, cells in exponential growth phase were seeded (100 µL/well in 96-well plates) with the appropriate cell line concentration (1.5·10^4^ cells/mL for HeLa, 4·10^4^ cells/mL for MCF7 and HT-29, 2·10^4^ cells/mL for HEK-293, and 5·10^4^ cells/mL for U87) in GlutaMAX™ DMEM medium and incubated at 37 °C and 5% CO_2_ atmosphere. After 24 h of incubation to allow cells to attach to the plates, all compounds were added to a final 1 or 10 µM concentration, and the effect on proliferation was evaluated 72 h post-treatment. Compounds showing antiproliferative effects at the evaluated concentration were selected for IC_50_ calculation (50% inhibitory concentration with respect to the untreated controls) from 10^−5^ to 10^−9^ M or 10^−4^ to 10^−8^ M concentrations. Non-linear curve fitting of the experimental data was carried out for each compound, and IC_50_ values were determined using the software GraphPad Prism 8. Compounds were dissolved in DMSO, and the final solvent concentrations never exceeded 0.5% (v/v). The control wells included treated cells with 0.5% (*v/v*) DMSO and the positive control. A final concentration of 10 µM verapamil was included as a control for the HT-29 cell line to study the sensitivity to MDR pumps. Measurements were performed in triplicate, and each experiment was repeated three times.

  iii. **Wound healing assay**

HeLa cells were seeded in 12-well plates at 3·10^5^ cells/well in GlutaMAX™ DMEM medium containing 2% (*v*/*v*) HIFBS and 1% streptomycin-penicillin and incubated at 37 °C in humidified 95% air and 5% CO_2_. After 24 h, using a 200 µL pipette tip, the well was scratched to create a scar in the monolayer and marked with a pen on the outside to track progression over the next 48 h. Each well was carefully washed twice with 0.5 mL of PBS to remove the detached cells, and fresh medium was added with the final concentration of the treatment or DMSO as a negative control. Pictures were taken with a Samsung Galaxy A51 phone from a Motic^®^ inverted light microscope at 0, 1, 4, 7, 24, and 48 h, with a 4x objective. The images were then processed with ImageJ software downloaded from https://imagej.net/ij/ (accessed on 9 January 2026). The healing of the scar was calculated with the following formula:

$$Wound\;aperture\;\left(\%\right)=\;\frac{Area\;at\;x\;hours}{Area\;at\;0\;hours}*100$$

  iv. **Flow cytometry assay: Cell cycle distribution**

Cells were seeded in 6-well plates at the appropriate cell line concentration (5·10^4^ cells/mL for HeLa, 7.5·10^4^ cells/mL for HT-29, and 8.75·10^5^ cells/mL for U87) in GlutaMAX™ DMEM medium containing 10% (*v*/*v*) HIFBS and 1% streptomycin-penicillin and incubated at 37 °C in humidified 95% air and 5% CO_2_. After 24 h, the culture medium was removed, and fresh medium was added with the final concentration of the treatments or DMSO as a negative control. All the samples were performed in triplicate. After 24, 48, and 72 h, cells were detached from the plates with trypsin TrypLE^TM^ Express (Gibco, Thermo Fisher Scientific, Grand Island, NY, USA), the rest recovered with PBS, and centrifuged at 1300 rpm, 24 °C for 10 min. Then, the supernatant was discarded, and the cells within the pellet were fixed with 500 µL of a 7:3 mixture of EtOH/MilliQ H_2_O, then incubated at 4 °C for less than 5 days. 24 h before the experiment, the fixed cells were centrifuged at 1300 rpm, 4 °C for 10 min, resuspended in PBS, and centrifuged again at 1300 rpm, 4 °C for 10 min. The supernatant was discarded, and the pellet was resuspended in 400 µL DNA marking mixture and incubated in the dark at 24 °C.

For each plate, a DNA marking mixture was made of 8 mL of PBS, 8 µL Triton^TM^ X-100 (Sigma-Aldrich, St. Louis, MO, USA), 80 µL RNase A (10 mg/mL) (Roche Diagnostics GmbH, Mannheim, Germany), and 320 µL propidium iodide (1 mg/mL) (Sigma-Aldrich, St. Louis, MO, USA).

A BD Accuri™ C6 Plus Flow Cytometer (BD Biosciences, Franklin Lakes, NJ, USA) was used to analyze the samples, applying a scatter gate (FSC-A vs. SSC-A) gating strategy for 3000 events. Data analysis was performed in BD Accuri™ C6 Software (version 1.0.264.21).

  v. **Apoptosis assay**

Cells were seeded in 12-well plates at the appropriate cell line concentration (5·10^4^ cells/mL for HeLa, 7.5·10^4^ cells/mL for HT-29, and 8.75·10^5^ cells/mL for U87) in GlutaMAX™ DMEM medium containing 10% (*v*/*v*) HIFBS and 1% streptomycin-penicillin and incubated at 37 °C in humid 95% air and 5% CO_2_. After 24 h, the culture medium was removed, and fresh medium was added with the final concentration of the treatments or DMSO as a negative control. All samples were performed in triplicate. After 72 h, cells were detached with TrypLE^TM^ Express and centrifuged at 1500 rpm for 7 min at 24 °C. The supernatant was discarded, and the cells within the pellet were incubated for 15 min in the marking mixture using an Annexin V-FITC/PI apoptosis detection kit (Immunostep, Salamanca, Spain), following the manufacturer’s guidelines.

A BD Accuri™ C6 Plus Flow Cytometer (BD Biosciences, Franklin Lakes, NJ, USA) was used to analyze the samples. The data analysis was performed in BD Accuri™ C6 Software (version 1.0.264.21).

  vi. **Confocal microscopy**

0.01% Poly-L-Lysine pre-coated square glass coverslips (22 mm^2^) were placed in 6-well plates, one coverslip per well. After that, cells were seeded in each well at the appropriate cell line concentration (5·10^4^ cells/mL for HeLa and 8.75·10^5^ cells/mL for U87) in GlutaMAX™ DMEM medium containing 10% (*v*/*v*) HIFBS and 1% streptomycin-penicillin and incubated at 37 °C in humidified 95% air and 5% CO_2_. After 24 h, the culture medium was carefully removed, and fresh medium was added with the final concentration of the treatments or DMSO as a negative control. Cells were incubated for 24 h, after which the medium was removed. Coverslips were washed with PBS, fixed with a 4% formaldehyde (Sigma-Aldrich, St. Louis, MO, USA) in PBS solution and incubated for 10 min, permeabilized with a 0.25% Tryton ™ X-100 in PBS solution for 10 min, blocked with a 10% BSA (Sigma-Aldrich, St. Louis, MO, USA) in PBS solution for 30 min, and finally washed with PBS. After that, a 1:200 anti-α-tubulin mouse monoclonal antibody (Sigma-Aldrich, St. Louis, MO, USA) in PBS solution was added to each coverslip, and they were incubated for 1.5 h. The coverslips were washed again with PBS, and a 1:400 Alexa Fluor 488 goat anti-mouse IgG (Molecular Probes, Invitrogen, Eugene, OR, USA) in PBS solution was added as the secondary antibody and incubated in the dark for 1 h. Coverslips were washed with PBS, and a drop of ProLong™ Gold Antifade Mountant containing DAPI was added for cell nuclei staining, and the samples were incubated for 24 h at 4 °C in the dark before the analysis. The samples were analyzed via confocal microscopy performed on a Leica SP5 microscope DMI-6000V model coupled to a Leica LAS AF software computer.

c. **In silico studies**
  - **Conformational analysis**

The virtual structures of the compounds were built within Spartan’08, and conformers were generated using the conformational search engine at the molecular mechanics (MMFF) level. The conformations were ordered by energy, and the most stable conformers were selected and superimposed.

  ii. **Docking studies**

The virtual compounds were built in Marvin in 2D and transformed to 3D structures [58]. The 3D models were saved as mol2 or pdbqt using AutoDockTools [59]. The virtual compounds were docked into multiple structures representing different configurations of the colchicine site of tubulin (ensemble docking) as described [49]. We downloaded 139 structures, reduced them to a single tubulin dimer, cleaned them to allow docking, and removed the ligands from the binding site. Structures with water bridging the ligands and the protein were used in duplicate, one with the water and the other without it, giving up to 147 structures. We added six models from a previous molecular dynamics simulation, for a total of 153 binding sites. All the refined models were superimposed with Chimera to best superimpose the colchicine sites 6Å from any colchicine site ligand. Docking experiments were run in parallel with AutoDock 4.2 [52] and PLANTS [53]. AutoDock used the Lamarckian genetic algorithm (LGA) 100–300 times for a maximum of 2.5 10^6^ energy evaluations, 150 individuals, and a maximum of 27,000 generations. PLANTS was run 10 instances per ligand with default settings. In-house written scripts and KNIME pipelines [60] were used to semiautomatically process the outputs from the docking programs, allocating the poses to the subzones A, B, C, and D. The programs’ energy scores for each ligand were converted to relative scales from 0 to 1, and Z-scores were calculated. This allowed us to compare the energies of the two software packages. We visually selected the two common poses, one from each program, having the best Z-scores, and took them as the consensus binding mode. LigRMSD [61] calculated RMSDs between the poses and undecorated (with no substituents) model scaffolds and with colchicine binding-site ligands occupying different subzones. Analysis of the poses was done with Chimera [62], OpenEye [63], and JADOPPT [64].

  iii. **Calculation of physicochemical properties, pharmacokinetic parameters, and metabolic transformations**

The structures of the molecules in the most likely protonation states were drawn with ChemDraw and converted to SMILES. The SMILES codes were submitted to the Swiss ADME web tool [54] to obtain their physicochemical properties and pharmacokinetic parameters. Significant properties for the pharmacokinetic behavior of the compounds were compared between compounds: topological polar surface area (TPSA), fraction of sp^3^ carbons, logP, water solubility, GI absorption, BBB penetration, and predicted sensitivity to glycoprotein efflux pumps (Pgp substrate). The predicted sites of metabolism were estimated with the SOMP and NERDD (FAME3 and GLORYx) web tools [56,57].

## 4. Conclusions

Antimitotic drugs binding at the colchicine site of tubulin are potent antitumor agents, but they lack selectivity towards cancer cells, which results in limiting toxicities. Antitumor alkylphospholipids, such as the phospholipid ethers edelfosine and miltefosine, are tumor-selective agents but lack the potency of the antimitotics. Here, we explored the possibility of targeting antimitotic *N*-3,4,5-trimethoxyphenyl naphthalene sulfonamides, novel microtubule-disrupting agents with potent antiproliferative activity against tumor cells [32], towards cancer cells by combining them with a phospholipid moiety in a new approach to small molecule drug conjugates (SMDC). To this end, we synthesized a total of 24 *N*-3,4,5-trimethoxyphenyl naphthalene sulfonamides substituted on the sulfonamide nitrogen with linear spacers of different lengths (4 to 9 atoms) and polarities (alkyls or ethers) and capped with different polar heads (diethyl phosphonates, phosphonic acids, ethyl hydrogenophosphonates, and hydrogenophosphonate esters of ethanol or aminoalcohols (choline, dimethylaminoethanol, or diethylaminopropanol) that mimic the APLs. 23 compounds were assayed against a panel of human cancer cell lines of different origins, revealing that the cap groups determine the antiproliferative activity: diethylphosphonates and some phosphonic acids show similar values of micromolar or submicromolar potencies (IC_50_ values) against HeLa and HT-29 cells; thiophosphonates were less active, with potencies in the micromolar range, and hydrogenophosphonates were all inactive. The potency against HT-29, MCF-7, and U87MG is similar to that against HeLa, and the most potent ones are in the range of ABT-751, a colchicine-site inhibitor that has reached the clinic, used as a positive control. This homogeneous potency against the cell lines is more similar to ABT-751 than to Miltefosine, thus suggesting an antimitotic mechanism of action. The active compounds show IC_50_ values against the non-tumorigenic cell line HEK-293 similar to those observed with ABT-751, with the sole exception of phosphonic acid **4F**, which has a large selectivity index similar to that of miltefosine. Comparison of the antiproliferative IC_50_ values of the compounds alone or in co-treatments with verapamil, a P-GP inhibitor, against HT-29 showed that all the phosphonic acid derivatives are indeed active, but rendered inactive by MDR efflux pumps. The co-treatment with verapamil also renders the hydrogenophosphonate esters with diethylaminopropanol with 5- and 6-carbon-atom spacers active, showing that the original design strategy of combining the naphthalene sulfonamides with APLs leads to active compounds, but they become substrates of the MDR pumps. This MDR sensitivity also explains the low observed selectivity towards cancer cells, as the candidates to provide targeting are not active.

The most potent compounds arrest the cell cycle at 24, 48, and 72 h at G_2_/M, consistent with an antimitotic effect, and increase the SubG_0_/G_1_ population, assigned to apoptotic death. Studies of the cell death mechanism show that the phosphonates induce apoptotic responses in HeLa cells, mostly late, and necrosis, which is usually considered the outcome of apoptosis in vitro. In all cases, the cell death patterns are qualitatively similar to those induced by a typical microtubule inhibitor, such as ABT-751. Immunofluorescent microscopy of HeLa and U-87 M cells shows disruption of microtubule networks and changes in cell shape and volume upon treatment, consistent with a proposed anti-tubulin mechanism of action that disorganizes the cytoskeleton responsible for maintaining cell shape and volume.

Docking experiments provide poses compatible with binding at the colchicine site of tubulin, placing the *N*-trimethoxyphenyl naphthalene sulfonamide within the A and B zones of the colchicine site, respectively, in good agreement with the proposed binding mode for the unconjugated naphthalenesulfonamides. The phosphorus-containing hybrids behave as antimitotic agents, suggesting a minor contribution from the APLs, which are putatively responsible for their becoming substrates of the MDR efflux pumps in a structure-dependent way. The antiproliferative results suggest that uptake is the major limitation to these compounds becoming useful antimitotic agents. The new SMDC constitutes a promising family for further development of novel antitumor compounds, providing useful information on the modifications needed to address these limitations.

## Figures and Tables

**Table 1:** Antiproliferative potency of the compounds against the cancer cell lines HeLa, HT-29, MCF7, and U87 MG, the non-tumorigenic cell line HEK-293, and against HeLa and HT-29 in the presence of verapamil (VP). IC_50_ values are in µM. The mean and confidence intervals are in the Supplementary Material: Supplementary Table S1, ST1. Comp = Compound.

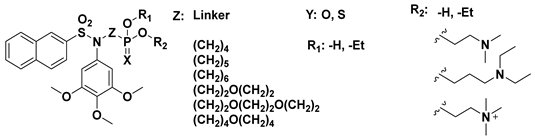													
Comp.	Z	X	R_1_	R_2_	HeLa	HeLa + VP	HT-29	HT-29 + VP	RS	MCF7	U87 MG	HEK 293	SI
**3A**	(CH_2_)_4_	O	Et	Et	1.06	-	1.51	1.11	1.4	1.29	1.16	1.51	1.4
**3B**	(CH_2_)_5_	O	Et	Et	0.26	-	0.34	0.22	1.5	0.28	0.30	0.33	1.3
**3C**	(CH_2_)_2_O(CH_2_)_2_	O	Et	Et	1.24	-	1.54	1.21	1.3	0.86	1.47	1.12	0.9
**3D**	(CH_2_)_6_	O	Et	Et	0.74	-	0.86	0.23	3.7	0.47	1.81	1.93	2.5
**3E**	(CH_2_)_2_[O(CH_2_)_2_]_2_	O	Et	Et	0.66	-	0.74	0.45	1.6	0.90	0.83	0.63	1.0
**3F**	(CH_2_)_4_O(CH_2_)_4_	O	Et	Et	0.64	-	0.54	0.51	1.1	0.51	0.63	0.48	0.8
**4A**	(CH_2_)_4_	O	H	H	3.54	-	6.71	4.38	1.5	5.44	3.12	3.50	1.0
**4B**	(CH_2_)_5_	O	H	H	>10	<10	>10	0.62	>15	-	-	-	-
**4C**	(CH_2_)_2_O(CH_2_)_2_	O	H	H	>10	<10	>10	8.07	>1	-	-	-	-
**4D**	(CH_2_)_6_	O	H	H	>10	<10	>10	10.9	>1	-	-	-	-
**4F**	(CH_2_)_4_O(CH_2_)_4_	O	H	H	5.97	-	>10	>10	-	7.99	18.1	~100	17
**7A**	(CH_2_)_4_	S	Et	Et	4.09	-	>10	>10	-	>10	>10	3.24	0.8
**7B**	(CH_2_)_5_	S	Et	Et	2.53	-	1.94	1.92	1.0	1.21	4.69	1.41	0.6
**7D**	(CH_2_)_6_	S	Et	Et	6.54	-	>10	>10	-	>10	>10	3.83	0.6
**5A**	(CH_2_)_4_	O	H	Et	>10	>10	>10	>10	-	-	-	-	-
**5D**	(CH_2_)_6_	O	H	Et	>10	>10	>10	>10	-	-	-	-	-
**6A-DMAE**	(CH_2_)_4_	O	H	DMAE	>10	>10	>10	>10	-	-	-	-	-
**6C-DMAE**	(CH_2_)_2_O(CH_2_)_2_	O	H	DMAE	>10	>10	>10	>10	-	-	-	-	-
**6F-DMAE**	(CH_2_)_4_O(CH_2_)_4_	O	H	DMAE	>10	>10	>10	>10	-	-	-	-	-
**6A-DEAP**	(CH_2_)_4_	O	H	DEAP	>10	>10	>10	>10	-	-	-	-	-
**6B-DEAP**	(CH_2_)_5_	O	H	DEAP	>10	<10	>10	7.18	>1.5	-	-	-	-
**6D-DEAP**	(CH_2_)_6_	O	H	DEAP	>10	<10	>10	14.3	-	-	-	-	-
**6A-Ch**	(CH_2_)_4_	O	H	Chol	>10	<10	>10	59.7	-	-	-	-	-
**Milt**	-	-	-	-	7.26	-	2.61	1.24	2.1	25.4	0.52	56.2	7.7
**ABT**	-	-	-	-	0.20	-	0.25	0.26	1	0.27	0.31	0.17	0.9

## Data Availability

The data underlying this study are available in the published article and its Supporting Information. Crude data are available from the authors upon request.

## Supplementary Materials

The following supporting information can be downloaded at: https://www.mdpi.com/article/10.3390/pharmaceutics18091064/s1: Supplementary Table S1, ST1. IC_50_ in the different cell lines with confidence intervals. Supplementary Figure S1, SF1. IC_50_ curves in the different cell lines. Supplementary Tables S2–S4, ST2–4. Cell cycle in HeLa, HT-29, and U87 MG, respectively, with the average for each treatment and standard deviation (SD). Supplementary Tables S5–S7, ST5–7. Replicate values for cell cycle in HeLa, HT-29, and U87 MG, respectively. Supplementary Table S8, ST8. Apoptosis assay data, average for each treatment in the three different cell lines and standard deviation (SD). Supplementary Table S9, ST9. Replicate values for the apoptosis assay in the three studied cell lines. Supplementary Figure S2, SF2. Conformations. Supplementary Table S10, ST10. Selected poses. Supplementary Table S11, ST11. Descriptors, physicochemical properties, and pharmacokinetic parameters for the compounds. Supplementary Table S12, ST12. Site of metabolism predictions. Supplementary Figure S3–S38. ^1^H and ^13^C-NMR spectra of the compounds.

## Abbreviations

The following abbreviations are used in this manuscript:

ADC	Antibody Drug Conjugate
AnV	Fluorescein isothiocyanate-labeled Annexin V
APC	Alkyl Phosphocholine
APL	Alkyl Phospholipids
Comp	Compound
DAPI	4′,6-diamidino-2-phenylindole
DMAP	Dimethyl amino pyridine
DMEM	Dulbecco’s modified Eagle’s medium
DMF	Dimethyl formamide
DMSO	Dimethyl sulfoxide
DNA	Desoxyribonucleic acid
EDC	*N*-Ethyl-*N*′-(3-dimethylaminopropyl)carbodiimide hydrochloride
HIFBS	Heat-inactivated fetal bovine serum
HRMS	High Resolution Mass Spectrometry
IC_50_	Inhibitory concentration 50
M.p.	Melting point
MDR	Multidrug resistance
MMFF	Merck Molecular Forcefield
MTT	3-[4,5-dimethylthiazol-2-yl]-2,5-diphenyltetrazolium bromide
NMR	Nuclear Magnetic Resonance
PBS	Phosphate Buffer Saline
PDB	Protein Data Bank
PDC	Peptide Drug Conjugate
P-gp	P-glycoprotein
PI	Propidium iodide
PLE	Phospholipid ethers
RPMI	Roswell Park Memorial Institute
RS	Relative sensitization
SI	Selectivity index
SMDC	Small-molecule drug conjugate
TLC	Thin-layer chromatography
TM	3,4,5-trimethoxyphenyl
TMNS	*N*-3,4,5-Trimethoxyphenyl Naphthalene Sulfonamide
TMS	Trimethyl silyl
TPSA	Topological polar surface area
VP	Verapamil
