# Supplementary material for: Small Molecule Drug Conjugate Hybrids of Naphthalene Sulfonamide and Phospholipid Conjugates Are Microtubule-Disrupting Antitumor Agents
Source: Pharmaceutics. 2026 Aug 26;18(9):1064. doi: 10.3390/pharmaceutics18091064 (PMC13610001; doi:10.3390/pharmaceutics18091064)
Supplement: Supplementary file 1 [file pharmaceutics-18-01064-s001.zip › pharmaceutics-4466471-supplementary.pdf]

Supplementary material

Small molecule drug conjugate hybrids of naphthalene sulfonamide and phospholipid conjugates are antitumor agents acting on tubulin

Noelia Fernández-Ceballos, Laura Gallego-Yerga, Rafael Peláez.

Supp. Table S1, ST1. IC_{50} in µM in the different cell lines and confidence intervals (CI).

Supp. Fig. S1, SF1. IC_{50} curves in the different cell lines.

Suppl. Table S2, ST2. Cell cycle in HeLa, average for each treatment and standard deviation (SD).

Suppl. Table S3, ST3. Cell cycle in HT-29, average for each treatment and standard deviation (SD).

Suppl. Table S4, ST4. Cell cycle in U87 MG, average for each treatment and standard deviation (SD).

Suppl. Table S5, ST5. Replicate values for cell cycle in HeLa.

Suppl. Table S6, ST6. Replicate values for cell cycle in HT-29.

Suppl. Table S7, ST7. Replicate values for cell cycle in U87 MG.

Suppl. Table S8, ST8. Apoptosis assay data, average for each treatment in the three different cell lines and standard deviation (SD).

Suppl. Table S9, ST9. Replicate values for apoptosis assay in the three studied cell lines.

Supp. Fig. S2, SF2. Conformations.

Supp. Table S10, ST10. Selected poses.

Supp. Table S11, ST11. Descriptors, physicochemical properties, and pharmacokinetic parameters for the compounds.

Supp. Table S12, ST12. Site of metabolism predictions.

Supp. Fig. S3–S38. ^{1}H and ^{13}C-NMR spectra of the compounds.

Supp. Table S1. IC_{50} in µM in the different cell lines and confidence intervals (CI).

	HeLa	CI	HT-29	CI	HT-29+VP	CI	MCF7	CI	U87 MG	CI	HEK 293	CI
**3A**	1.06	0.96 to 1.20	1.51	1.28 to 1.76	1.11	0.98 to 1.25	1.29	1.11 to 1.49	1.16	0.74 to 1.58	1.51	1.29 to 1.76
**3B**	0.26	0.22 to 0.32	0.34	0.32 to 0.36	0.22	0.19 to 0.26	0.28	0.24 to 0.33	0.30	0.23 to 0.37	0.33	0.29 to 0.36
**3C**	1.24	1.13 to 1.38	1.54	1.38 to 1.72	1.21	1.04 to 1.44	0.86	0.76 to 0.96	1.47	1.26 to 1.71	1.12	0.97 to 1.35
**3D**	0.74	0.65 to 0.85	0.86	0.76 to 0.95	0.23	0.11 to 0.35	0.47	0.36 to 0.60	1.81	0.99 to 2.68	1.93	0.77 to 3.09
**3E**	0.66	0.59 to 0.73	0.74	0.68 to 0.80	0.45	0.27 to 0.57	0.90	0.80 to 1.03	0.83	0.72 to 0.96	0.63	0.55 to 0.74
**3F**	0.64	0.57 to 0.71	0.54	0.46 to 0.63	0.51	0.37 to 0.65	0.51	0.46 to 0.56	0.63	0.52 to 0.76	0.48	0.41 to 0.57
**4A**	3.54	3.21 to 3.89	6.71	5.86 to 7.56	4.38	2.89 to 5.87	5.44	4.81 to 6.20	3.12	2.26 to 4.09	3.50	2.94 to 4.12
**4B**	>10		>10		0.62	0.43 to 0.80						
**4C**	>10		>10		8.07	6.97 to 9.24						
**4D**	>10		>10		10.9	Wide						
**4F**	5.97	5.54 to 6.46	>10		>10		7.99	7.19 to 8.93	18.1	11.36 to 29.91	~100	Wide
**6B-DEAP**	>10		>10		7.18	6.27 to 8.19						
**6D-DEAP**	>10		>10		14.3	0.61 to 34.71						
**6A-Ch**	>10		>10		59.7	Wide						
**7A**	4.09	3.40 to 4.72	>10		>10		>10		>10		3.24	2.70 to 3.89
**7B**	2.53	2.21 to 2.83	1.94	1.75 to 2.14	1.92	1.54 to 2.34	1.21	0.93 to 1.62	4.69	3.39 to 6.74	1.41	1.13 to 1.69
**7D**	6.54	5.52 to 7.94	>10		>10		>10		>10		3.83	2.91 to 5.15
**Miltefosine**	7.26	6.44 to 8.22	2.61	2.13 to 3.22	1.24	0.59 to 2.52	25.4	2.60 to 48.2	0.52	0.33 to 0.75	56.2	37.7 to 77.5
**ABT**	0.20	0.19 to 0.22	0.25	0.22 to 0.28	0.26	0.23 to 0.31	0.27	0.23 to 0.33	0.31	0.26 to 0.38	0.17	0.15 to 0.18

Supp. Fig. S1. IC_{50} curves in the different cell lines.

IC_{50} curves in HeLa.

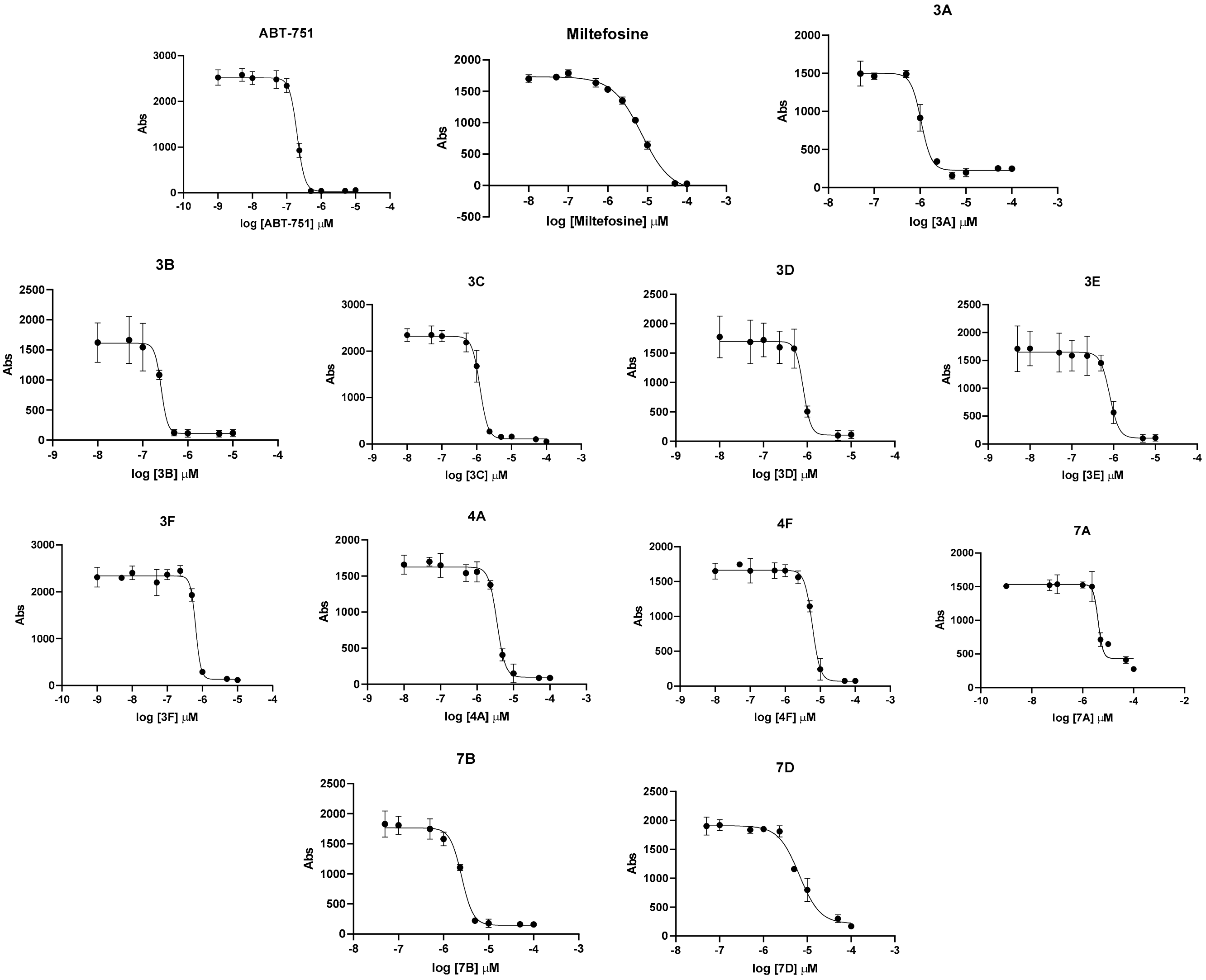

IC_{50} curves in HT-29.

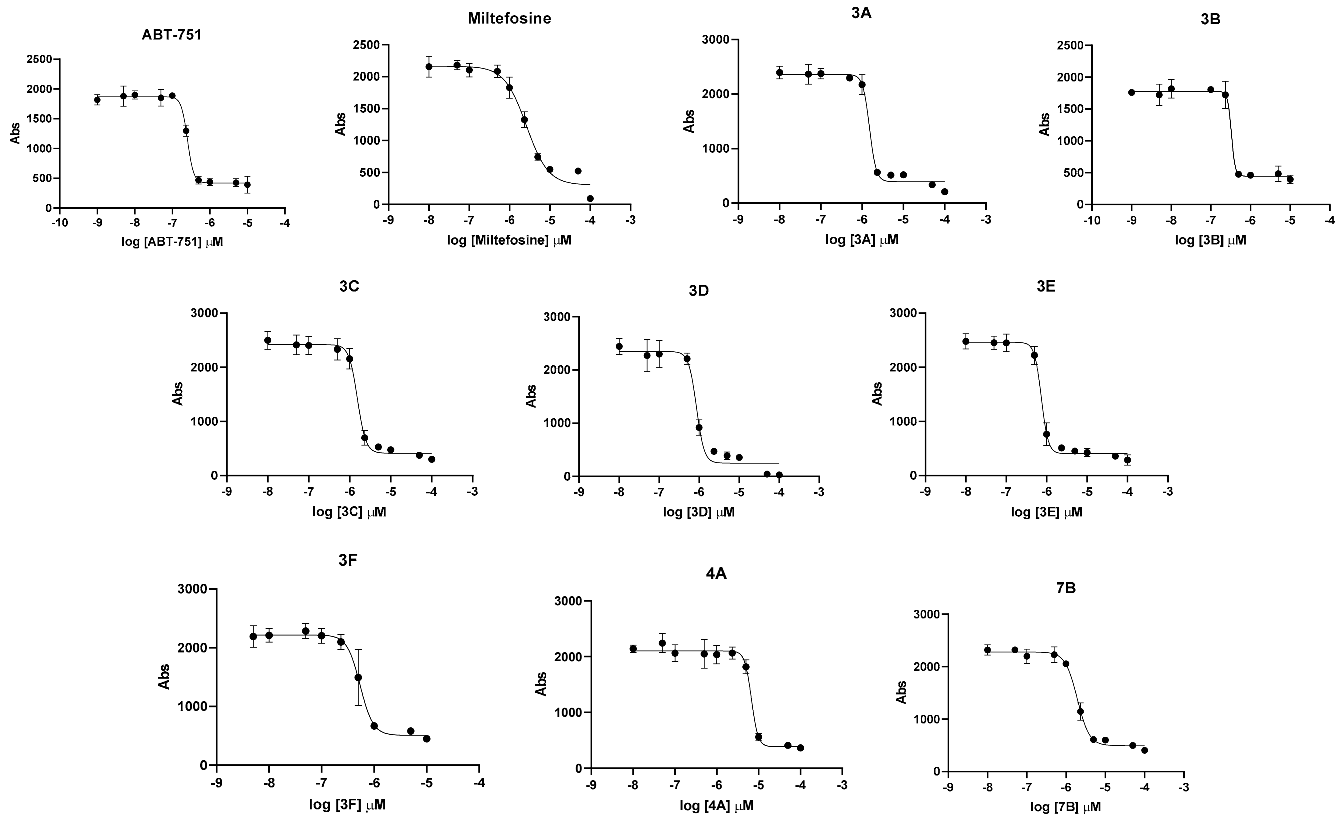

IC_{50} curves in HT-29 + 10 μM Verapamil

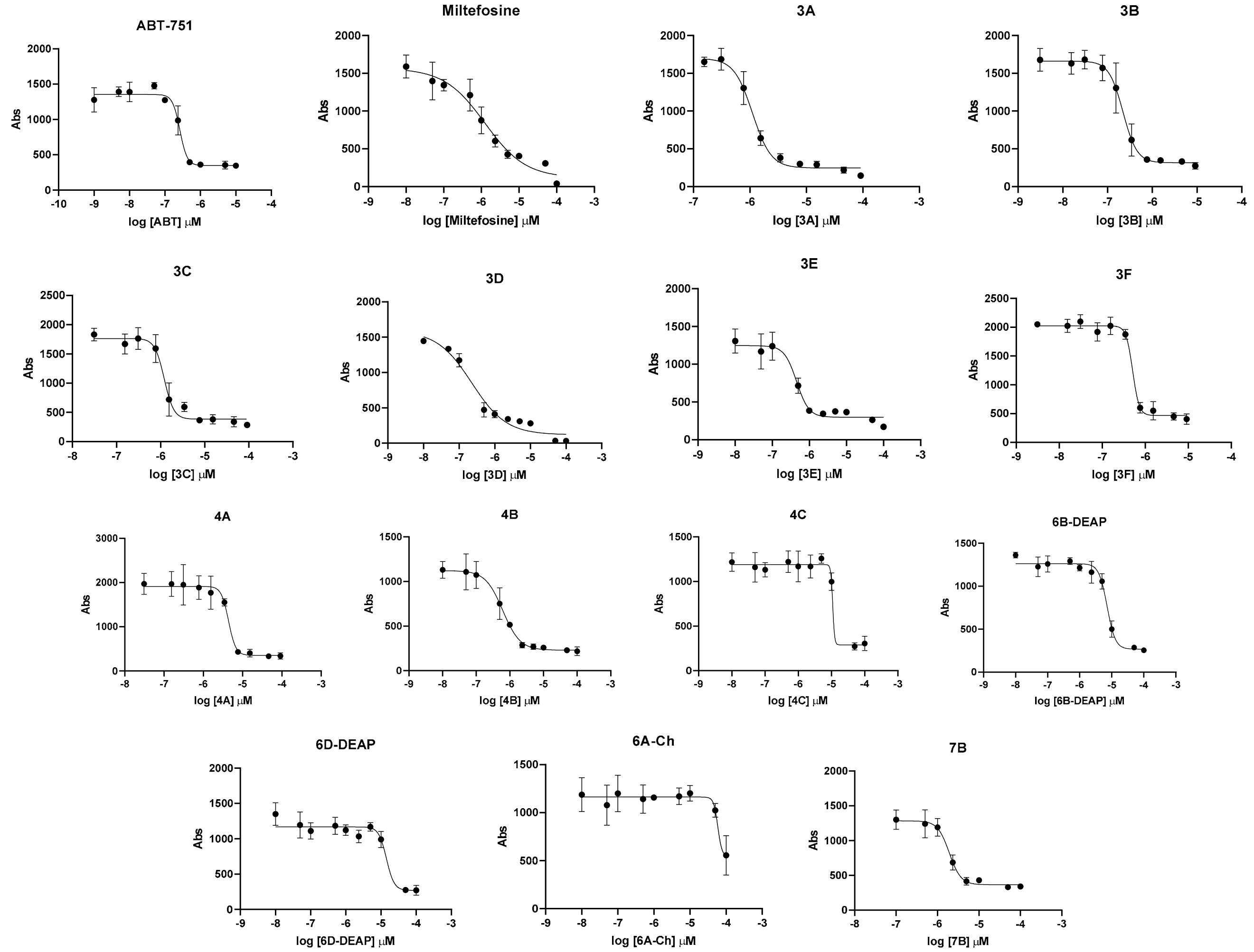

IC$_{50}$ curves in MCF7.

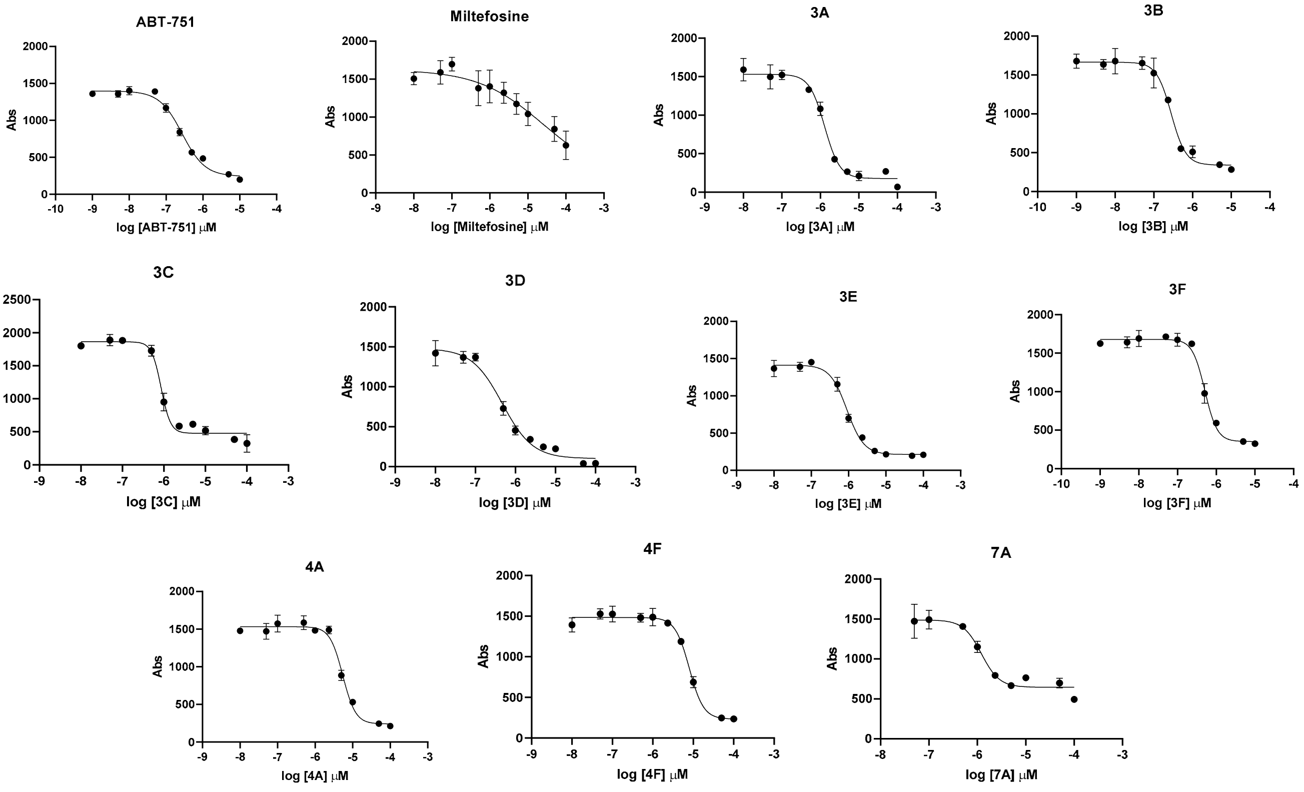

IC$_{50}$ curves in U87 MG.

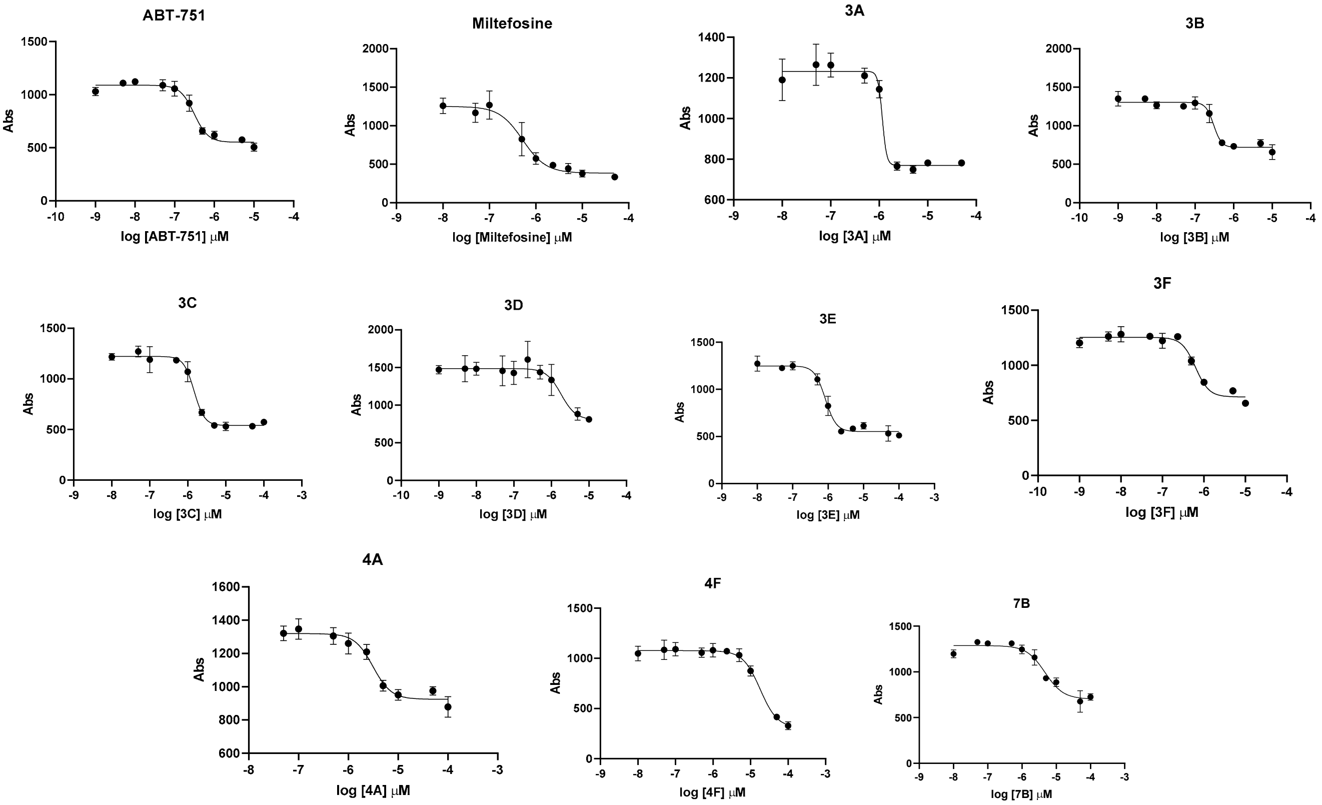

IC$_{50}$ curves in HEK293.

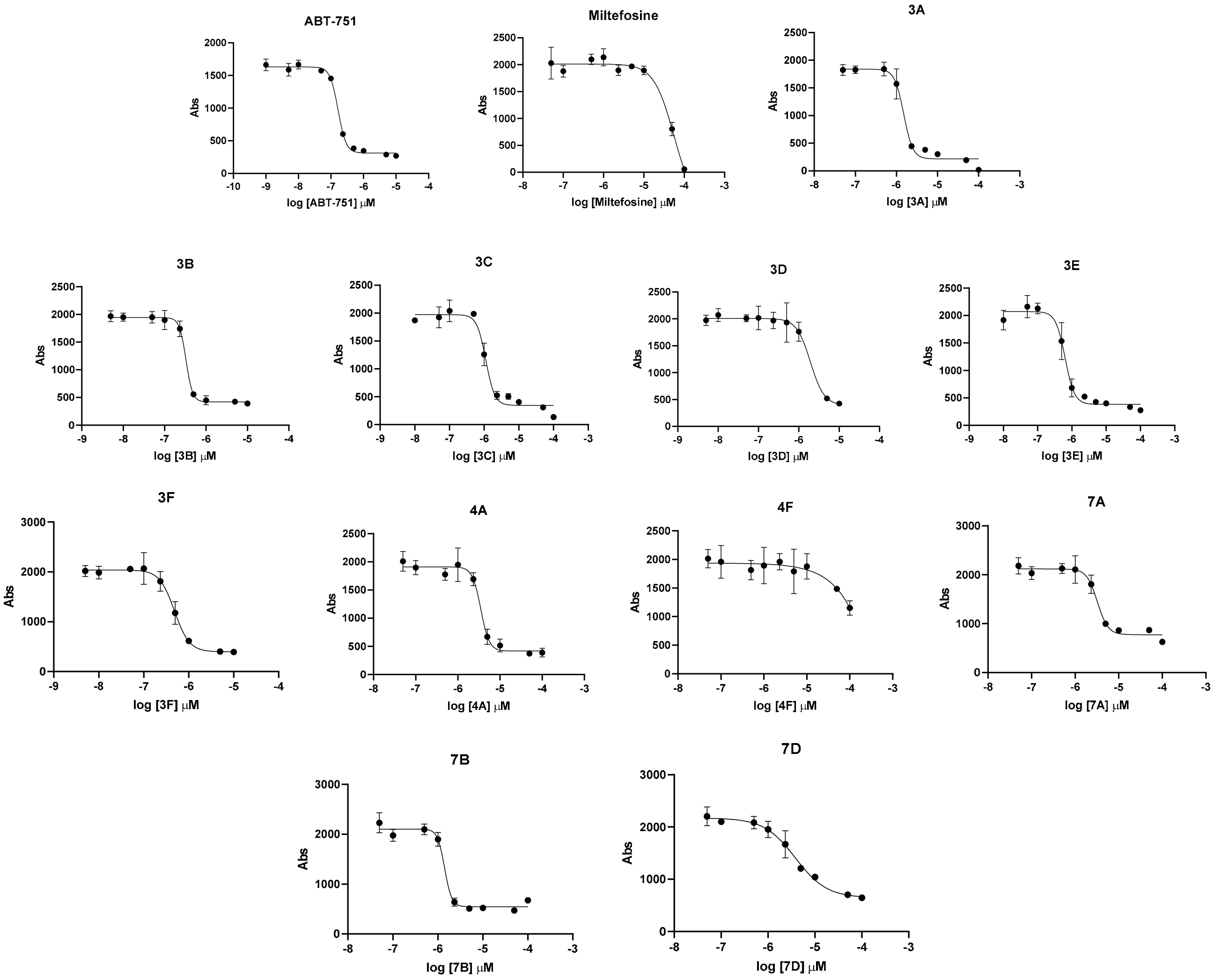

Suppl. Table S2. Cell cycle in HeLa, average for each treatment and standard deviation (SD).

Time after treatment	Cell cycle population	Control (% cells)	SD	ABT (% cells)	SD	3B (% cells)	SD	3F (% cells)	SD
**24 h**	SubG$_0$/G$_1$	4.1	1.4	25.7	3.0	17.0	6.3	12.6	1.8
	G$_0$/G$_1$	78.5	2.7	22.6	2.3	15.0	0.9	25.3	3.0
	S	2.9	0.9	5.9	1.2	8.3	3.6	6.4	0.8
	G$_2$/M	14.8	1.4	45.3	3.1	60.8	3.4	56.1	4.9
**48 h**	SubG$_0$/G$_1$	5.9	3.7	14.1	10.6	27.7	7.4	22.9	4.9
	G$_0$/G$_1$	77.0	3.8	38.4	3.2	17.4	1.0	25.8	1.9
	S	3.1	1.4	11.0	4.1	9.7	3.8	9.0	3.1
	G$_2$/M	14.4	3.7	38.0	8.2	46.3	6.2	43.1	4.4
**72 h**	SubG$_0$/G$_1$	2.8	0.7	50.5	8.4	33.9	5.8	30.6	4.9
	G$_0$/G$_1$	82.2	2.7	25.6	2.5	15.3	2.3	23.7	2.1
	S	3.4	0.8	5.5	2.2	8.2	3.7	7.2	2.5
	G$_2$/M	11.9	2.6	18.5	4.8	43.5	0.9	39.2	3.3

Suppl. Table S3. Cell cycle in HT-29, average for each treatment and standard deviation (SD).

Time after treatment	Cell cycle population	Control (% cells)	SD	ABT (% cells)	SD	3B (% cells)	SD	3F (% cells)	SD
**24 h**	$SubG_0/G_1$	2.7	1.4	40.1	6.1	26.6	6.2	12.3	7.4
	G_0/G_1	61.7	0.8	17.1	5.9	14.4	7.1	37.3	8.1
	S	9.5	5.3	4.1	1.9	5.7	5.6	10.4	4.8
	G_2/M	25.0	5.7	38.2	8.6	52.4	12.1	38.9	5.5
**48 h**	$SubG_0/G_1$	2.0	0.6	40.3	4.6	30.7	3.3	20.2	9.0
	G_0/G_1	71.0	1.7	29.4	16.0	14.6	6.0	24.3	1.3
	S	9.1	2.3	4.3	2.4	6.4	7.0	6.8	2.9
	G_2/M	17.2	4.9	25.7	18.5	47.4	11.7	48.0	10.3
**72 h**	$SubG_0/G_1$	3.0	2.0	54.8	15.0	49.1	12.3	39.1	5.7
	G_0/G_1	73.8	6.6	22.0	7.1	14.5	4.6	20.0	1.1
	S	7.7	4.2	4.9	2.9	6.4	5.7	8.4	4.8
	G_2/M	15.2	2.6	17.7	7.0	29.2	6.9	31.9	10.0

Suppl. Table S4. Cell cycle in U87 MG, average for each treatment and standard deviation (SD).

Time after treatment	Cell cycle population	Control (% cells)	SD	ABT (% cells)	SD	3B (% cells)	SD	3F (% cells)	SD
**24 h**	$SubG_0/G_1$	7.9	1.6	16.8	5.3	37.6	12.1	33.7	2.7
	G_0/G_1	57.5	3.3	39.2	16.9	32.5	10.3	40.5	2.9
	S	4.2	0.3	10.3	9.0	3.2	2.1	3.8	0.5
	G_2/M	30.5	4.0	34.1	29.6	26.9	6.2	22.2	3.0
**48 h**	$SubG_0/G_1$	7.5	1.6	46.8	9.9	39.9	8.3	38.2	3.8
	G_0/G_1	55.5	2.7	22.1	4.7	27.9	7.3	24.8	5.5
	S	3.4	0.3	2.7	0.5	3.8	1.2	4.7	0.3
	G_2/M	33.6	1.6	28.5	14.2	28.7	1.1	32.5	3.7
**72 h**	$SubG_0/G_1$	10.0	3.3	62.7	3.3	62.7	11.6	75.0	2.2
	G_0/G_1	56.2	5.9	21.3	2.2	19.4	4.3	16.8	0.6
	S	4.2	2.2	2.2	0.8	1.9	1.0	1.0	0.2
	G_2/M	29.6	4.8	13.8	4.3	16.0	6.9	7.2	1.4

Suppl. Table S5. Replicate values for cell cycle in HeLa.

Time after treatment	Cell cycle population	Control (% cells)			ABT (% cells)			3B (% cells)			3F (% cells)		
		1	2	3	1	2	3	1	2	3	1	2	3
24 h	SubG$_0$/G$_1$	3.78%	5.62%	2.93%	23.41%	24.59%	29.10%	12.02%	14.80%	24.05%	11.53%	11.56%	14.60%
	G$_0$/G$_1$	81.12%	75.76%	78.67%	23.61%	19.98%	24.30%	15.73%	15.30%	13.92%	23.33%	23.92%	28.73%
	S	2.12%	3.82%	2.63%	6.48%	6.80%	4.50%	10.00%	10.70%	4.21%	7.13%	6.53%	5.60%
	G$_2$/M	13.46%	15.16%	15.80%	45.30%	48.41%	42.20%	64.27%	60.70%	57.46%	59.15%	58.78%	50.40%
48 h	SubG$_0$/G$_1$	10.10%	4.33%	3.17%	10.53%	5.79%	26.03%	24.10%	22.85%	36.26%	19.29%	20.91%	28.40%
	G$_0$/G$_1$	77.22%	73.03%	80.70%	34.96%	41.22%	39.03%	17.21%	16.54%	18.52%	27.16%	23.64%	26.60%
	S	1.80%	4.57%	2.90%	13.00%	13.76%	6.37%	11.26%	12.51%	5.32%	12.00%	9.36%	5.75%
	G$_2$/M	11.44%	18.53%	13.07%	43.86%	41.53%	28.60%	49.30%	50.44%	39.11%	42.89%	47.57%	38.75%
72 h	SubG$_0$/G$_1$	3.10%	1.97%	3.33%	40.80%	54.45%	56.15%	33.16%	28.44%	40.05%	25.50%	31.16%	35.20%
	G$_0$/G$_1$	84.82%	79.33%	82.30%	27.88%	26.03%	22.90%	16.09%	17.06%	12.75%	25.11%	21.23%	24.70%
	S	2.94%	4.33%	3.07%	7.63%	5.48%	3.25%	8.53%	11.68%	4.35%	9.06%	8.25%	4.40%
	G$_2$/M	9.66%	14.80%	11.13%	23.62%	14.04%	17.90%	43.64%	44.44%	42.55%	41.67%	40.42%	35.40%

Suppl. Table S6. Replicate values for cell cycle in HT-29.

Time after treatment	Cell cycle population	Control (% cells)			ABT (% cells)			3B (% cells)			3F (% cells)		
		1	2	3	1	2	3	1	2	3	1	2	3
24 h	SubG$_0$/G$_1$	4.10%	1.37%	2.53%	46.06%	40.38%	33.83%	33.68%	24.10%	22.00%	18.39%	14.42%	3.97%
	G$_0$/G$_1$	60.90%	61.70%	62.47%	20.81%	10.30%	20.23%	22.27%	8.42%	12.53%	30.39%	35.43%	46.20%
	S	3.68%	10.85%	14.07%	4.72%	5.65%	2.07%	4.03%	11.98%	1.23%	12.70%	13.65%	4.83%
	G$_2$/M	31.21%	23.93%	20.00%	28.35%	42.65%	43.73%	39.90%	53.22%	63.97%	38.27%	33.78%	44.73%
48 h	SubG$_0$/G$_1$	1.59%	2.75%	1.80%	34.98%	43.55%	42.37%	33.46%	27.10%	31.60%	30.52%	16.05%	14.13%
	G$_0$/G$_1$	69.10%	71.58%	72.27%	29.06%	45.60%	13.53%	13.43%	21.00%	9.23%	24.25%	25.67%	23.03%
	S	6.53%	9.90%	11.00%	6.64%	4.20%	1.93%	4.06%	14.37%	0.90%	6.89%	9.62%	3.80%
	G$_2$/M	22.76%	13.92%	14.80%	29.26%	5.78%	42.20%	49.00%	35.00%	58.23%	38.29%	46.80%	58.80%
72 h	SubG$_0$/G$_1$	5.25%	2.03%	1.60%	37.60%	61.48%	65.37%	46.30%	38.38%	62.60%	40.35%	32.85%	44.00%
	G$_0$/G$_1$	72.75%	80.90%	67.76%	29.90%	19.78%	16.30%	11.75%	19.72%	11.90%	21.00%	18.88%	20.13%
	S	7.75%	3.47%	11.87%	7.20%	6.00%	1.60%	5.00%	12.68%	1.53%	5.35%	5.98%	14.00%
	G$_2$/M	14.15%	13.30%	18.25%	25.10%	11.30%	16.57%	36.95%	26.75%	23.80%	33.25%	41.25%	21.33%

Suppl. Table S7. Replicate values for cell cycle in U87 MG.

Time after treatment	Cell cycle population	Control (% cells)			ABT (% cells)			3B (% cells)			3F (% cells)		
		1	2	3	1	2	3	1	2	3	1	2	3
24 h	SubG_0/G_1	6.47%	7.70%	9.57%	10.85%	18.97%	20.70%	24.17%	40.97%	47.73%	35.70%	34.73%	30.63%
	G_0/G_1	55.10%	61.23%	56.03%	21.05%	42.20%	54.43%	39.70%	37.13%	20.70%	41.80%	37.17%	42.50%
	S	3.83%	4.27%	4.50%	5.05%	5.17%	20.70%	5.53%	2.50%	1.60%	3.57%	3.50%	4.40%
	G_2/M	34.70%	26.73%	29.93%	63.75%	33.83%	4.57%	30.77%	19.73%	30.13%	18.97%	24.80%	22.70%
48 h	SubG_0/G_1	6.43%	6.67%	9.33%	33.47%	54.70%	52.20%	34.10%	36.20%	49.37%	39.97%	40.80%	33.87%
	G_0/G_1	56.03%	57.87%	52.63%	35.50%	15.47%	15.23%	33.63%	30.30%	19.63%	26.70%	18.60%	28.97%
	S	3.37%	3.73%	3.07%	3.57%	1.93%	2.53%	4.87%	3.93%	2.47%	4.47%	4.50%	5.00%
	G_2/M	34.17%	31.83%	34.93%	27.60%	27.87%	30.10%	27.57%	29.73%	28.73%	28.90%	36.27%	32.20%
72 h	SubG_0/G_1	11.23%	12.50%	6.20%	66.17%	62.30%	59.67%	72.47%	65.70%	49.83%	76.90%	72.53%	75.47%
	G_0/G_1	51.60%	54.13%	62.80%	21.93%	23.17%	18.93%	17.40%	16.50%	24.37%	16.33%	17.50%	16.57%
	S	3.13%	2.80%	6.80%	1.87%	1.67%	3.10%	0.93%	1.83%	2.90%	0.83%	1.17%	0.97%
	G_2/M	33.83%	30.47%	24.37%	10.03%	12.83%	18.47%	9.10%	16.03%	22.93%	5.90%	8.77%	7.00%

Suppl. Table S8. Apoptosis assay data, average for each treatment in the three different cell lines and standard deviation (SD).

HeLa	Control – (% cells)	SD	ABT (% cells)	SD	3B (% cells)	SD	3F (% cells)	SD
Live	91.1	0.7	83.5	1.8	45.9	0.8	63.4	3.6
Early apoptosis	0.2	0.1	0.4	0.1	7.3	0.2	4.5	0.8
Late apoptosis	0.2	0.1	0.6	0.3	16.3	1.1	7.2	1.1
Necrosis	8.5	0.8	15.5	1.5	30.5	1.2	24.9	2.9

HT-29	Control – (% cells)	SD	ABT (% cells)	SD	3B (% cells)	SD	3F (% cells)	SD
Live	84.0	4.5	74.3	4.0	52.6	0.9	60.0	0.4
Early apoptosis	0.1	0.0	6.9	2.8	19.9	1.3	15.7	0.4
Late apoptosis	2.9	0.8	13.0	1.7	22.3	0.9	18.9	0.5
Necrosis	13.1	3.8	5.9	0.9	5.2	1.0	5.5	0.4

U87 MG	Control – (% cells)	SD	ABT (% cells)	SD	3B (% cells)	SD	3F (% cells)	SD
Live	89.0	1.6	58.2	1.8	73.0	2.3	70.2	2.5
Early apoptosis	0.7	0.2	9.5	2.0	11.9	1.3	11.5	0.7
Late apoptosis	2.3	0.5	18.9	1.6	10.9	0.7	13.1	1.5
Necrosis	8.0	2.2	13.4	1.8	4.3	0.4	5.2	0.4

Suppl. Table S9. Replicate values for apoptosis assay in the three studied cell lines.

Cell line	Cell viability state	Control (% cells)			ABT (% cells)			3B (% cells)			3F (% cells)		
		1	2	3	1	2	3	1	2	3	1	2	3
HeLa	Live	91.48%	91.52%	90.29%	83.01%	82.03%	85.56%	46.80%	45.67%	45.17%	67.48%	61.10%	61.57%
	Early apoptosis	0.16%	0.29%	0.06%	0.42%	0.38%	0.32%	7.13%	7.23%	7.60%	4.61%	5.20%	3.67%
	Late apoptosis	0.32%	0.16%	0.25%	0.82%	0.81%	0.22%	16.20%	15.17%	17.40%	6.09%	8.30%	7.30%
	Necrosis	8.04%	8.04%	9.40%	15.75%	16.77%	13.89%	29.87%	31.93%	29.83%	21.82%	25.40%	27.47%
HT-29	Live	89.11%	81.87%	80.93%	70.17%	78.23%	74.50%	53.00%	53.17%	51.60%	60.17%	60.33%	59.50%
	Early apoptosis	0.13%	0.07%	0.07%	9.63%	4.13%	6.80%	21.07%	18.57%	20.17%	15.57%	16.13%	15.37%
	Late apoptosis	2.04%	3.53%	3.03%	14.20%	11.00%	13.77%	21.57%	21.97%	23.30%	19.07%	18.27%	19.23%
	Necrosis	8.72%	14.53%	15.97%	6.00%	6.63%	4.93%	4.37%	6.30%	4.93%	5.20%	5.27%	5.90%
U87 MG	Live	89.39%	90.37%	87.20%	57.47%	56.90%	60.32%	75.65%	72.15%	71.30%	67.40%	71.50%	71.80%
	Early apoptosis	0.93%	0.77%	0.50%	10.71%	10.65%	7.27%	10.40%	12.60%	12.60%	12.25%	11.10%	11.05%
	Late apoptosis	2.36%	2.70%	1.80%	20.04%	19.55%	17.02%	10.15%	10.80%	11.60%	14.75%	12.50%	11.95%
	Necrosis	7.32%	6.17%	10.50%	11.79%	12.90%	15.39%	3.80%	4.45%	4.50%	5.60%	4.90%	5.20%

Suppl. Fig. S2. Conformations. Superpositions of the conformations of lower energy adopting a (+)-gauche disposition of the sulfonamide bond (similar to that required for binding to the A and B zones of the colchicine site) for the ligands **3B**, **3C**, **4B** and **4C**.

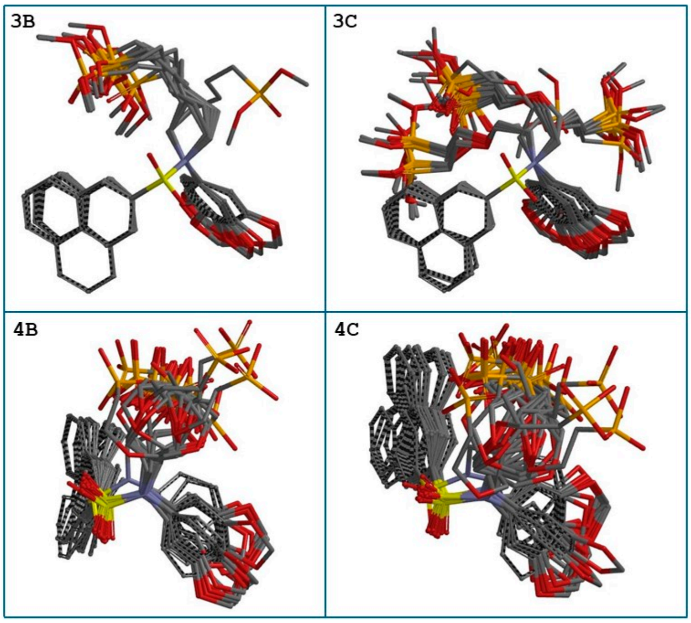

Suppl. Table S10. Selected poses. The PDB ID for that pose, the zones that the ligand of PDB occupies and the zones occupied by the pose, the PLANTS and AD4 scores, and the derived 0-1 scores and Z-scores.

Comp (poses)	Protein	Site Zones	Occ. Zones	0-1 score	Z score	PLANTS score	AD4 score
**1**							
5JVD_28	5JVD	AC	AB	1,0	2,9		-10,5
6AGK_conf_01	6AGK	BC	AB	0,8	2,6	-91,3	
**3A**							
5XAF_14	5XAF	BC	AB	1,0	1,6		-12,3
5XIW_conf_01	5XIW	BC	AB	1,0	1,8	-104,6	
**3B**							
5XAG_70	5XAG	AB	AB	1,0	1,4		-11,9
5GON_bLact_conf_01	bLact	BC	AB	1,0	1,8	-104,3	
**3C**							
5XAG_59	5XAG	AB	AB	1,0	1,4		-11,3
5XAF_conf_01	5XAF	BC	AB	1,0	1,8	-101,6	
**3D**							
7CPD_17	7CPD	AB	AB	1,0	1,2		-10,7
6GJ4_conf_01	6GJ4	BC	AB	1,0	1,9	-110,7	
**3E**							
6GJ4_conf_01	6GJ4	BC	AB	1,0	1,8	-109,1	
5YLS_70	5YLS	BC	ABC	1,0	1,2		-10,0
6PC4wn_97	6PC4wn	AB	AB	1,0	1,2		-10,1
5XLT_conf_01	5XLT	BC	AB	1,0	1,6	-105,0	
**3F**							
7CQP_44	7CQP	BC	AB	1,0	1,2		-11,1
7CQP_conf_02	7CQP	BC	AB	1,0	1,7	-110,9	

**4A**							
5XLT_conf_01	5XLT	BC	AB	1,0	2,8	-116,0	
5XAG_22	5XAG	AB	AB	1,0	1,7		-9,1
**4B**							
5Z4P_conf_01	5Z4P	BC	AB	1,0	3,2	-128,1	
TM_6AGK_41	6AGK	BC	AB	1,0	1,6		-9,8
**4C**							
8C0F_conf_01	8C0F	BC	AB	1,0	2,7	-117,6	
8C0F_68	8C0F	BC	AB	1,0	1,5		-8,1
**4D**							
bLact_conf_01	bLact	BC	AB	1,0	2,8	-122,6	
6AGK_39	6AGK	BC	AB	1,0	1,6		-9,4
**4E**							
6XESw_conf_01	6XESw	BC	AB	1,0	2,7	-123,242	
7LZ8_68	7LZ8	BC	AB	1,0	1,4		-8,7
**4F**							
6XET_54	6XET	AB	AB	1,0	1,3		-8,8
5GON_bLact_conf_01	bLact	BC	AB	1,0	2,3	-121,2	
**5A**							
6ZWC_conf_01	6ZWC	AB	AB	1,0	2,3	-107,5	
TM_6ZWC_15	6ZWC	AB	AB	1,0	1,7		-10,6
**5B**							
5GON_bLact_39	bLact	BC	AB	1,0	1,4		-9,2
5XLT_conf_02	5XLT	BC	AB	0,9	2,1	-106,6	
**5C**							
5GON_bLact_conf_01	bLact	BC	AB	1,0	2,3	-111,4	
5XAG_31	5XAG	AB	AB	1,0	1,5		-9,4
**5D**							
8C0F_44	8C0F	BC	AB	1,0	1,3		-9,6
5XLT_conf_01	5XLT	BC	AB	0,9	1,7	-105,7	
**5E**							
7CPD_conf_01	7CPD	AB	ABC	1,0	2,3	-114,3	
5YLS_24	5YLS	BC	ABC	1,0	1,5		-10,4
6AGK_97	6AGK	BC	AB	1,0	1,3		-9,2
6AGK_conf_01	6AGK	BC	AB	1,0	1,9	-107,1	
**7A**							
5XAF_40	5XAF	BC	AB	1,0	1,3		-11,4
5YLJ_conf_01	5YLJ	BC	ABC	0,9	1,6	-99,0	
**7B**							
7CQP_95	7CQP	BC	AB	1,0	1,2		-10,9
6GJ4_conf_01	6GJ4	BC	AB	1,0	1,9	-106,7	
**7C**							
5Z4U_conf_01	5Z4U	BC	AB	1,0	1,9	-108,0	
5XAF_9	5XAF	BC	AB	1,0	1,4		-11,5
**7D**							
6XES_78	6XES	BC	AB	1,0	1,3		-11,9
6AGK_conf_01	6AGK	BC	AB	1,0	1,8	-105,9	

**7E**							
6AGK_28	6AGK	BC	AB	1,0	1,2		-10,8
6GJ4_conf_01	6GJ4	BC	AB	1,0	1,7	-104,4	
**7F**							
5YLS_17	5YLS	BC	ABC	1,0	1,2		-11,5
7CQP_conf_01	7CQP	BC	ABC	1,0	1,9	-110,8	
**6A-Chol**							
6GJ4_conf_01	6GJ4	BC	AB	1,0	1,9	-104,5	
6AGK_56	6AGK	BC	AB	1,0	1,4		-11,4
**6B-Chol**							
5XLT_53	5XLT	BC	AB	1,0	1,3		-10,5
5XLT_conf_01	5XLT	BC	AB	0,9	1,7	-102,6	
**6C-Chol**							
6GJ4_conf_01	6GJ4	BC	AB	1,0	1,8	-103,4	
6Y6D_35	6Y6D	BC	AB	1,0	1,2		-9,2
**6D-Chol**							
6GJ4_conf_01	6GJ4	BC	AB	1,0	1,8	-111,1	
7DBD_96	7DBD	BC	AB	1,0	1,2		-9,4
**6A-DMEA**							
5XAF_75	5XAF	BC	AB	1,0	1,6		-12,0
5XLT_conf_01	5XLT	BC	AB	1,0	1,8	-105,1	
**6B-DMEA**							
6AGK_87	6AGK	BC	AB	1,0	1,5		-11,8
6AGK_conf_01	6AGK	BC	AB	0,9	1,7	-100,0	
**6C-DMEA**							
5Z4U_conf_01	5Z4U	BC	AB	1,0	1,8	-103,7	
5XLT_54	5XLT	BC	AB	1,0	1,3		-9,9
**6D-DMEA**							
6HX8_conf_01	6HX8	BC	AB	1,0	2,2	-115,7	
7DBB_44	7DBB	BC	AB	1,0	1,4		-11,6
**6F-DMEA**							
7CPDw_22	7CPDw	AB	ABC	1,0	1,2		-10,0
7CQP_conf_02	7CQP	BC	AB	1,0	1,8	-111,6	
**6A-DEAP**							
5XLT_conf_01	5XLT	BC	AB	1,0	1,8	-108,9	
6ZWC_45	6ZWC	AB	AB	1,0	1,3		-11,7
**6B-DEAP**							
7CQP_10	7CQP	BC	AB	1,0	1,2		-10,4
TUB_M1H_conf_01	M1H	BC	AB	1,0	1,6	-104,7	
**6C-DEAP**							
6GJ4_conf_01	6GJ4	BC	ABC	1,0	1,8	-112,7	
6BR1_46	6BR1	BC	AB	1,0	1,2		-11,1
**6D-DEAP**							
3HKC_64	3HKC	BC	AB	0,9	0,8		-5,7
3HKC_conf_07	3HKC	BC	AB	0,7	0,2	-58,8	

Supp. Table S11. Descriptors, physicochemical properties, and pharmacokinetic parameters for the compounds.

**Compound**	Formula	MW	#eavy at	#Arha	FCsp3	#Rot bonds	#HBA	#HBD	MR	TPSA	XLOGP3	WLOGP	MLOGP	cos-IT Lo	sensus L
**3A**	C27H36NO7PS2	581,68	38	16	0,41	15	7	0	156,83	133,81	6,21	7,96	2,52	6	5,67
**3B**	C28H38NO8PS	579,64	39	16	0,43	16	8	0	154,73	118,79	4,84	7,58	2,75	4,22	4,85
**3D**	C27H36NO9PS	581,61	39	16	0,41	16	9	0	151,01	128,02	3,62	6,42	1,79	3,36	3,8
**3C**	C29H40NO8PS	593,67	40	16	0,45	17	8	0	159,54	118,79	5,2	7,97	2,95	4,63	5,19
**3E**	C29H40NO10PS	625,67	42	16	0,45	19	10	0	161,71	137,25	3,48	6,44	1,42	3,75	3,77
**3F**	C31H44NO9PS	637,72	43	16	0,48	20	9	0	170,23	128,02	5,05	7,98	2,56	5,03	5,16
**4A**	C23H26NO8PS--	507,49	34	16	0,3	11	8	0	127,78	146,45	2,68	5,98	1,73	1,85	3,06
**4B**	C24H28NO8PS--	521,52	35	16	0,33	12	8	0	132,59	100,33	3,04	6,37	1,94	2,26	3,4
**4C**	C23H26NO9PS--	523,49	35	16	0,3	12	9	0	128,87	109,56	1,82	5,21	0,96	1,41	2,35
**4D**	C25H30NO8PS--	535,55	36	16	0,36	13	8	0	137,4	100,33	3,39	6,76	2,15	2,67	3,74
**4E**	C25H30NO10PS--	567,55	38	16	0,36	15	10	0	139,57	164,91	1,67	5,23	0,62	1,79	2,33
**4F**	C27H34NO9PS--	579,6	39	16	0,41	16	9	0	148,1	155,68	3,25	6,77	1,79	3,06	3,72
**5A**	C25H31NO8PS-	536,55	36	16	0,36	13	8	0	138,85	132,62	3,58	6,58	2,15	2,82	3,78
**5B**	C26H33NO8PS-	550,58	37	16	0,38	14	8	0	143,66	132,62	3,94	6,97	2,35	3,24	4,13
**5C**	C25H31NO9PS-	552,55	37	16	0,36	14	9	0	139,94	141,85	2,72	5,82	1,38	2,38	3,08
**5D**	C27H35NO8PS-	564,61	38	16	0,41	15	8	0	148,47	132,62	4,3	7,36	2,56	3,65	4,47
**5E**	C27H35NO10PS-	596,61	40	16	0,41	17	10	0	150,64	151,08	2,57	5,84	1,02	2,77	3,05
**5F**	C29H39NO9PS-	608,66	41	16	0,45	18	9	0	159,16	141,85	4,15	7,38	2,18	4,04	4,44
**6A-Chol**	C28H39N2O8PS	594,66	40	16	0,43	15	8	0	155,65	132,62	3,27	6,27	-1,7	0,11	1,99
**6A-DMAE**	C27H36N2O8PS-	579,62	39	16	0,41	15	9	0	151,36	135,86	0,97	6,12	1,79	2,45	2,83
**6A-DMAEH**	C27H36N2O8PS-	579,62	39	16	0,41	15	9	0	151,36	135,86	0,97	6,12	1,79	2,45	2,83
**6A-DEAP**	C30H42N2O8PS-	621,7	42	16	0,47	18	9	0	165,78	135,86	2,06	7,29	2,37	3,7	3,86
**6A-DEAPH**	C30H43N2O8PS	622,71	42	16	0,47	18	8	1	167,04	137,06	2,06	5,88	-1,31	3,8	2,61
**6A-Pip**	C29H39N2O8PS	606,67	41	16	0,45	13	8	1	163,74	137,06	1,53	4,86	-1,9	2,68	1,79
**6B-DEAP**	C31H44N2O8PS-	635,73	43	16	0,48	19	9	0	170,59	135,86	2,42	7,68	2,56	4,12	4,2
**6B-DEAPH**	C31H45N2O8PS	636,74	43	16	0,48	19	8	1	171,85	137,06	2,42	6,27	-1,12	4,22	2,95
**6C-DMAE**	C27H36N2O9PS-	595,62	40	16	0,41	16	10	0	152,45	145,09	0,11	5,36	1,02	2,01	2,13
**6C-DMAEH**	C27H37N2O9PS	596,63	40	16	0,41	16	9	1	153,7	146,29	0,11	3,94	-2,66	2,1	0,87
**6D-DEAP**	C32H46N2O8PS-	649,76	44	16	0,5	20	9	0	175,4	135,86	2,78	8,07	2,75	4,54	4,54
**6D-DEAEH**	C32H47N2O8PS	650,76	44	16	0,5	20	8	1	176,65	137,06	2,78	6,66	-0,93	4,64	3,29
**6F-DMAE**	C31H44N2O9PS-	651,73	44	16	0,48	20	10	0	171,67	145,09	1,54	6,92	1,8	3,68	3,49
**6F-DMAEH**	C31H45N2O9PS	652,74	44	16	0,48	20	9	1	172,93	146,29	1,54	5,5	-1,88	3,78	2,23

Cont.

**Compound**	ESOL Log S	olubility (r	Solubility (m	ESOL Class	Ali Log S	olubility (mg	olubility (m	Ali Class	icos-IT Log	T Solubility (	-IT Solubility (	cos-IT cla
**3A**	-6,68	1,21E-04	2,09E-07	oorly solub	-8,81	9,11E-07	1,57E-09	Poorly	-8,86	7,99E-07	1,37E-09	Poorly
**3B**	-5,73	1,08E-03	1,86E-06	lerately sol	-7,07	4,95E-05	8,55E-08	Poorly	-9,07	4,98E-07	8,59E-10	Poorly
**3D**	-4,97	6,17E-03	1,06E-05	lerately sol	-6	5,87E-04	1,01E-06	Moderately	-8,4	2,32E-06	3,99E-09	Poorly
**3C**	-5,97	6,35E-04	1,07E-06	lerately sol	-7,44	2,15E-05	3,62E-08	Poorly	-9,45	2,10E-07	3,53E-10	Poorly
**3E**	-4,94	7,19E-03	1,15E-05	lerately sol	-6,04	5,65E-04	9,02E-07	Poorly	-8,89	8,06E-07	1,29E-09	Poorly
**3F**	-5,93	7,48E-04	1,17E-06	lerately sol	-7,48	2,11E-05	3,31E-08	Poorly	-9,94	7,29E-08	1,14E-10	Poorly
**4A**	-4,3	2,56E-02	5,05E-05	lerately sol	-5,41	1,98E-03	3,91E-06	Moderately	-6,53	1,49E-04	2,93E-07	Poorly
**4B**	-4,53	1,52E-02	2,92E-05	lerately sol	-4,81	8,03E-03	1,54E-05	Moderately	-6,92	6,26E-05	1,20E-07	Poorly
**4C**	-3,78	8,72E-02	1,67E-04	Soluble	-3,74	9,51E-02	1,82E-04	Soluble	-6,25	2,92E-04	5,57E-07	Poorly
**4D**	-4,77	9,16E-03	1,71E-05	lerately sol	-5,18	3,57E-03	6,67E-06	Moderately	-7,31	2,63E-05	4,91E-08	Poorly
**4E**	-3,73	1,05E-01	1,85E-04	Soluble	-4,75	1,02E-02	1,79E-05	Moderately	-6,75	1,01E-04	1,78E-07	Poorly
**4F**	-4,73	1,08E-02	1,87E-05	lerately sol	-6,19	3,72E-04	6,41E-07	Poorly	-7,8	9,10E-06	1,57E-08	Poorly
**5A**	-4,89	6,87E-03	1,28E-05	lerately sol	-6,05	4,77E-04	8,89E-07	Poorly	-7,61	1,33E-05	2,47E-08	Poorly
**5B**	-5,13	4,06E-03	7,38E-06	lerately sol	-6,42	2,07E-04	3,76E-07	Poorly	-7,99	5,58E-06	1,01E-08	Poorly
**5C**	-4,38	2,33E-02	4,21E-05	lerately sol	-5,35	2,45E-03	4,44E-06	Moderately	-7,33	2,60E-05	4,70E-08	Poorly
**5D**	-5,37	2,40E-03	4,25E-06	lerately sol	-6,8	8,98E-05	1,59E-07	Poorly	-8,38	2,35E-06	4,16E-09	Poorly
**5E**	-4,33	2,78E-02	4,66E-05	lerately sol	-5,39	2,43E-03	4,07E-06	Moderately	-7,82	9,01E-06	1,51E-08	Poorly
**5F**	-5,33	2,85E-03	4,69E-06	lerately sol	-6,84	8,87E-05	1,46E-07	Poorly	-8,87	8,14E-07	1,34E-09	Poorly
**6A-Chol**	-4,89	7,61E-03	1,28E-05	lerately sol	-5,73	1,11E-03	1,86E-06	Moderately	-8,77	1,02E-06	1,71E-09	Poorly
**6A-DMAE**	-3,36	2,54E-01	4,38E-04	Soluble	-3,41	2,25E-01	3,88E-04	Soluble	-7,68	1,21E-05	2,08E-08	Poorly
**6A-DMAEH**	-3,36	2,54E-01	4,38E-04	Soluble	-3,41	2,25E-01	3,88E-04	Soluble	-7,68	1,21E-05	2,08E-08	Poorly
**6A-DEAP**	-4,09	5,10E-02	8,20E-05	lerately sol	-4,54	1,79E-02	2,87E-05	Moderately	-8,84	9,01E-07	1,45E-09	Poorly
**6A-DEAPH**	-4,09	5,03E-02	8,08E-05	lerately sol	-4,57	1,69E-02	2,71E-05	Moderately	-9,56	1,73E-07	2,77E-10	Poorly
**6A-Pip**	-4	6,12E-02	1,01E-04	Soluble	-4,02	5,83E-02	9,61E-05	Moderately	-8,32	2,87E-06	4,73E-09	Poorly
**6B-DEAP**	-4,33	2,99E-02	4,70E-05	lerately sol	-4,92	7,72E-03	1,21E-05	Moderately	-9,22	3,80E-07	5,97E-10	Poorly
**6B-DEAPH**	-4,33	2,95E-02	4,64E-05	lerately sol	-4,94	7,30E-03	1,15E-05	Moderately	-9,94	7,28E-08	1,14E-10	Poorly
**6C-DMAE**	-2,84	8,57E-01	1,44E-03	Soluble	-2,71	1,16E+00	1,94E-03	Soluble	-7,4	2,37E-05	3,97E-08	Poorly
**6C-DMAEH**	-2,85	8,46E-01	1,42E-03	Soluble	-2,74	1,09E+00	1,83E-03	Soluble	-8,12	4,55E-06	7,62E-09	Poorly
**6D-DEAP**	-4,57	1,75E-02	2,70E-05	lerately sol	-5,29	3,34E-03	5,14E-06	Moderately	-9,61	1,60E-07	2,46E-10	Poorly
**6D-DEAEH**	-4,58	1,73E-02	2,66E-05	lerately sol	-5,31	3,16E-03	4,85E-06	Moderately	-10,33	3,06E-08	4,71E-11	Insoluble
**6F-DMAE**	-3,8	1,03E-01	1,58E-04	Soluble	-4,2	4,15E-02	6,37E-05	Moderately	-8,94	7,45E-07	1,14E-09	Poorly
**6F-DMAEH**	-3,81	1,02E-01	1,56E-04	Soluble	-4,22	3,92E-02	6,01E-05	Moderately	-9,66	1,43E-07	2,19E-10	Poorly

Cont.

Compound	I absorptio	B permea	substr	g Kp (cm/ski	#viola	se #violati	er #violati	n #violati	gge #viola	vailability	SNS #al	enk #ale	eness #vi	etic Access
**3A**	Low	No	Yes	-5,44	1	4	1	2	1	0,55	0	1	3	4,59
**3B**	Low	No	Yes	-6,4	1	4	1	1	1	0,55	0	1	3	4,64
**3D**	Low	No	Yes	-7,28	1	4	1	1	1	0,55	0	1	3	4,72
**3C**	Low	No	Yes	-6,23	1	4	1	1	2	0,55	0	1	3	4,78
**3E**	Low	No	Yes	-7,65	2	4	1	2	2	0,17	0	1	2	5,07
**3F**	Low	No	Yes	-6,6	1	4	1	1	3	0,55	0	1	3	5,13
**4A**	Low	No	Yes	-7,49	1	2	2	2	0	0,56	0	1	2	3,84
**4B**	Low	No	Yes	-7,32	1	3	1	1	0	0,56	0	1	2	4,36
**4C**	High	No	Yes	-8,2	1	1	1	0	0	0,56	0	1	2	4,4
**4D**	Low	No	Yes	-7,16	1	3	1	1	0	0,56	0	1	2	4,5
**4E**	Low	No	Yes	-8,58	2	2	2	1	1	0,11	0	1	2	4,3
**4F**	Low	No	Yes	-7,53	1	4	2	2	2	0,11	0	1	2	4,38
**5A**	Low	No	Yes	-7,03	1	3	1	2	0	0,56	0	1	3	4,7
**5B**	Low	No	Yes	-6,86	1	3	1	2	0	0,56	0	1	3	4,76
**5C**	Low	No	Yes	-7,74	1	3	2	1	0	0,56	0	1	2	4,85
**5D**	Low	No	Yes	-6,69	1	4	1	2	0	0,56	0	1	3	4,91
**5E**	Low	No	Yes	-8,11	2	4	2	1	2	0,11	0	1	2	5,2
**5F**	Low	No	Yes	-7,07	1	4	2	2	2	0,56	0	1	3	5,25
**6A-Chol**	Low	No	Yes	-7,61	1	4	1	2	0	0,55	0	2	2	5,26
**6A-DMAE**	Low	No	Yes	-9,15	1	4	1	2	0	0,55	0	1	2	5,1
**6A-DMAEH**	Low	No	Yes	-9,15	1	4	1	2	0	0,55	0	1	2	5,1
**6A-DEAP**	Low	No	Yes	-8,63	1	4	1	2	2	0,55	0	1	2	5,45
**6A-DEAPH**	Low	No	Yes	-8,64	1	4	1	1	2	0,55	0	1	2	5,49
**6A-Pip**	Low	No	Yes	-8,91	1	3	1	1	1	0,55	0	1	2	5,7
**6B-DEAP**	Low	No	Yes	-8,46	1	4	1	2	2	0,55	0	1	2	5,52
**6B-DEAPH**	Low	No	Yes	-8,47	1	4	1	2	2	0,55	0	1	2	5,56
**6C-DMAE**	Low	No	Yes	-9,86	2	3	2	1	1	0,17	0	1	2	5,25
**6C-DMAEH**	Low	No	Yes	-9,86	2	3	2	1	1	0,17	0	1	2	5,29
**6D-DEAP**	Low	No	Yes	-8,29	1	4	1	2	2	0,55	0	1	2	5,66
**6D-DEAEH**	Low	No	Yes	-8,3	1	4	1	2	2	0,55	0	1	2	5,7
**6F-DMAE**	Low	No	Yes	-9,18	2	4	2	2	2	0,17	0	1	2	5,65
**6F-DMAEH**	Low	No	Yes	-9,19	2	3	2	1	2	0,17	0	1	2	5,69

Supp. Table S12. Site of metabolism predictions. The Atom correspond to the atom numbers indicated on the chemical drawing in the left columns. Rang stands for the range, and Delta P the possibility of that transformation. The most probable transformations (above 0.4) are highlighted in green.

4A

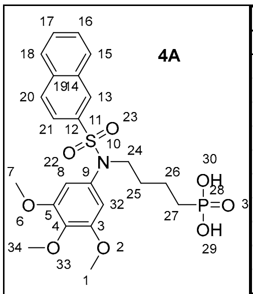

CYP1A2			CYP2C9			CYP2C19			CYP2D6			CYP3A4			UGT		
Atom	Rang	Delta P	Atom	Rang	Delta P	Atom	Rang	Delta P	Atom	Rang	Delta P	Atom	Rang	Delta P	Atom	Rang	Delta P
1	1	0.403	34	1	0.752	1	1	0,707	1	1	0.716	34	1	0.564	10	1	0.062
7	2	0.403	1	2	0.732	7	2	0,707	7	2	0.716	1	2	0.55	29	2	-0.01
34	3	0.277	7	3	0.732	34	3	0,659	34	3	0.712	7	3	0.55	30	3	-0.01
24	4	0.2	24	4	0.402	24	4	0,423	24	4	0.363	24	4	0.469	28	4	-0.052
16	5	0.173	11	5	0.341	16	5	0,267	16	5	0.224	16	5	0.295	31	5	-0.052
17	6	0.173	26	6	0.281	17	6	0,267	17	6	0.224	17	6	0.295	33	6	-0.069
20	7	0.114	16	7	0.269	21	7	0,217	26	7	0.147	18	7	0.19	2	7	-0.09
18	8	0.087	17	8	0.269	26	8	0,161	27	8	0.042	25	8	0.153	6	8	-0.09
15	9	0.035	27	9	0.254	25	9	0,152	18	9	-0.042	20	9	0.147	22	9	-0.113
26	10	-0.079	25	10	0.155	11	10	0,093	25	10	-0.096	15	10	0.125	23	10	-0.113

4B

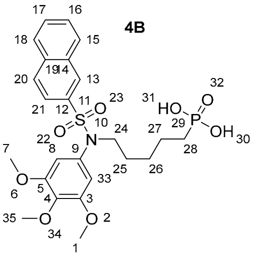

CYP1A2			CYP2C9			CYP2C19			CYP2D6			CYP3A4			UGT		
Atom	Rang	Delta P	Atom	Rang	Delta P	Atom	Rang	Delta P	Atom	Rang	Delta P	Atom	Rang	Delta P	Atom	Rang	Delta P
1	1	0.401	35	1	0.749	1	1	0.703	1	1	0.708	35	1	0.564	10	1	0.058
7	2	0.401	1	2	0.729	7	2	0.703	7	2	0.708	1	2	0.551	30	2	-0.01
35	3	0.278	7	3	0.729	35	3	0.652	35	3	0.703	7	3	0.551	31	3	-0.01
24	4	0.24	24	4	0.421	24	4	0.435	24	4	0.395	24	4	0.503	29	4	-0.051
16	5	0.175	11	5	0.351	16	5	0.268	16	5	0.223	16	5	0.301	32	5	-0.051
17	6	0.175	28	6	0.281	17	6	0.268	17	6	0.223	17	6	0.301	34	6	-0.067
20	7	0.118	27	7	0.279	21	7	0.22	26	7	0.134	18	7	0.2	2	7	-0.088
18	8	0.091	16	8	0.278	26	8	0.194	28	8	0.091	25	8	0.162	6	8	-0.088
15	9	0.041	17	9	0.278	27	9	0.164	27	9	0.077	20	9	0.16	22	9	-0.109
26	10	-0.042	26	10	0.255	25	10	0.157	18	10	-0.035	26	10	0.149	23	10	-0.109

4C

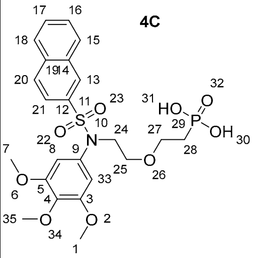

CYP1A2			CYP2C9			CYP2C19			CYP2D6			CYP3A4			UGT		
Atom	Rang	Delta P	Atom	Rang	Delta P	Atom	Rang	Delta P	Atom	Rang	Delta P	Atom	Rang	Delta P	Atom	Rang	Delta P
1	1	0.356	35	1	0.73	1	1	0.684	1	1	0.698	35	1	0.527	10	1	0.263
7	2	0.356	1	2	0.711	7	2	0.684	7	2	0.698	1	2	0.515	30	2	-0.031
35	3	0.214	7	3	0.711	35	3	0.623	35	3	0.694	7	3	0.515	31	3	-0.031
16	4	0.13	11	4	0.304	24	4	0.247	16	4	0.204	24	4	0.29	26	4	-0.043
17	5	0.13	27	5	0.28	16	5	0.229	17	5	0.204	16	5	0.263	29	5	-0.074
20	6	0.069	16	6	0.239	17	6	0.229	27	6	0.184	17	6	0.263	32	6	-0.074
27	7	0.046	17	7	0.239	27	7	0.21	24	7	0.137	25	7	0.193	34	7	-0.088
18	8	0.04	25	8	0.215	25	8	0.206	25	8	0.083	27	8	0.161	2	8	-0.112
25	9	0.026	28	9	0.161	21	9	0.171	28	9	0.028	18	9	0.153	6	9	-0.112
15	10	-0.014	24	10	0.132	11	10	0.015	18	10	-0.066	20	10	0.108	28	10	-0.128

4D

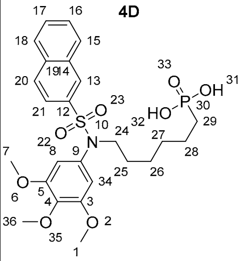

CYP1A2			CYP2C9			CYP2C19			CYP2D6			CYP3A4			UGT		
Atom	Rang	Delta P	Atom	Rang	Delta P	Atom	Rang	Delta P	Atom	Rang	Delta P	Atom	Rang	Delta P	Atom	Rang	Delta P
1	1	0.401	36	1	0.749	1	1	0.703	1	1	0.708	36	1	0.564	10	1	0.058
7	2	0.401	1	2	0.729	7	2	0.703	7	2	0.708	1	2	0.551	31	2	-0.01
36	3	0.278	7	3	0.729	36	3	0.652	36	3	0.703	7	3	0.551	32	3	-0.01
24	4	0.242	24	4	0.427	24	4	0.433	24	4	0.389	24	4	0.505	30	4	-0.051
16	5	0.175	11	5	0.351	16	5	0.268	16	5	0.223	16	5	0.301	33	5	-0.051
17	6	0.175	28	6	0.302	17	6	0.268	17	6	0.223	17	6	0.301	35	6	-0.067
20	7	0.118	29	7	0.291	21	7	0.22	26	7	0.143	25	7	0.217	2	7	-0.088
18	8	0.091	16	8	0.278	26	8	0.196	28	8	0.12	27	8	0.204	6	8	-0.088
15	9	0.041	17	9	0.278	27	9	0.194	29	9	0.094	18	9	0.2	22	9	-0.109
28	10	-0.029	26	10	0.27	28	10	0.187	27	10	0.065	28	10	0.161	23	10	-0.109

4E

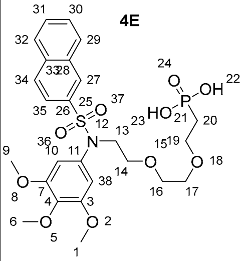

CYP1A2			CYP2C9			CYP2C19			CYP2D6			CYP3A4			UGT		
Atom	Rang	Delta P	Atom	Rang	Delta P	Atom	Rang	Delta P	Atom	Rang	Delta P	Atom	Rang	Delta P	Atom	Rang	Delta P
1	1	0.344	6	1	0.719	1	1	0.676	1	1	0.688	6	1	0.515	12	1	0.263
9	2	0.344	1	2	0.701	9	2	0.676	9	2	0.688	1	2	0.504	22	2	-0.031
17	3	0.275	9	3	0.701	6	3	0.614	6	3	0.684	9	3	0.504	23	3	-0.031
6	4	0.203	17	4	0.527	17	4	0.502	17	4	0.329	17	4	0.331	15	4	-0.045
16	5	0.199	16	5	0.449	16	5	0.457	19	5	0.296	19	5	0.32	18	5	-0.045
19	6	0.149	19	6	0.31	13	6	0.247	16	6	0.255	14	6	0.271	21	6	-0.074
30	7	0.124	25	7	0.295	30	7	0.227	30	7	0.197	16	7	0.263	24	7	-0.074
31	8	0.124	30	8	0.233	31	8	0.227	31	8	0.197	13	8	0.261	5	8	-0.088
14	9	0.07	31	9	0.233	19	9	0.217	14	9	0.129	30	9	0.255	2	9	-0.112
34	10	0.063	20	10	0.16	35	10	0.169	13	10	0.112	31	10	0.255	8	10	-0.112

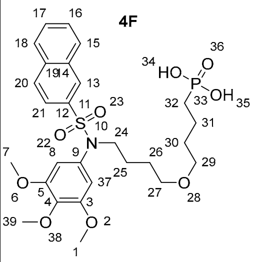

CYP1A2			CYP2C9			CYP2C19			CYP2D6			CYP3A4			UGT		
Atom	Rang	Delta P	Atom	Rang	Delta P	Atom	Rang	Delta P	Atom	Rang	Delta P	Atom	Rang	Delta P	Atom	Rang	Delta P
29	1	0.41	39	1	0.687	1	1	0.654	1	1	0.656	27	1	0.491	10	1	0.057
27	2	0.405	1	2	0.665	7	2	0.654	7	2	0.656	29	2	0.47	28	2	-0.002
1	3	0.268	7	3	0.665	39	3	0.59	39	3	0.65	39	3	0.469	34	3	-0.006
7	4	0.268	27	4	0.571	27	4	0.428	27	4	0.53	1	4	0.457	35	4	-0.006
39	5	0.152	29	5	0.571	29	5	0.427	29	5	0.529	7	5	0.457	33	5	-0.044
24	6	0.089	24	6	0.354	24	6	0.377	24	6	0.353	24	6	0.407	36	6	-0.044
16	7	0.083	11	7	0.263	16	7	0.224	16	7	0.164	16	7	0.215	38	7	-0.059
17	8	0.083	32	8	0.23	17	8	0.224	17	8	0.164	17	8	0.215	2	8	-0.078
20	9	0.019	16	9	0.209	26	9	0.184	26	9	0.093	30	9	0.122	6	9	-0.078
18	10	-0.007	17	10	0.209	30	10	0.181	32	10	0.082	26	10	0.111	22	10	-0.094

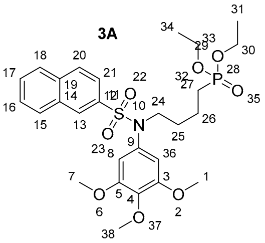

CYP1A2			CYP2C9			CYP2C19			CYP2D6			CYP3A4			UGT		
Atom	Rang	Delta P	Atom	Rang	Delta P	Atom	Rang	Delta P	Atom	Rang	Delta P	Atom	Rang	Delta P	Atom	Rang	Delta P
30	1	0.486	38	1	0.7	1	1	0.649	1	1	0.672	30	1	0.631	10	1	0.041
33	2	0.486	1	2	0.682	7	2	0.649	7	2	0.672	33	2	0.631	29	2	-0.042
1	3	0.294	7	3	0.682	38	3	0.586	38	3	0.668	38	3	0.495	32	3	-0.042
7	4	0.294	30	4	0.376	30	4	0.576	24	4	0.334	1	4	0.485	35	4	-0.049
38	5	0.175	33	5	0.376	33	5	0.576	30	5	0.243	7	5	0.485	37	5	-0.063
24	6	0.12	24	6	0.358	24	6	0.362	33	6	0.243	24	6	0.398	28	6	-0.074
16	7	0.108	11	7	0.307	16	7	0.237	16	7	0.213	16	7	0.258	2	7	-0.082
17	8	0.108	26	8	0.257	17	8	0.237	17	8	0.213	17	8	0.258	6	8	-0.082
20	9	0.051	16	9	0.249	21	9	0.188	26	9	0.145	18	9	0.16	22	9	-0.098
18	10	0.025	17	10	0.249	26	10	0.13	27	10	0.055	25	10	0.122	23	10	-0.098

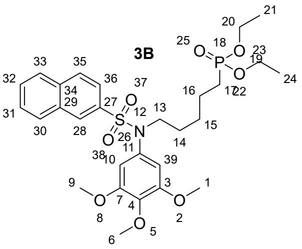

CYP1A2			CYP2C9			CYP2C19			CYP2D6			CYP3A4			UGT		
Atom	Rang	Delta P	Atom	Rang	Delta P	Atom	Rang	Delta P	Atom	Rang	Delta P	Atom	Rang	Delta P	Atom	Rang	Delta P
20	1	0.483	6	1	0.699	1	1	0.644	1	1	0.667	20	1	0.63	12	1	0.039
23	2	0.483	1	2	0.68	9	2	0.644	9	2	0.667	23	2	0.63	19	2	-0.042
1	3	0.294	9	3	0.68	6	3	0.582	6	3	0.662	6	3	0.497	22	3	-0.042
9	4	0.294	20	4	0.382	20	4	0.572	13	4	0.358	1	4	0.487	25	4	-0.048
6	5	0.177	23	5	0.382	23	5	0.572	20	5	0.242	9	5	0.487	5	5	-0.062
13	6	0.148	13	6	0.378	13	6	0.377	23	6	0.242	13	6	0.435	18	6	-0.072
31	7	0.111	26	7	0.316	31	7	0.239	31	7	0.213	31	7	0.264	2	7	-0.08
32	8	0.111	17	8	0.258	32	8	0.239	32	8	0.213	32	8	0.264	8	8	-0.08
35	9	0.056	31	9	0.258	36	9	0.192	15	9	0.133	33	9	0.17	37	9	-0.095
33	10	0.031	32	10	0.258	15	10	0.162	17	10	0.097	14	10	0.132	38	10	-0.095

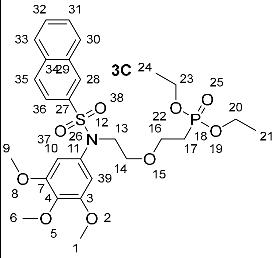

CYP1A2			CYP2C9			CYP2C19			CYP2D6			CYP3A4			UGT		
Atom	Rang	Delta P	Atom	Rang	Delta P	Atom	Rang	Delta P	Atom	Rang	Delta P	Atom	Rang	Delta P	Atom	Rang	Delta P
20	1	0.476	6	1	0.702	1	1	0.64	1	1	0.674	20	1	0.63	12	1	0.217
23	2	0.476	1	2	0.683	9	2	0.64	9	2	0.674	23	2	0.63	15	2	-0.042
1	3	0.273	9	3	0.683	6	3	0.574	6	3	0.67	6	3	0.49	19	3	-0.054
9	4	0.273	20	4	0.368	20	4	0.564	20	4	0.236	1	4	0.48	22	4	-0.054
6	5	0.158	23	5	0.368	23	5	0.564	23	5	0.236	9	5	0.48	25	5	-0.065
31	6	0.09	26	6	0.296	13	6	0.226	31	6	0.206	13	6	0.269	5	6	-0.079
32	7	0.09	16	7	0.275	31	7	0.212	32	7	0.206	31	7	0.246	18	7	-0.094
35	8	0.029	31	8	0.239	32	8	0.212	16	8	0.188	32	8	0.246	2	8	-0.1
16	9	0.004	32	9	0.239	16	9	0.193	13	9	0.146	14	9	0.18	8	9	-0.1
33	10	0.003	14	10	0.216	14	10	0.189	14	10	0.099	16	10	0.151	17	10	-0.109

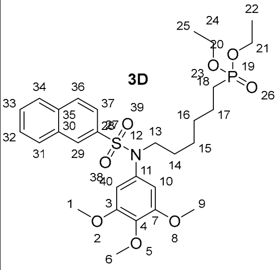

CYP1A2			CYP2C9			CYP2C19			CYP2D6			CYP3A4			UGT		
Atom	Rang	Delta P	Atom	Rang	Delta P	Atom	Rang	Delta P	Atom	Rang	Delta P	Atom	Rang	Delta P	Atom	Rang	Delta P
21	1	0.483	6	1	0.699	1	1	0.644	1	1	0.667	21	1	0.63	12	1	0.039
24	2	0.483	1	2	0.68	9	2	0.644	9	2	0.667	24	2	0.63	20	2	-0.042
1	3	0.294	9	3	0.68	6	3	0.582	6	3	0.662	6	3	0.497	23	3	-0.042
9	4	0.294	13	4	0.385	21	4	0.572	13	4	0.354	1	4	0.487	26	4	-0.048
6	5	0.177	21	5	0.382	24	5	0.572	21	5	0.242	9	5	0.487	5	5	-0.062
13	6	0.15	24	6	0.382	13	6	0.376	24	6	0.242	13	6	0.438	19	6	-0.072
32	7	0.111	27	7	0.316	32	7	0.239	32	7	0.213	32	7	0.264	2	7	-0.08
33	8	0.111	17	8	0.279	33	8	0.239	33	8	0.213	33	8	0.264	8	8	-0.08
36	9	0.056	18	9	0.268	37	9	0.192	15	9	0.141	14	9	0.183	38	9	-0.095
34	10	0.031	32	10	0.258	15	10	0.166	17	10	0.121	16	10	0.171	39	10	-0.095

3E

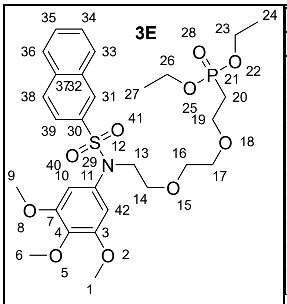

CYP1A2			CYP2C9			CYP2C19			CYP2D6			CYP3A4			UGT		
Atom	Rang	Delta P	Atom	Rang	Delta P	Atom	Rang	Delta P	Atom	Rang	Delta P	Atom	Rang	Delta P	Atom	Rang	Delta P
23	1	0.464	6	1	0.692	1	1	0.632	1	1	0.666	23	1	0.621	12	1	0.217
26	2	0.464	1	2	0.672	9	2	0.632	9	2	0.666	26	2	0.621	15	2	-0.042
1	3	0.262	9	3	0.672	6	3	0.566	6	3	0.662	6	3	0.48	18	3	-0.042
9	4	0.262	17	4	0.506	23	4	0.556	17	4	0.322	1	4	0.47	22	4	-0.054
17	5	0.208	16	5	0.433	26	5	0.556	19	5	0.29	9	5	0.47	25	5	-0.054
6	6	0.152	23	6	0.359	17	6	0.467	16	6	0.253	17	6	0.31	28	6	-0.065
16	7	0.151	26	7	0.359	16	7	0.425	23	7	0.229	19	7	0.299	5	7	-0.079
19	8	0.106	19	8	0.303	13	8	0.227	26	8	0.229	14	8	0.253	21	8	-0.094
34	9	0.085	29	9	0.288	34	9	0.21	34	9	0.2	16	9	0.246	2	9	-0.1
35	10	0.085	34	10	0.233	35	10	0.21	35	10	0.2	13	10	0.243	8	10	-0.1

3F

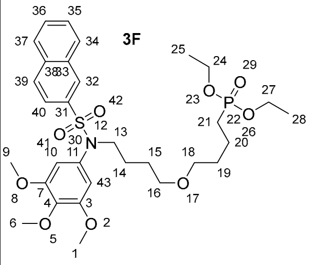

CYP1A2			CYP2C9			CYP2C19			CYP2D6			CYP3A4			UGT		
Atom	Rang	Delta P	Atom	Rang	Delta P	Atom	Rang	Delta P	Atom	Rang	Delta P	Atom	Rang	Delta P	Atom	Rang	Delta P
24	1	0.402	6	1	0.656	1	1	0.611	1	1	0.633	24	1	0.588	12	1	0.049
27	2	0.402	1	2	0.632	9	2	0.611	9	2	0.633	27	2	0.588	17	2	-0.007
18	3	0.351	9	3	0.632	6	3	0.546	6	3	0.627	16	3	0.457	23	3	-0.034
16	4	0.347	18	4	0.551	24	4	0.538	16	4	0.509	18	4	0.435	26	4	-0.034
1	5	0.205	16	5	0.55	27	5	0.538	18	5	0.508	6	5	0.432	29	5	-0.04
9	6	0.205	13	6	0.343	16	6	0.398	13	6	0.343	1	6	0.422	5	6	-0.054
6	7	0.112	24	7	0.333	18	7	0.397	24	7	0.198	9	7	0.422	22	7	-0.062
13	8	0.048	27	8	0.333	13	8	0.34	27	8	0.198	13	8	0.374	2	8	-0.071
35	9	0.045	30	9	0.26	35	9	0.208	35	9	0.169	35	9	0.202	8	9	-0.071
36	10	0.045	21	10	0.23	36	10	0.208	36	10	0.169	36	10	0.202	41	10	-0.083

6A-DMAE

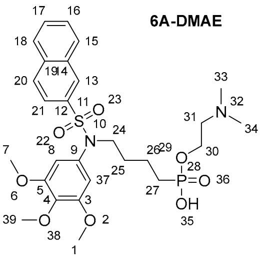

CYP1A2			CYP2C9			CYP2C19			CYP2D6			CYP3A4			UGT		
Atom	Rang	Delta P	Atom	Rang	Delta P	Atom	Rang	Delta P	Atom	Rang	Delta P	Atom	Rang	Delta P	Atom	Rang	Delta P
33	1	0.71	33	1	0.815	33	1	0.757	33	1	0.754	33	1	0.825	32	1	0.197
34	2	0.71	34	2	0.815	34	2	0.757	34	2	0.754	34	2	0.825	10	2	-0.001
1	3	0.201	32	3	0.799	1	3	0.574	1	3	0.622	39	3	0.404	35	3	-0.036
7	4	0.201	39	4	0.629	7	4	0.574	7	4	0.622	1	4	0.394	29	4	-0.058
39	5	0.109	1	5	0.605	39	5	0.505	39	5	0.615	7	5	0.394	36	5	-0.073
30	6	0.082	7	6	0.605	30	6	0.301	24	6	0.253	30	6	0.322	38	6	-0.085
24	7	0.047	24	7	0.282	24	7	0.271	31	7	0.179	31	7	0.319	28	7	-0.086
16	8	0.042	31	8	0.228	31	8	0.202	16	8	0.149	24	8	0.3	2	8	-0.104
17	9	0.042	11	9	0.216	16	9	0.151	17	9	0.149	16	9	0.17	6	9	-0.104
20	10	-0.017	16	10	0.175	17	10	0.151	26	10	0.066	17	10	0.17	22	10	-0.125

6A-DEAP

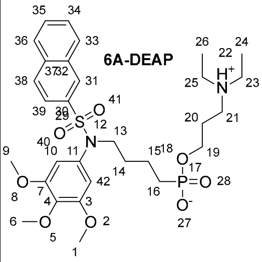

CYP1A2			CYP2C9			CYP2C19			CYP2D6			CYP3A4			UGT		
Atom	Rang	Delta P	Atom	Rang	Delta P	Atom	Rang	Delta P	Atom	Rang	Delta P	Atom	Rang	Delta P	Atom	Rang	Delta P
23	1	0.52	23	1	0.715	23	1	0.684	23	1	0.667	23	1	0.757	12	1	0.021
25	2	0.52	25	2	0.715	25	2	0.684	25	2	0.667	25	2	0.757	27	2	-0.017
21	3	0.432	6	3	0.61	1	3	0.567	1	3	0.597	21	3	0.487	18	3	-0.031
19	4	0.186	1	4	0.588	9	4	0.567	9	4	0.597	6	4	0.388	22	4	-0.038
1	5	0.182	9	5	0.588	6	5	0.5	6	5	0.588	1	5	0.379	28	5	-0.051
9	6	0.182	21	6	0.399	21	6	0.397	21	6	0.348	9	6	0.379	17	6	-0.062
6	7	0.096	22	7	0.297	19	7	0.325	13	7	0.233	19	7	0.368	5	7	-0.064
13	8	0.033	13	8	0.275	13	8	0.273	34	8	0.133	13	8	0.289	2	8	-0.081
34	9	0.029	29	9	0.211	34	9	0.16	35	9	0.133	34	9	0.162	8	9	-0.081
35	10	0.029	15	10	0.176	35	10	0.16	15	10	0.049	35	10	0.162	40	10	-0.096

6A-Chol

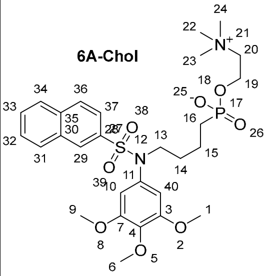

CYP1A2			CYP2C9			CYP2C19			CYP2D6			CYP3A4			UGT		
Atom	Rang	Delta P	Atom	Rang	Delta P	Atom	Rang	Delta P	Atom	Rang	Delta P	Atom	Rang	Delta P	Atom	Rang	Delta P
1	1	0.304	6	1	0.701	1	1	0.647	1	1	0.671	19	1	0.494	21	1	0.03
9	2	0.304	1	2	0.682	9	2	0.647	9	2	0.671	6	2	0.491	12	2	0.018
19	3	0.243	9	3	0.682	6	3	0.585	6	3	0.667	1	3	0.481	25	3	-0.024
6	4	0.182	22	4	0.526	19	4	0.443	22	4	0.384	9	4	0.481	18	4	-0.039
22	5	0.168	23	5	0.526	22	5	0.437	23	5	0.384	22	5	0.426	26	5	-0.062
23	6	0.168	24	6	0.526	23	6	0.437	24	6	0.384	23	6	0.426	17	6	-0.075
24	7	0.168	21	7	0.494	24	7	0.437	13	7	0.325	24	7	0.426	5	7	-0.076
13	8	0.124	13	8	0.359	13	8	0.355	32	8	0.21	13	8	0.382	2	8	-0.095
32	9	0.114	27	9	0.307	32	9	0.235	33	9	0.21	32	9	0.253	8	9	-0.095
33	10	0.114	20	10	0.268	33	10	0.235	20	10	0.142	33	10	0.253	22	10	-0.104

6B-DEAP

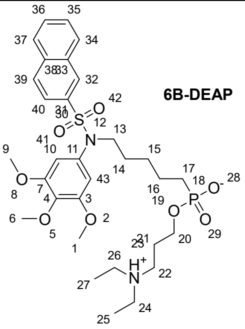

CYP1A2			CYP2C9			CYP2C19			CYP2D6			CYP3A4			UGT		
Atom	Rang	Delta P	Atom	Rang	Delta P	Atom	Rang	Delta P	Atom	Rang	Delta P	Atom	Rang	Delta P	Atom	Rang	Delta P
24	1	0.517	24	1	0.713	24	1	0.679	24	1	0.662	24	1	0.755	12	1	0.02
26	2	0.517	26	2	0.713	26	2	0.679	26	2	0.662	26	2	0.755	28	2	-0.017
22	3	0.43	6	3	0.61	1	3	0.563	1	3	0.59	22	3	0.489	19	3	-0.031
20	4	0.188	1	4	0.588	9	4	0.563	9	4	0.59	6	4	0.392	23	4	-0.038
1	5	0.184	9	5	0.588	6	5	0.497	6	5	0.58	1	5	0.383	29	5	-0.05
9	6	0.184	22	6	0.405	22	6	0.396	22	6	0.346	9	6	0.383	18	6	-0.061
6	7	0.099	23	7	0.305	20	7	0.325	13	7	0.26	20	7	0.373	5	7	-0.063
13	8	0.064	13	8	0.293	13	8	0.286	35	8	0.134	13	8	0.32	2	8	-0.08
35	9	0.034	30	9	0.223	35	9	0.164	36	9	0.134	35	9	0.171	8	9	-0.08
36	10	0.034	35	10	0.182	36	10	0.164	15	10	0.04	36	10	0.171	41	10	-0.093

6C-DMAE

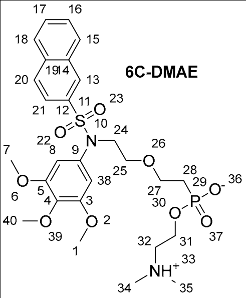

CYP1A2			CYP2C9			CYP2C19			CYP2D6			CYP3A4			UGT		
Atom	Rang	Delta P	Atom	Rang	Delta P	Atom	Rang	Delta P	Atom	Rang	Delta P	Atom	Rang	Delta P	Atom	Rang	Delta P
34	1	0.707	34	1	0.818	34	1	0.753	34	1	0.758	34	1	0.827	33	1	0.182
35	2	0.707	35	2	0.818	35	2	0.753	35	2	0.758	35	2	0.827	10	2	0.11
1	3	0.184	33	3	0.802	1	3	0.562	1	3	0.623	40	3	0.396	36	3	-0.049
7	4	0.184	40	4	0.629	7	4	0.562	7	4	0.623	1	4	0.386	26	4	-0.064
40	5	0.092	1	5	0.604	40	5	0.491	40	5	0.616	7	5	0.386	30	5	-0.076
31	6	0.037	7	6	0.604	31	6	0.267	32	6	0.159	31	6	0.294	37	6	-0.092
16	7	0.021	11	7	0.201	32	7	0.158	16	7	0.14	32	7	0.291	39	7	-0.102
17	8	0.021	32	8	0.199	24	8	0.128	17	8	0.14	24	8	0.168	29	8	-0.106
20	9	-0.037	27	9	0.19	16	9	0.114	27	9	0.113	16	9	0.156	2	9	-0.124
18	10	-0.06	16	10	0.163	17	10	0.114	24	10	0.072	17	10	0.156	6	10	-0.124

6D-DEAP

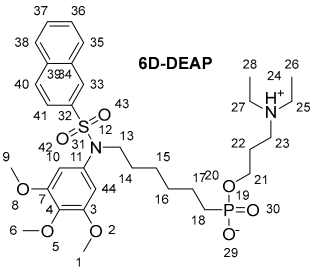

CYP1A2			CYP2C9			CYP2C19			CYP2D6			CYP3A4			UGT		
Atom	Rang	Delta P	Atom	Rang	Delta P	Atom	Rang	Delta P	Atom	Rang	Delta P	Atom	Rang	Delta P	Atom	Rang	Delta P
25	1	0.517	25	1	0.713	25	1	0.679	25	1	0.662	25	1	0.755	12	1	0.02
27	2	0.517	27	2	0.713	27	2	0.679	27	2	0.662	27	2	0.755	29	2	-0.017
23	3	0.43	6	3	0.61	1	3	0.563	1	3	0.59	23	3	0.489	20	3	-0.031
21	4	0.188	1	4	0.588	9	4	0.563	9	4	0.59	6	4	0.392	24	4	-0.038
1	5	0.184	9	5	0.588	6	5	0.497	6	5	0.58	1	5	0.383	30	5	-0.05
9	6	0.184	23	6	0.405	23	6	0.396	23	6	0.346	9	6	0.383	19	6	-0.061
6	7	0.099	24	7	0.305	21	7	0.325	13	7	0.259	21	7	0.373	5	7	-0.063
13	8	0.069	13	8	0.3	13	8	0.286	36	8	0.134	13	8	0.325	2	8	-0.08
36	9	0.034	31	9	0.223	36	9	0.164	37	9	0.134	36	9	0.171	8	9	-0.08
37	10	0.034	17	10	0.198	37	10	0.164	15	10	0.053	37	10	0.171	42	10	-0.093

6F-DMAE

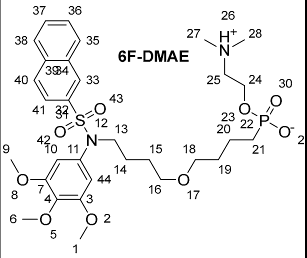

CYP1A2			CYP2C9			CYP2C19			CYP2D6			CYP3A4			UGT		
Atom	Rang	Delta P	Atom	Rang	Delta P	Atom	Rang	Delta P	Atom	Rang	Delta P	Atom	Rang	Delta P	Atom	Rang	Delta P
27	1	0.636	27	1	0.79	27	1	0.732	27	1	0.707	27	1	0.782	26	1	0.2
28	2	0.636	28	2	0.79	28	2	0.732	28	2	0.707	28	2	0.782	12	2	0.007
18	3	0.258	26	3	0.776	1	3	0.536	1	3	0.569	16	3	0.371	29	3	-0.029
16	4	0.253	6	4	0.581	9	4	0.536	9	4	0.569	18	4	0.35	17	4	-0.032
1	5	0.139	1	5	0.562	6	5	0.471	6	5	0.56	6	5	0.345	23	5	-0.051
9	6	0.139	9	6	0.562	16	6	0.311	16	6	0.45	1	6	0.338	30	6	-0.062
6	7	0.047	16	7	0.49	18	7	0.31	18	7	0.448	9	7	0.338	22	7	-0.073
24	8	-0.007	18	8	0.49	13	8	0.261	13	8	0.271	13	8	0.288	5	8	-0.074
36	9	-0.018	13	9	0.274	24	9	0.26	25	9	0.123	24	9	0.25	2	9	-0.091
37	10	-0.018	25	10	0.174	25	10	0.159	36	10	0.106	25	10	0.247	8	10	-0.091

Supp. Fig. S3–S38. ^{1}H and ^{13}C-NMR spectra of the compounds.

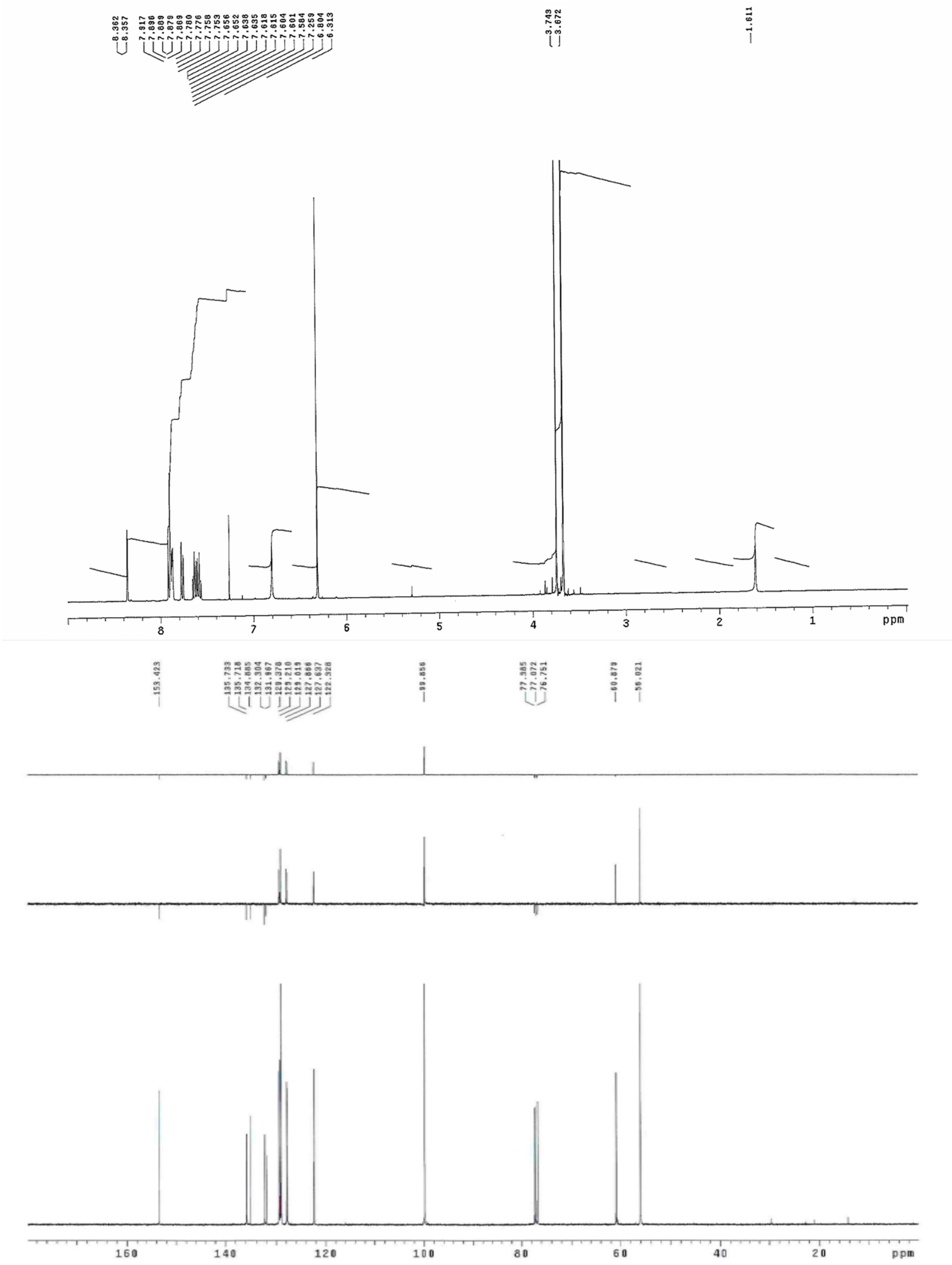

Supp. Fig. S3. ^{1}H and ^{13}C-NMR spectra for compound **1**.

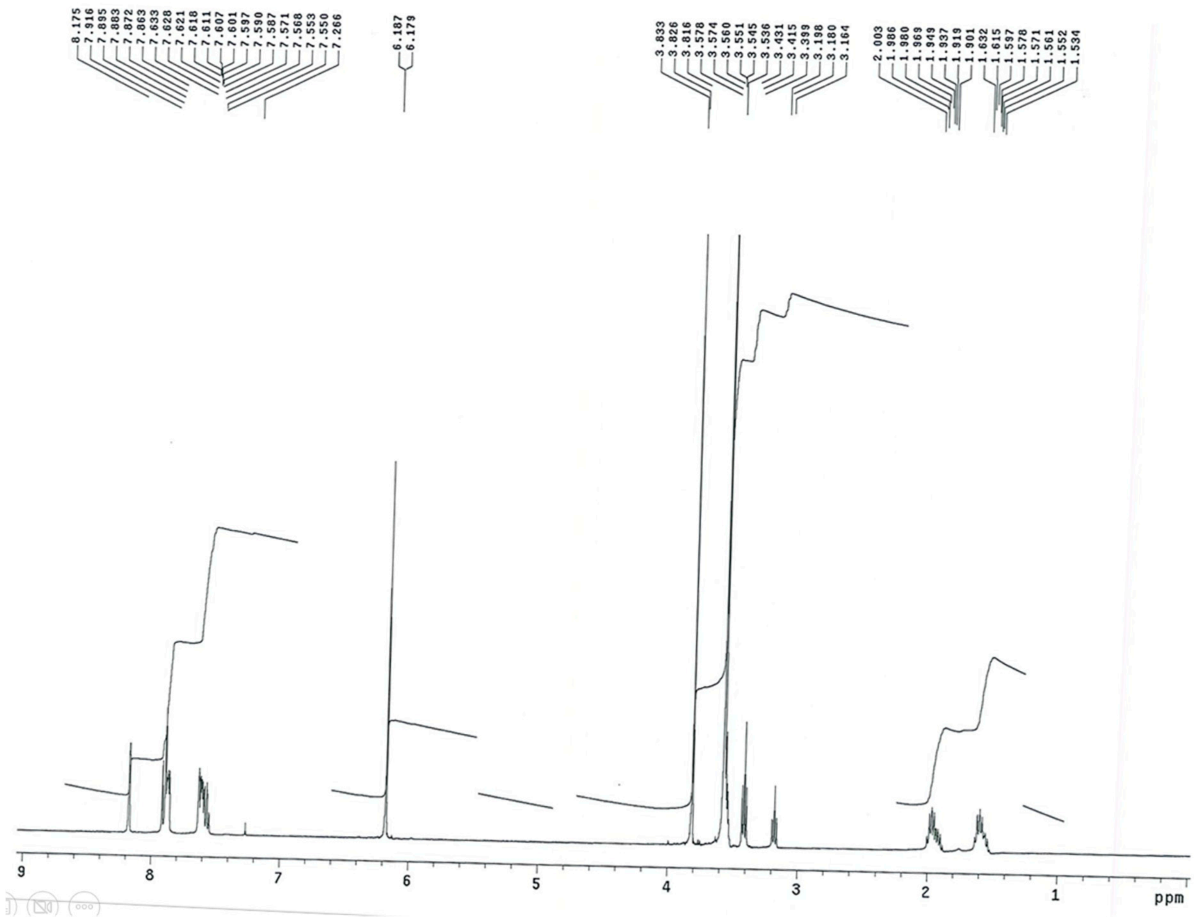

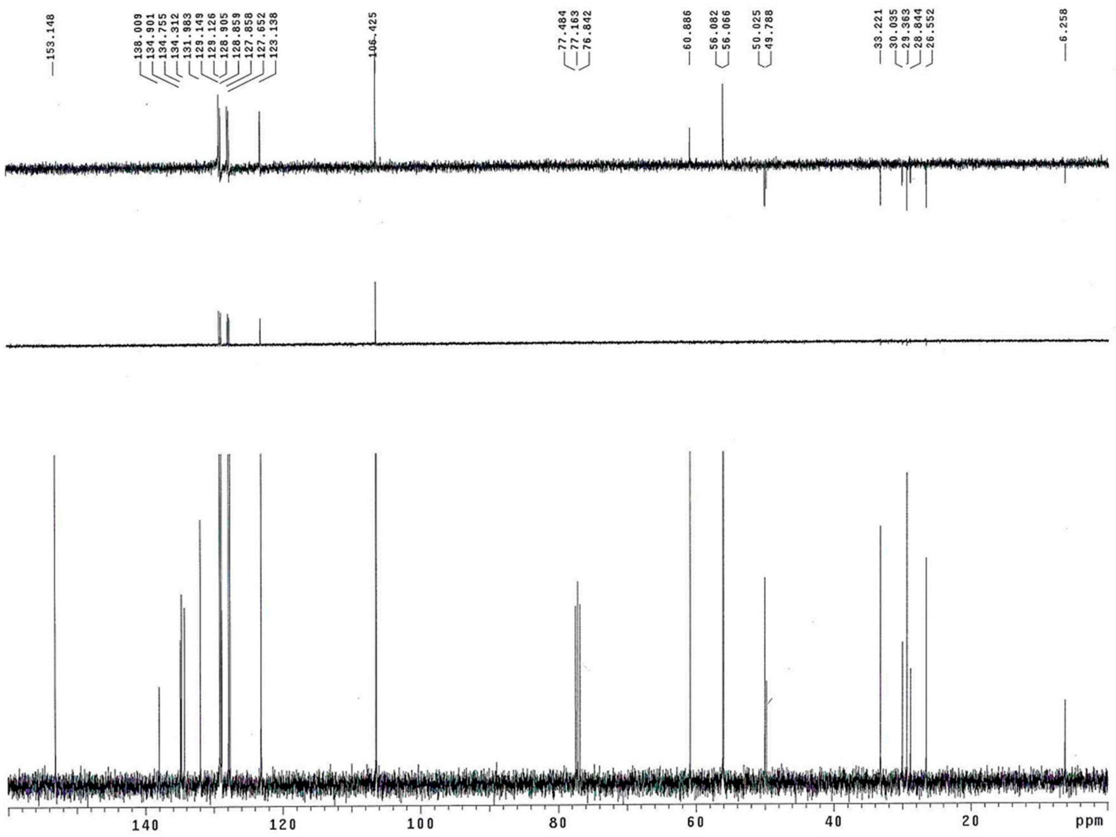

Supp. Fig. S4. ^{1}H and ^{13}C-NMR spectra for compound **2A**.

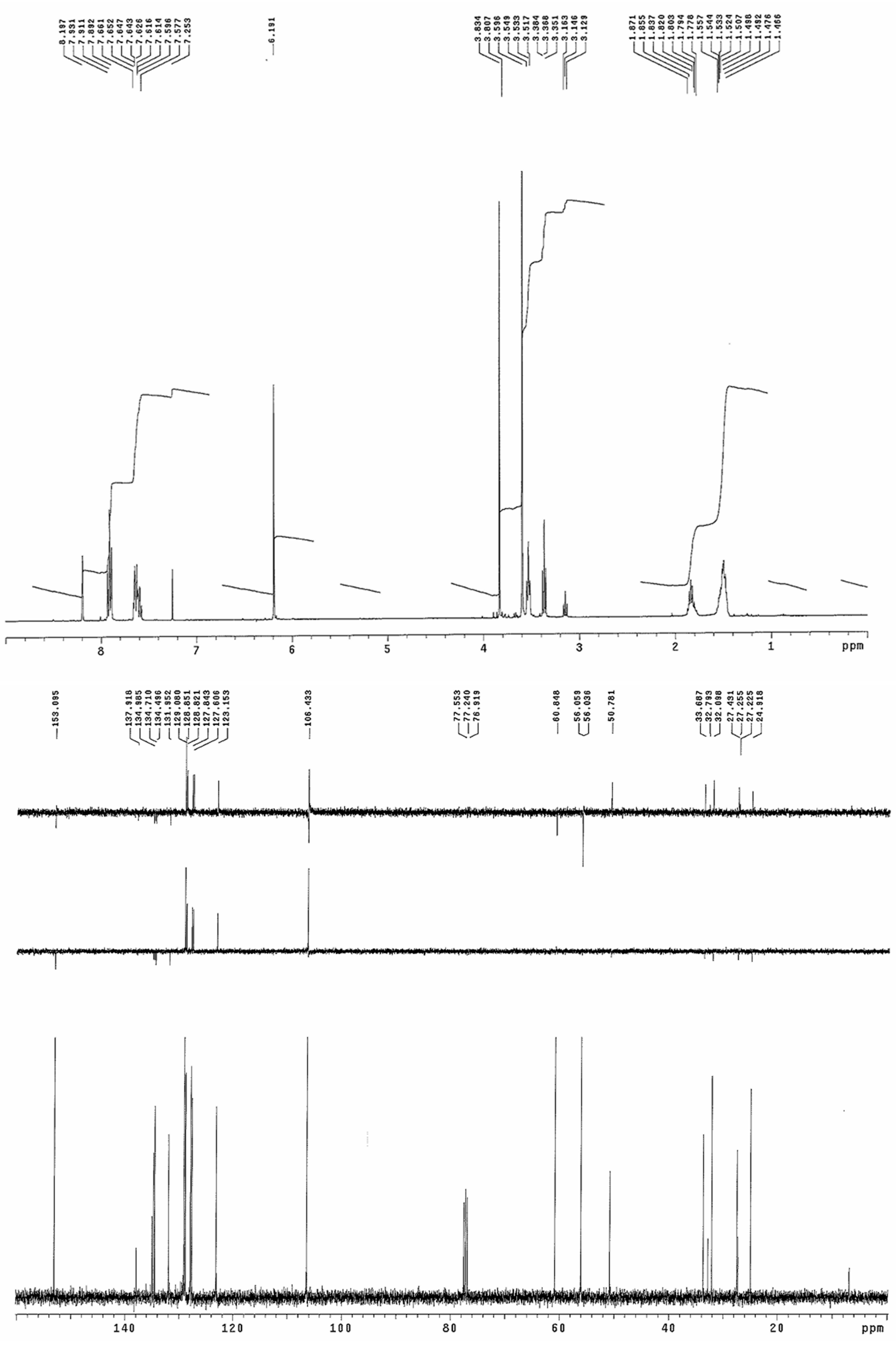

Supp. Fig. S5. ^{1}H and ^{13}C-NMR spectra for compound **2B**.

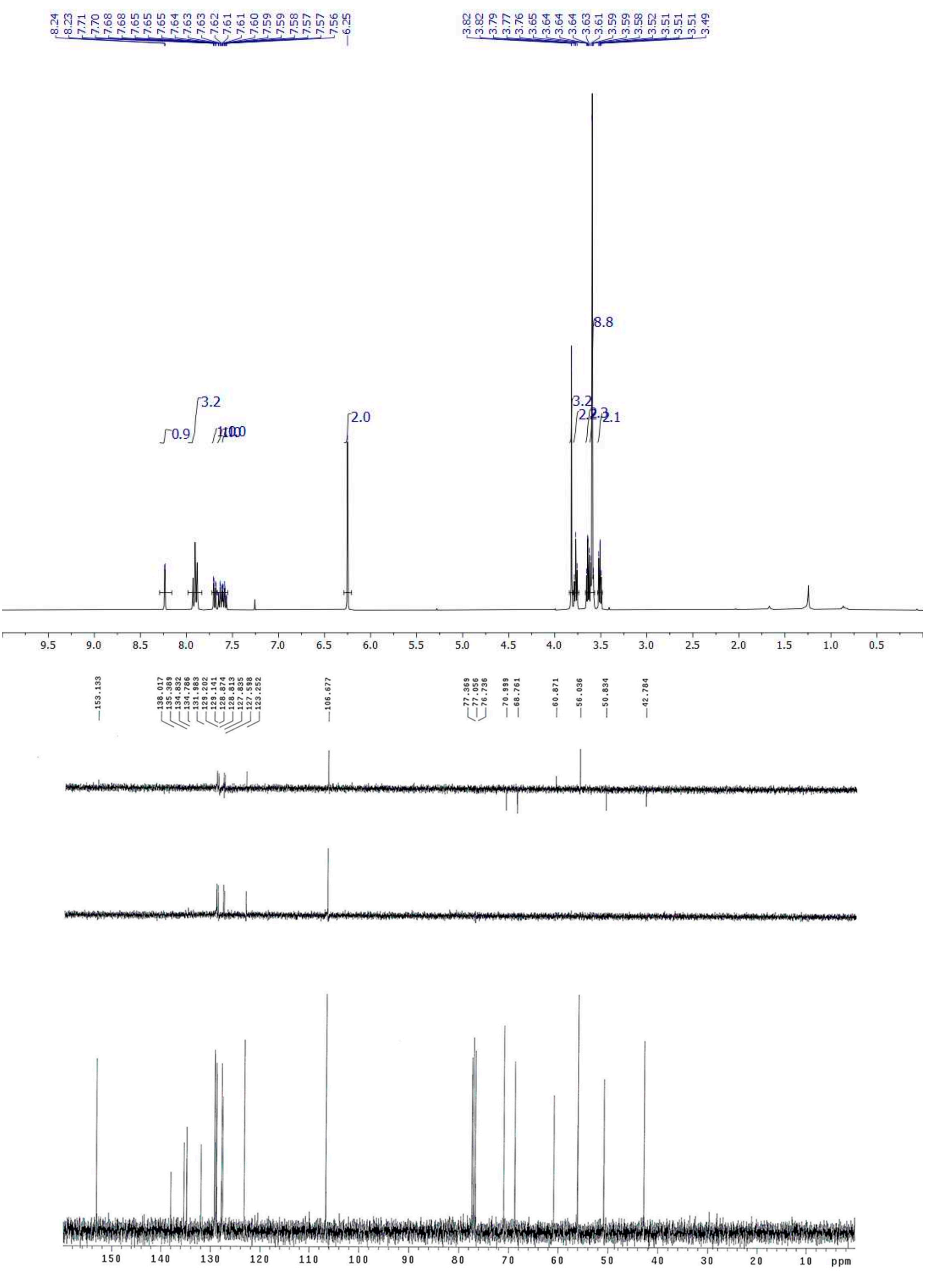

Supp. Fig. S6. ^{1}H and ^{13}C-NMR spectra for compound **2C**.

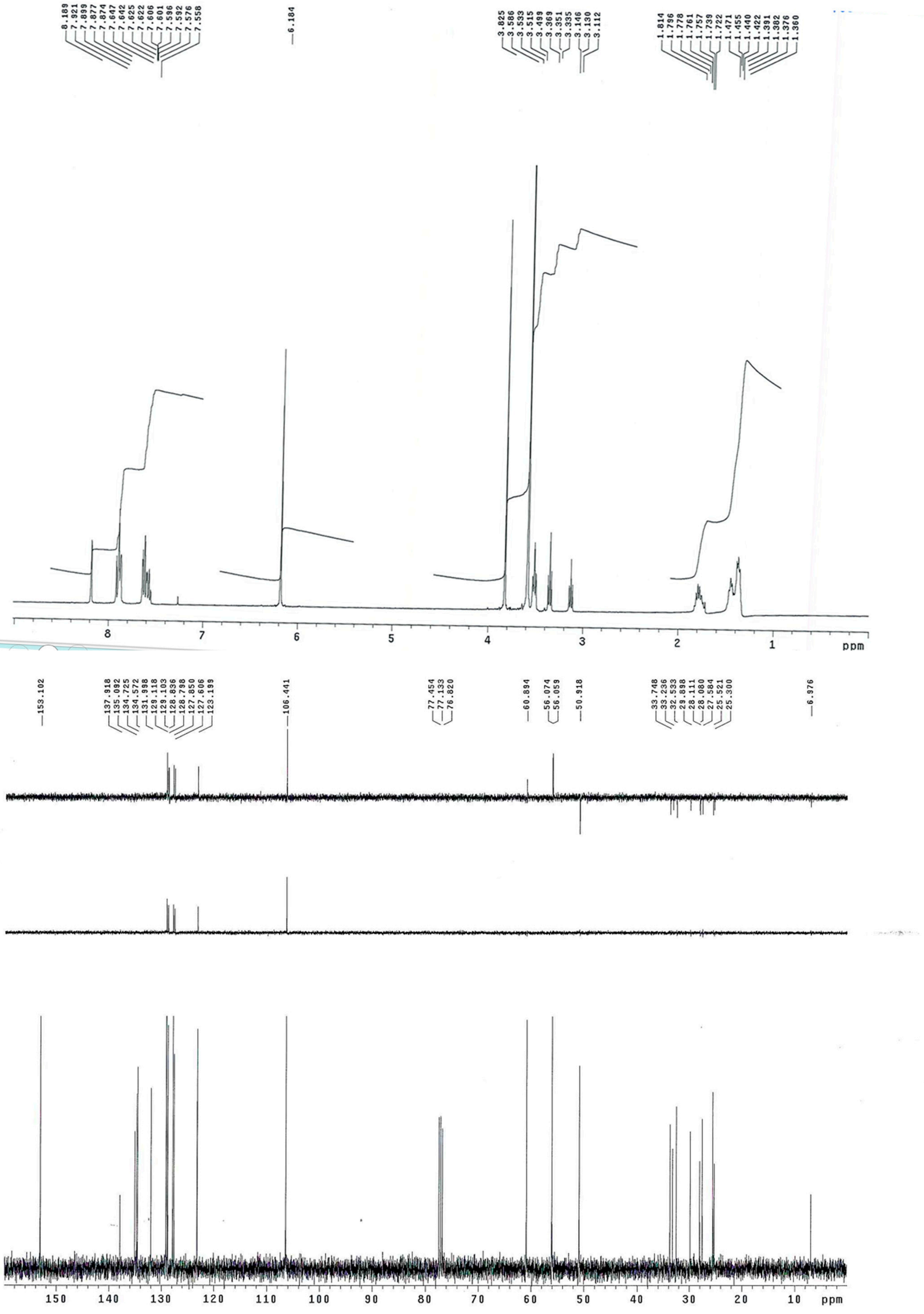

Supp. Fig. S7. ^{1}H and ^{13}C-NMR spectra for compound **2D**.

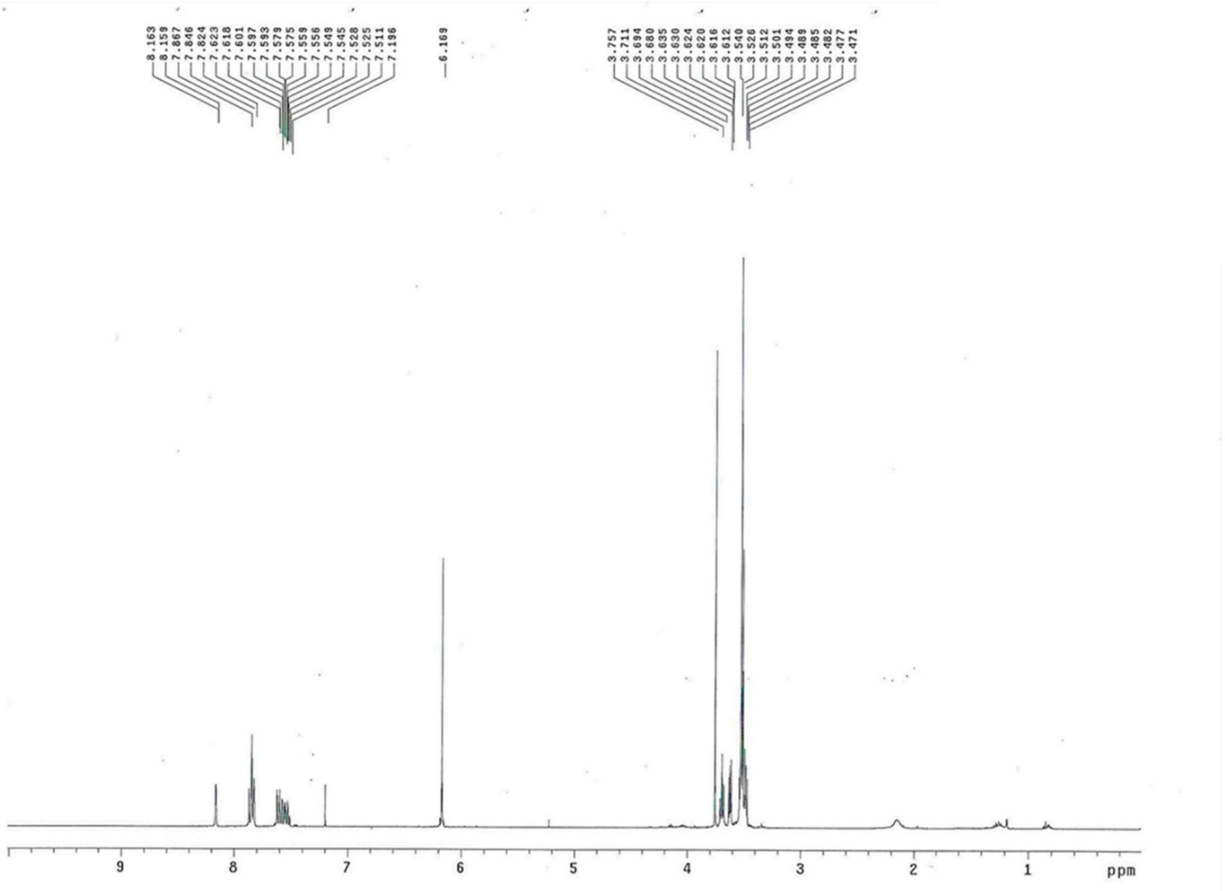

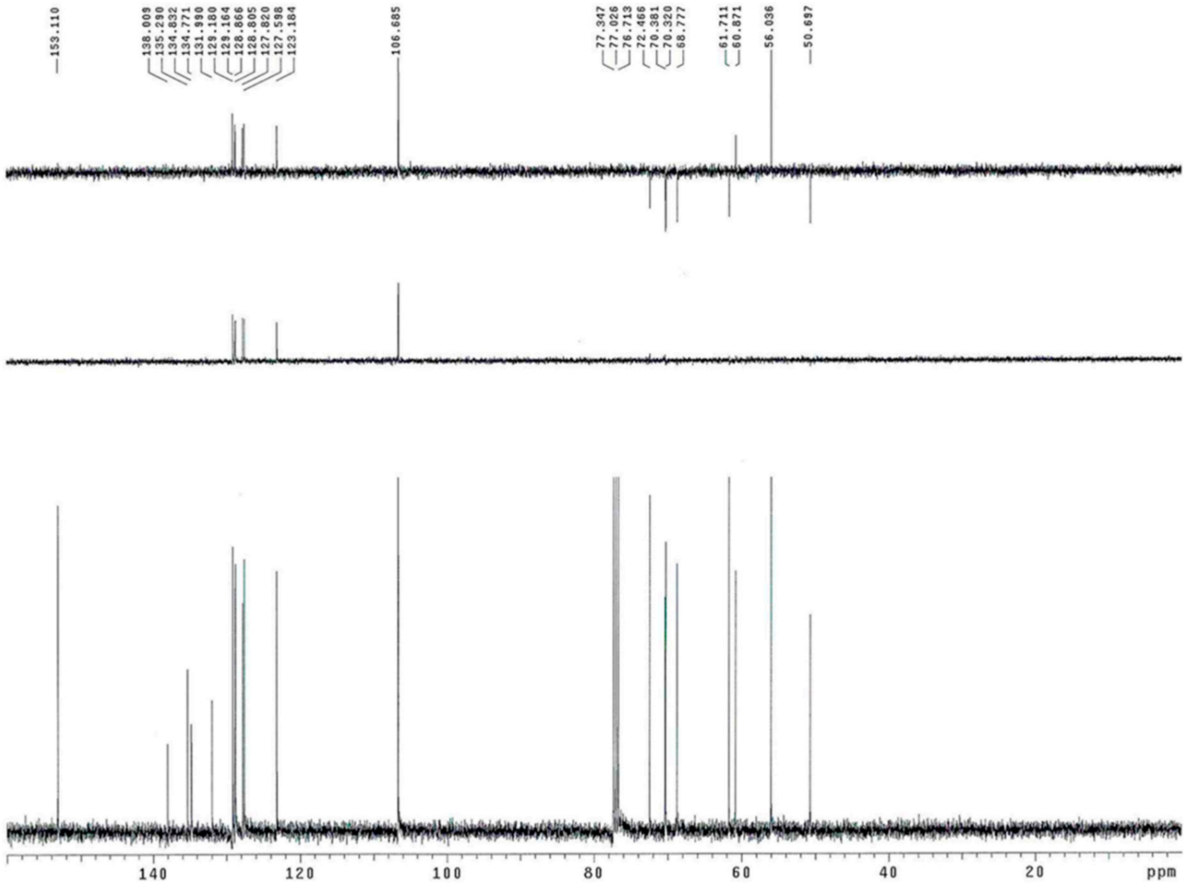

Supp. Fig. S8. ^{1}H and ^{13}C-NMR spectra for compound **2E**.

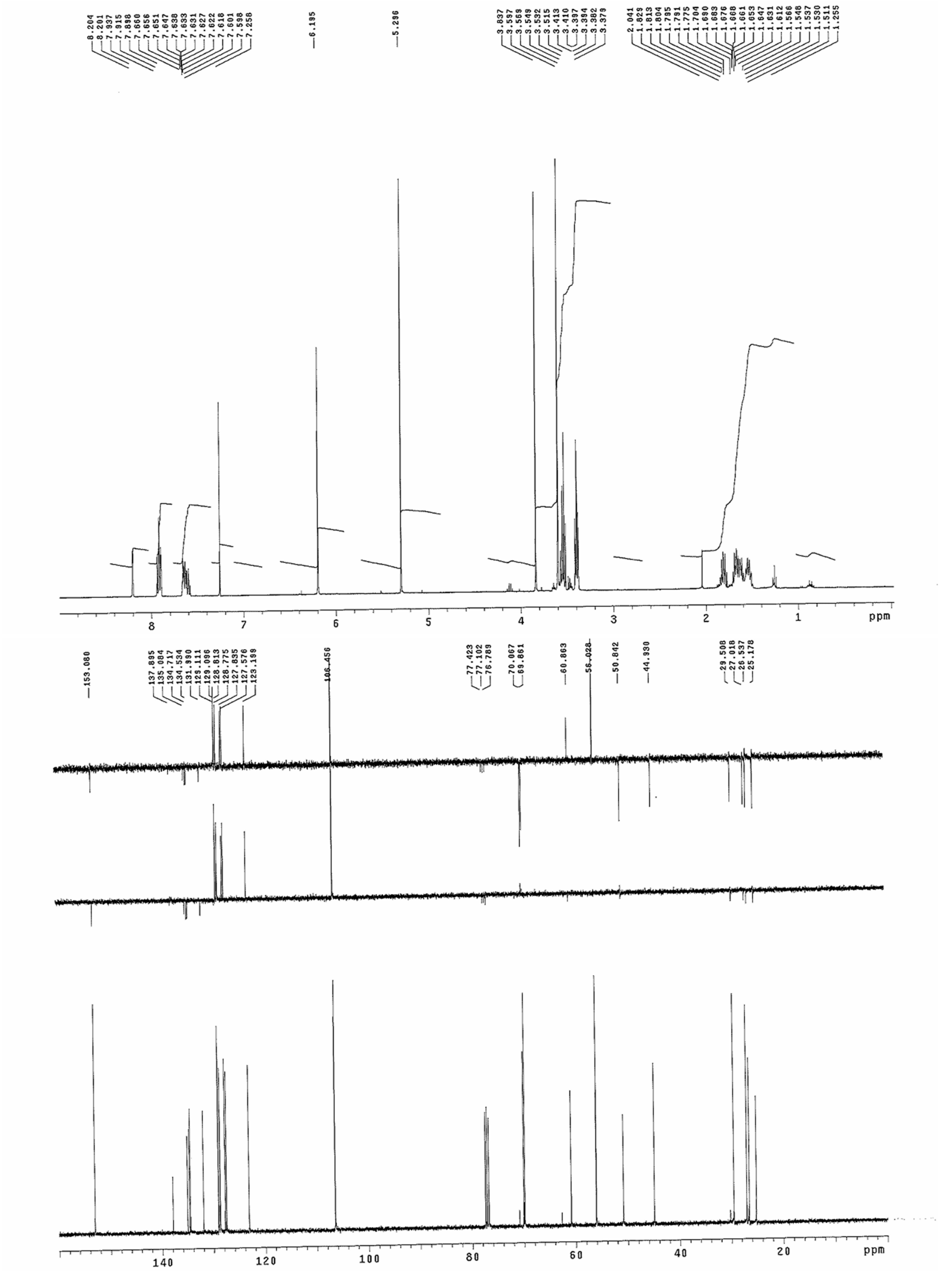

Supp. Fig. S9. ^{1}H and ^{13}C-NMR spectra for compound **2F**.

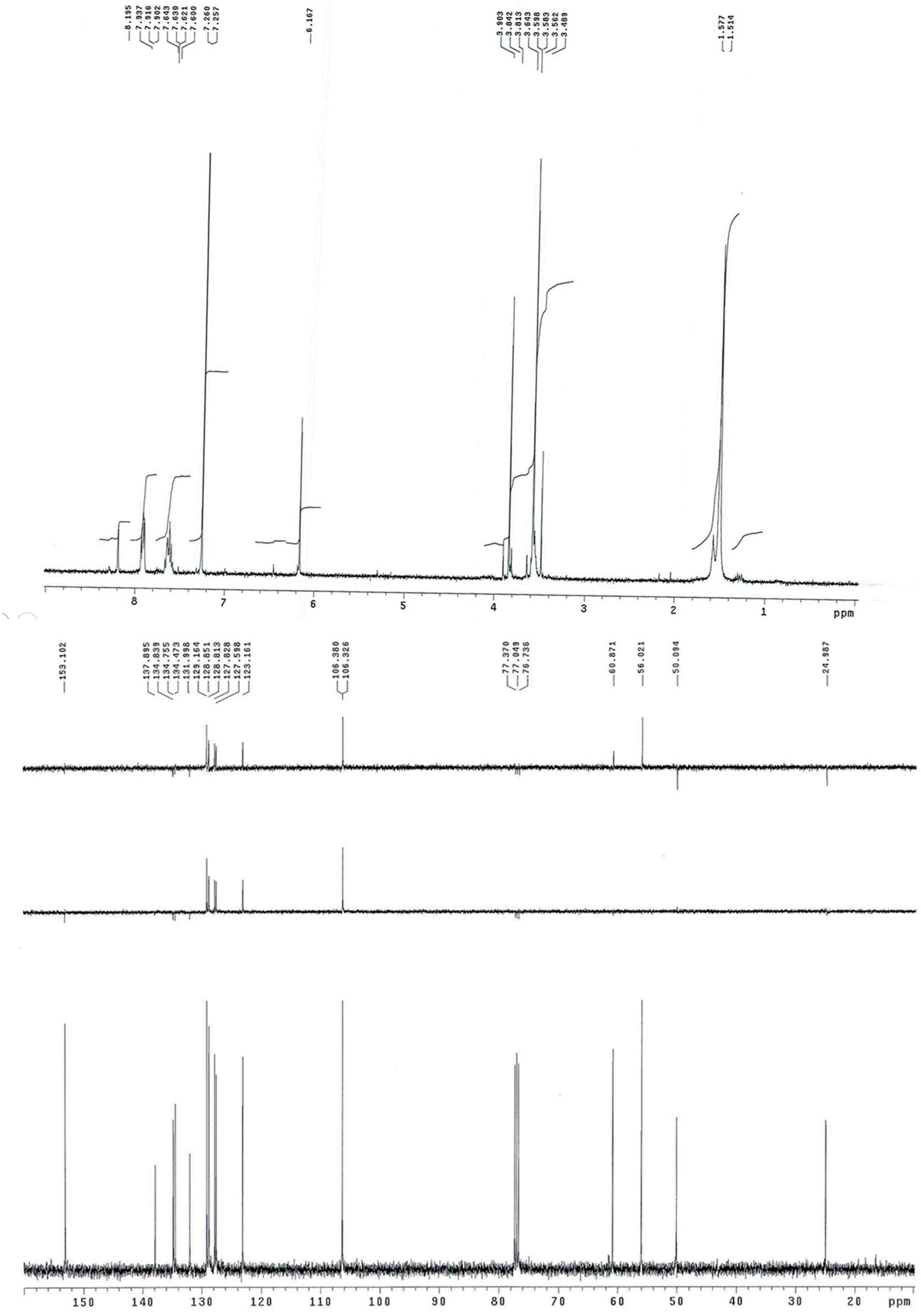

Supp. Fig. S10. ^{1}H and ^{13}C-NMR spectra for compound **2bA**.

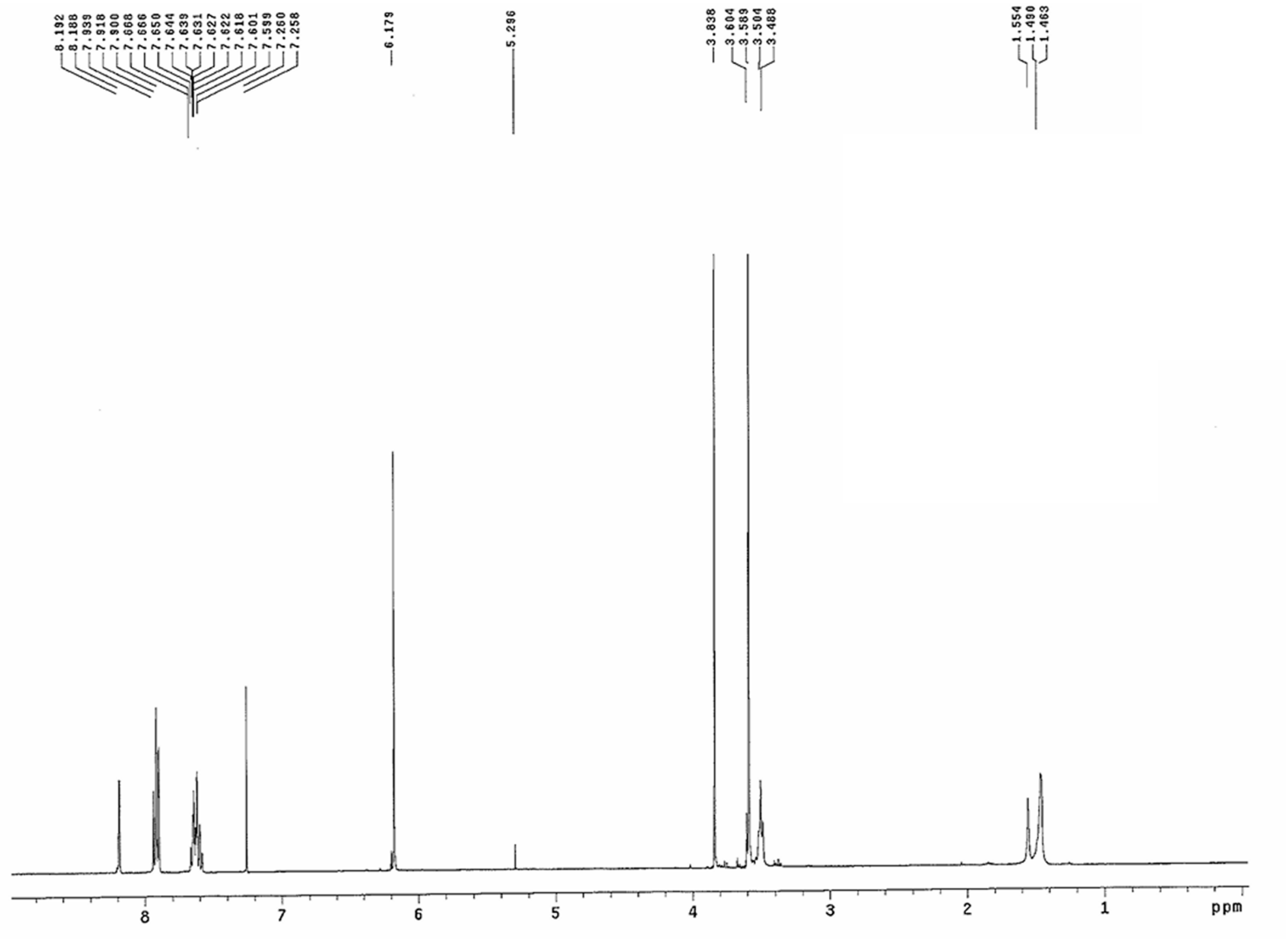

Supp. Fig. S11. ^{1}H-NMR spectrum for compound **2bB**.

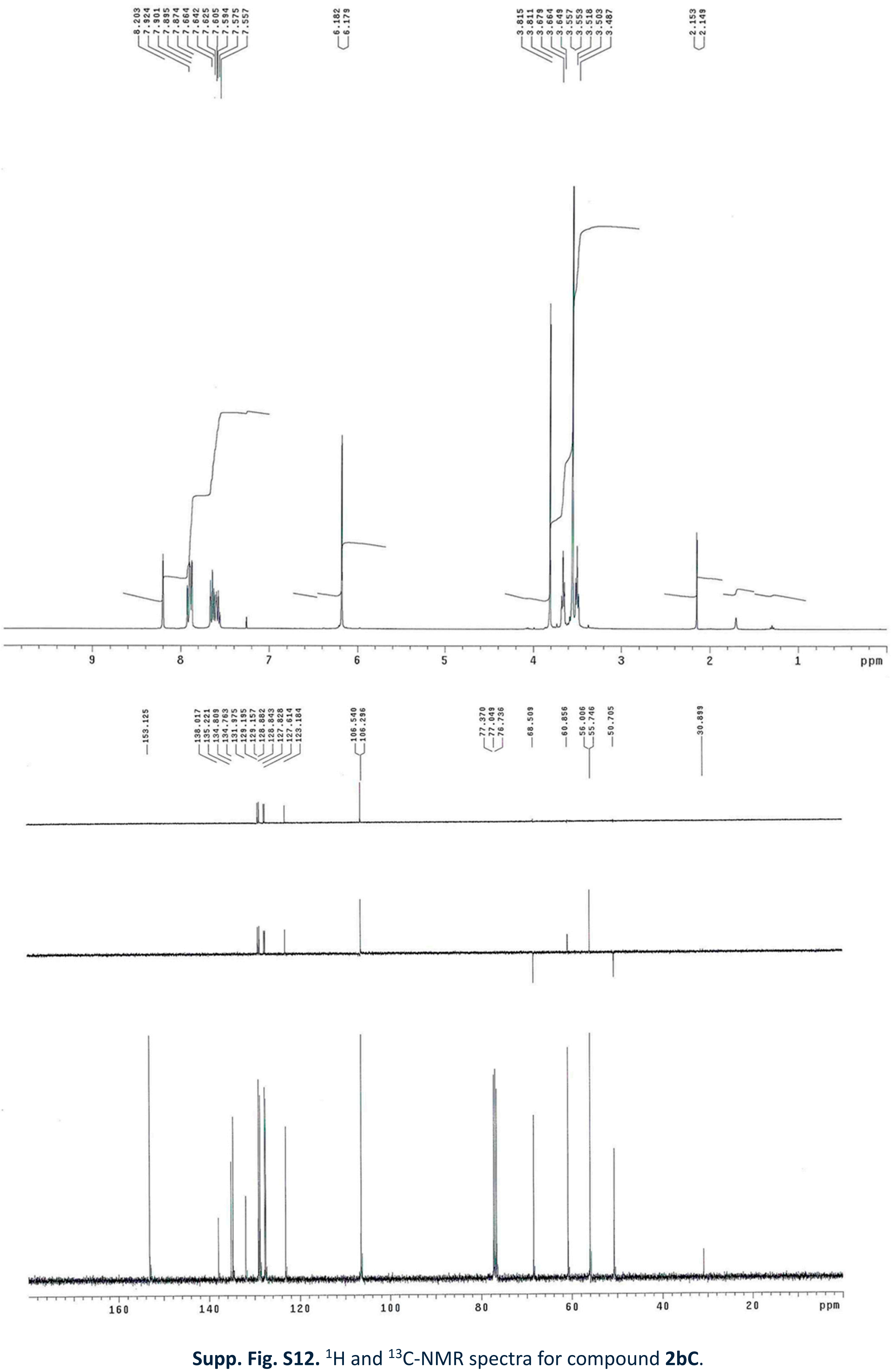

Supp. Fig. S12. ^{1}H and ^{13}C-NMR spectra for compound **2bC**.

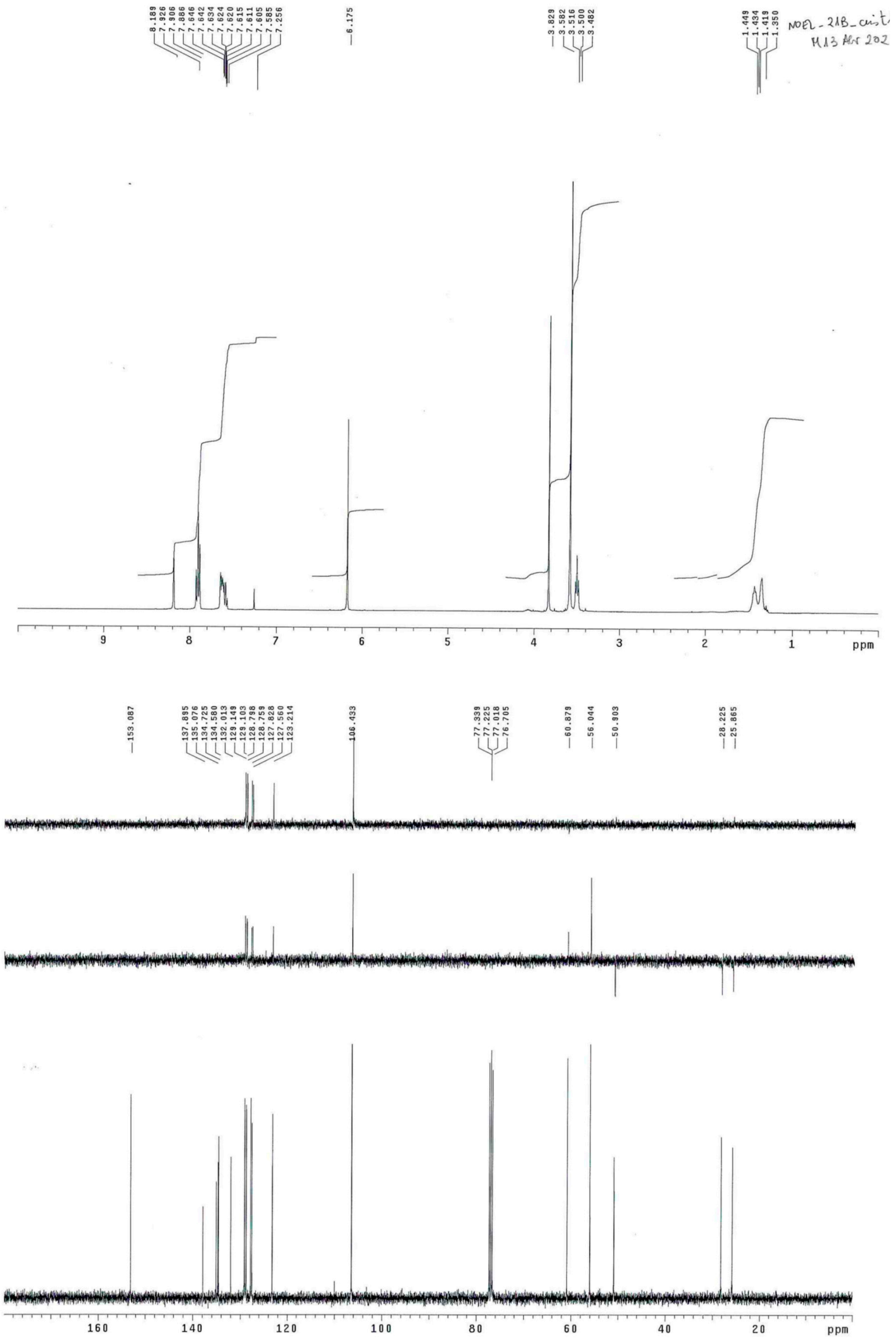

Supp. Fig. S13. ^{1}H and ^{13}C-NMR spectra for compound **2bD**.

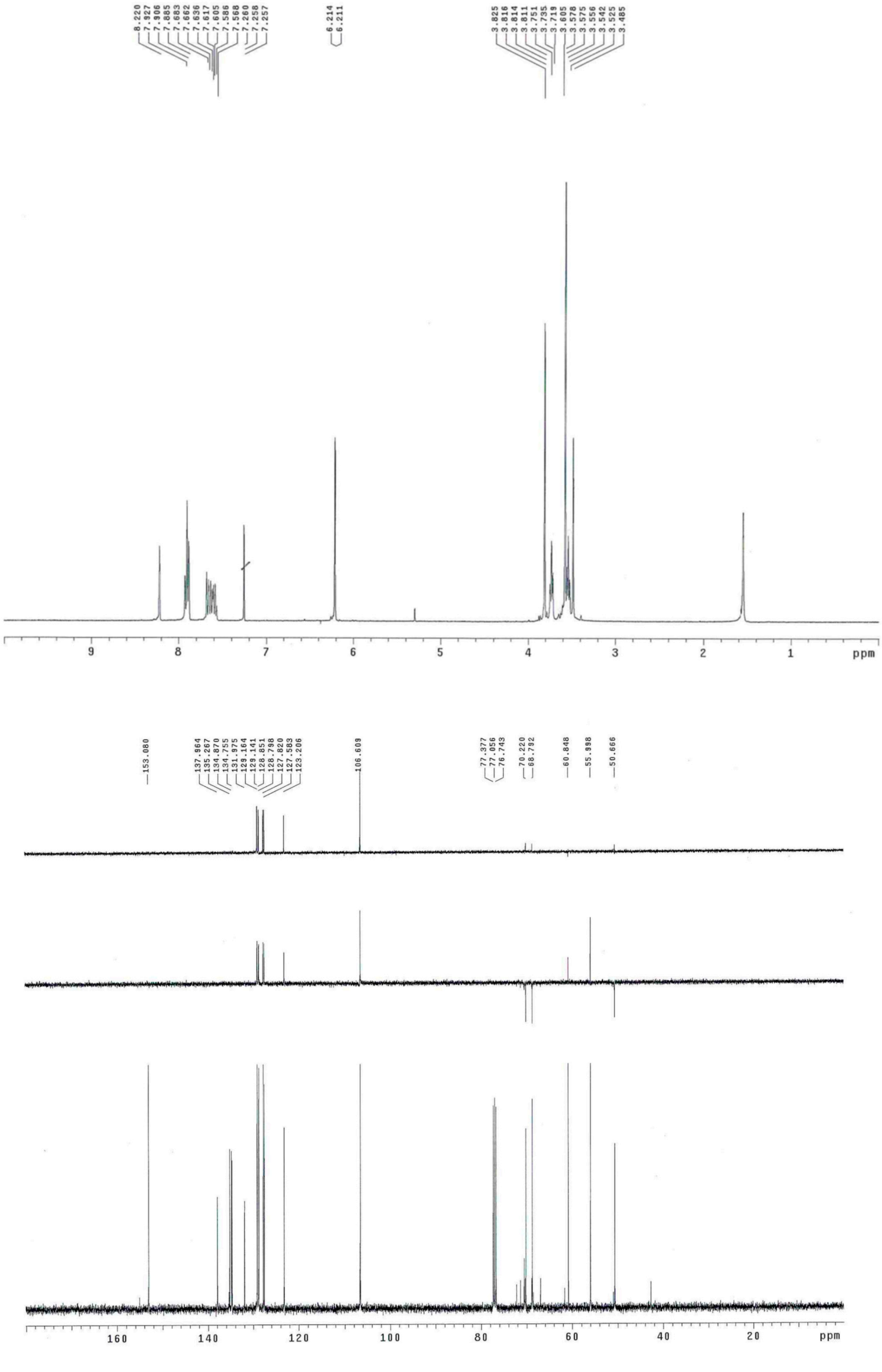

Supp. Fig. S14. ^{1}H and ^{13}C-NMR spectra for compound **2bE**.

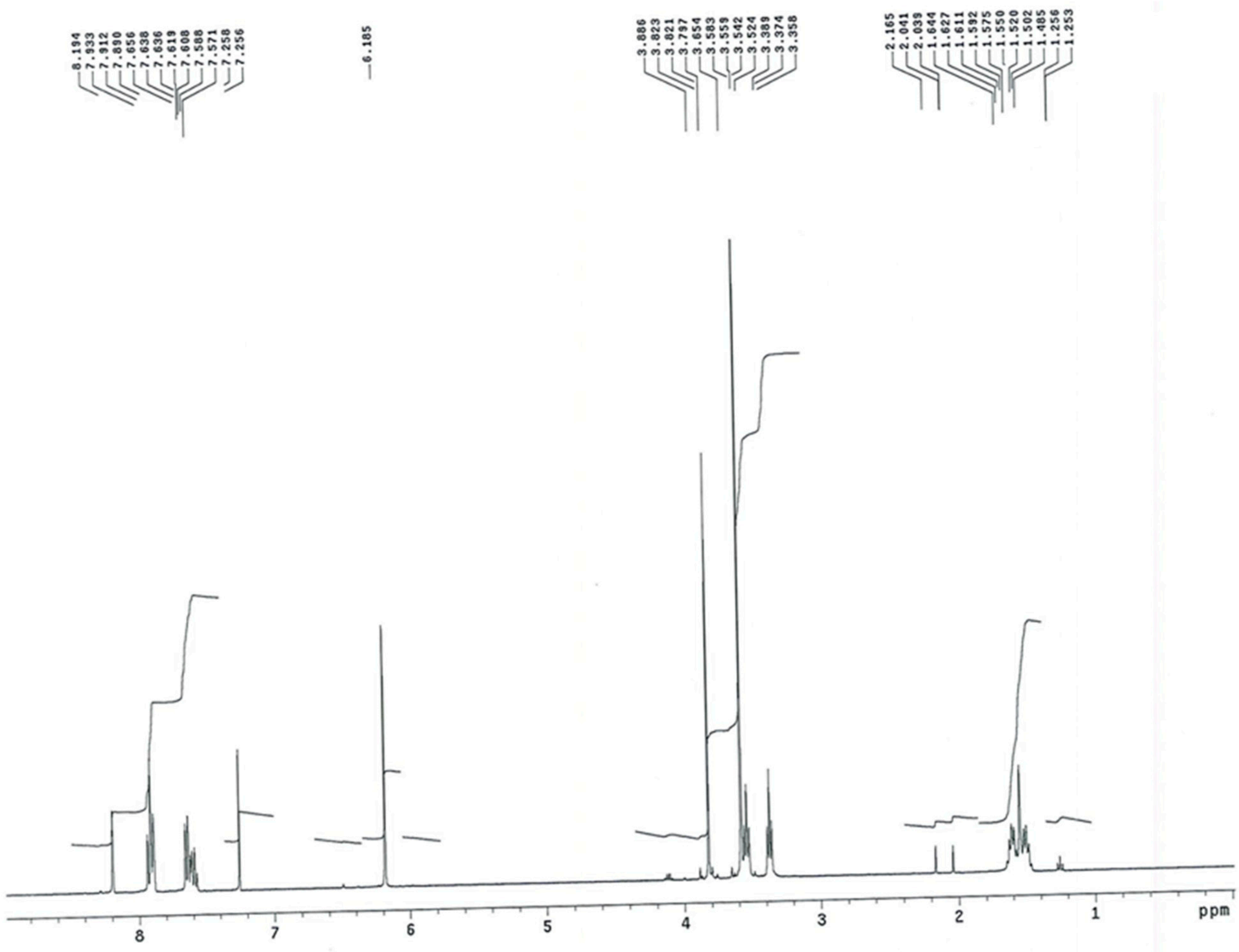

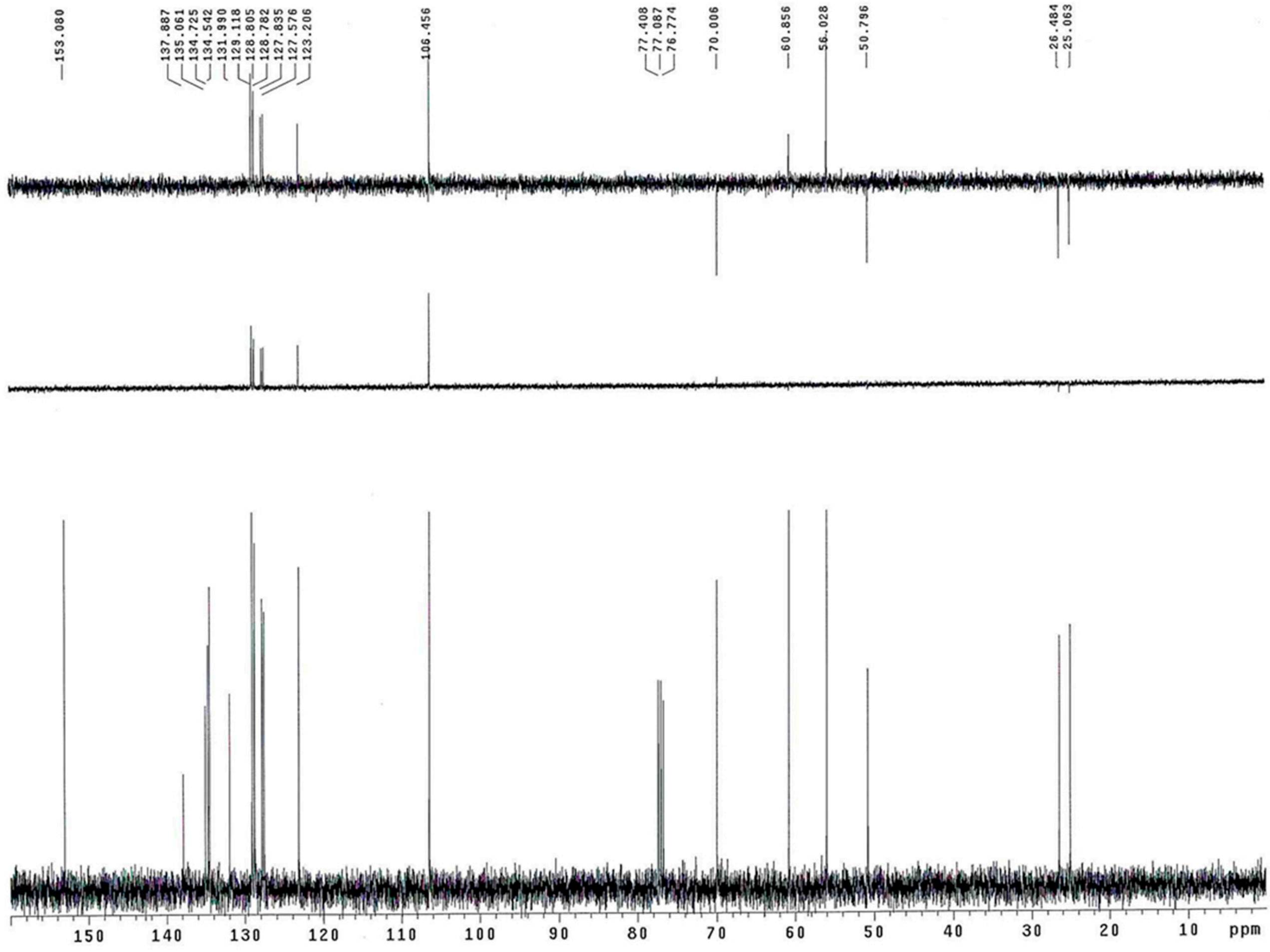

Supp. Fig. S15. ^{1}H and ^{13}C-NMR spectra for compound **2bF**.

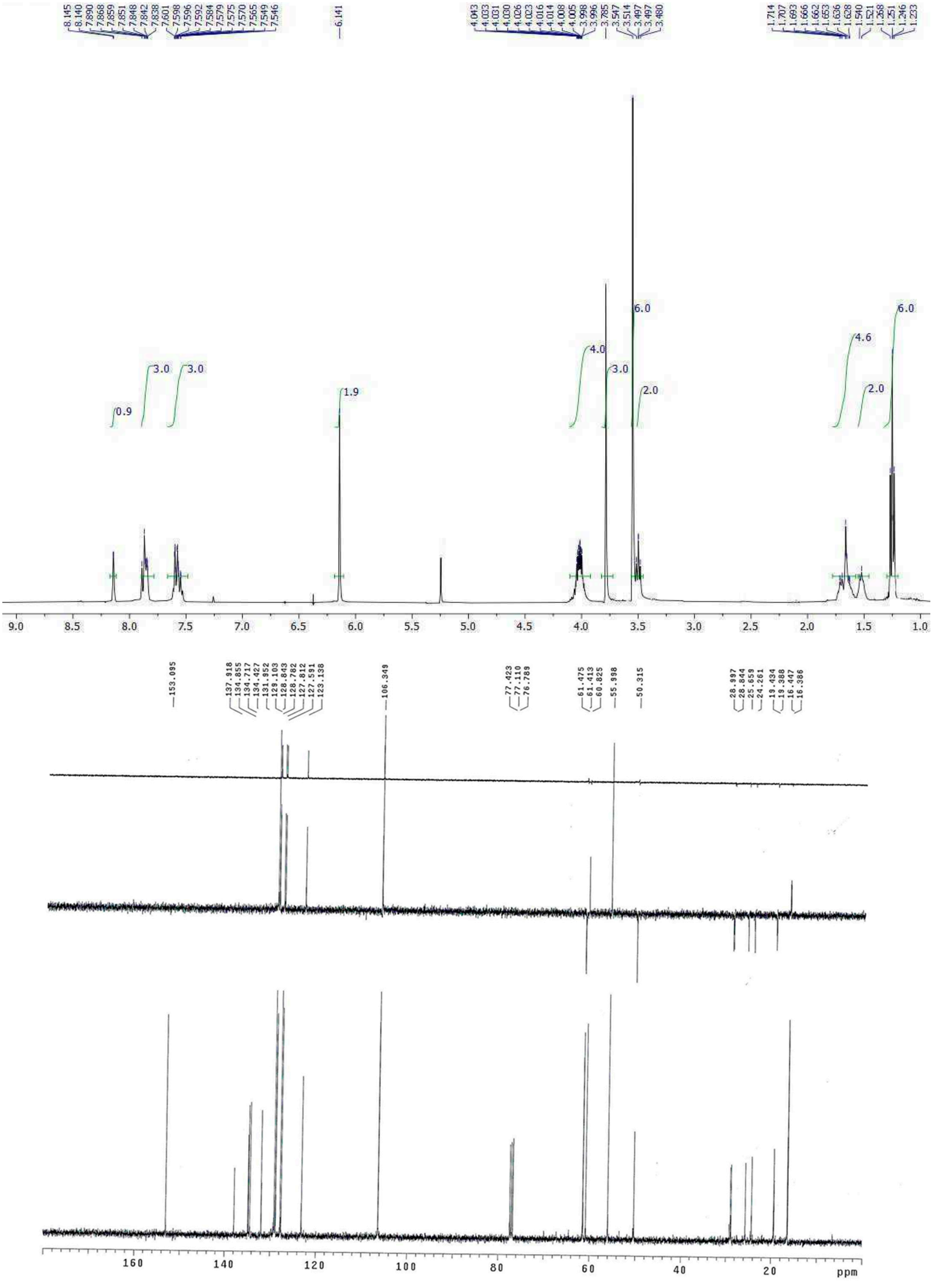

Supp. Fig. S16. ^{1}H and ^{13}C-NMR spectra for compound **3A**.

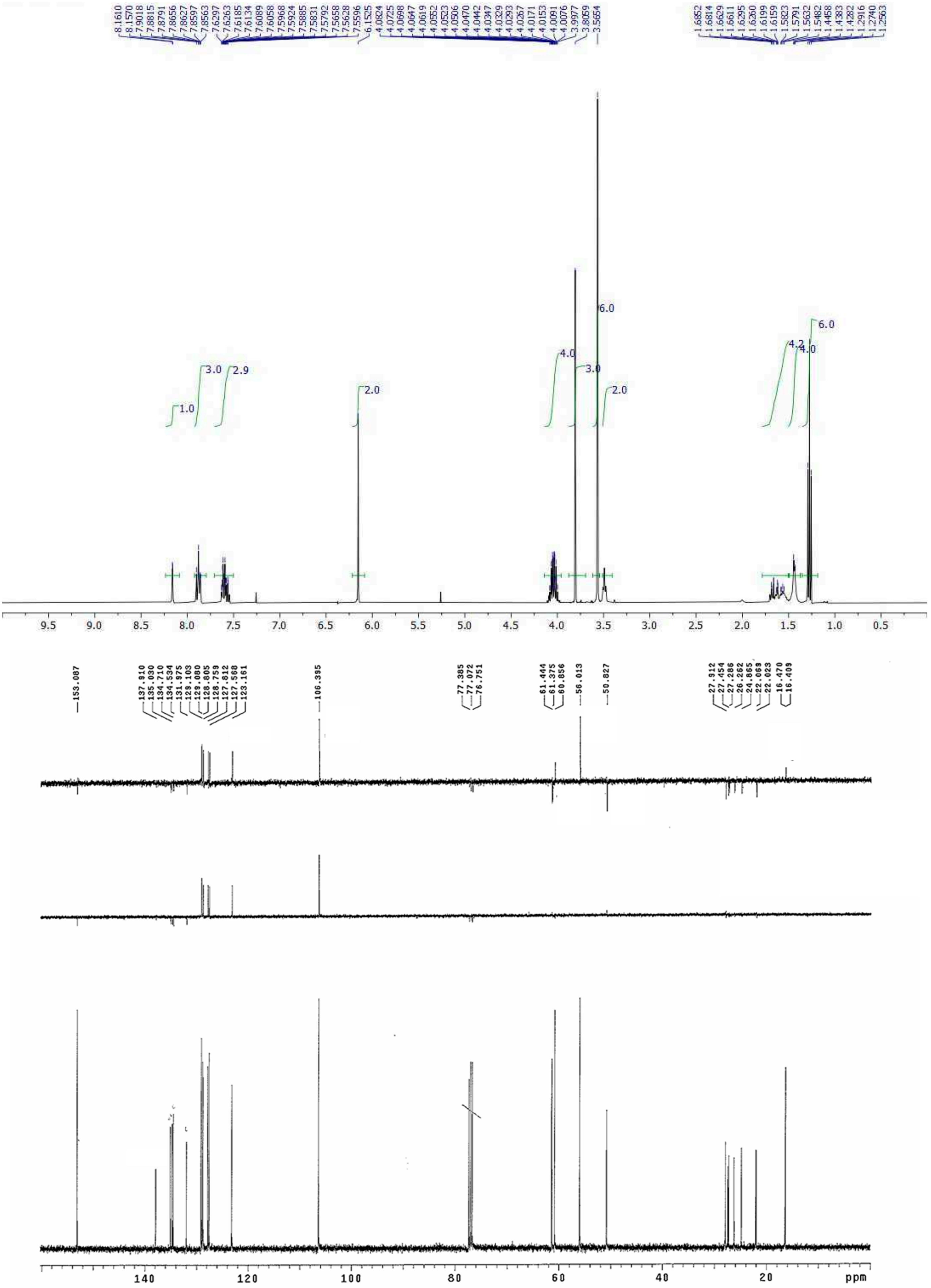

Supp. Fig. S17. ^{1}H and ^{13}C-NMR spectra for compound **3B**.

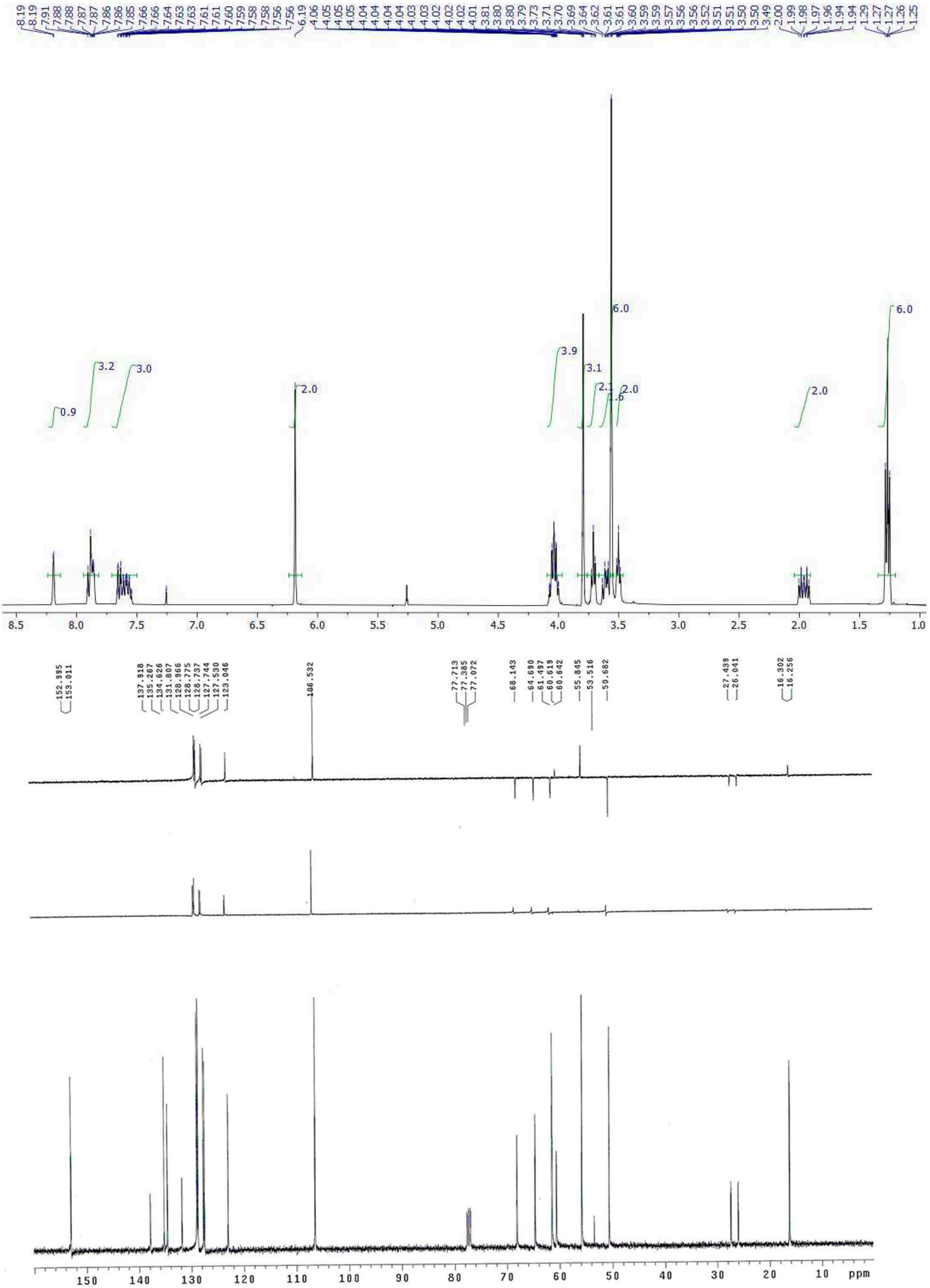

Supp. Fig. S18. ^{1}H and ^{13}C-NMR spectra for compound **3C**.

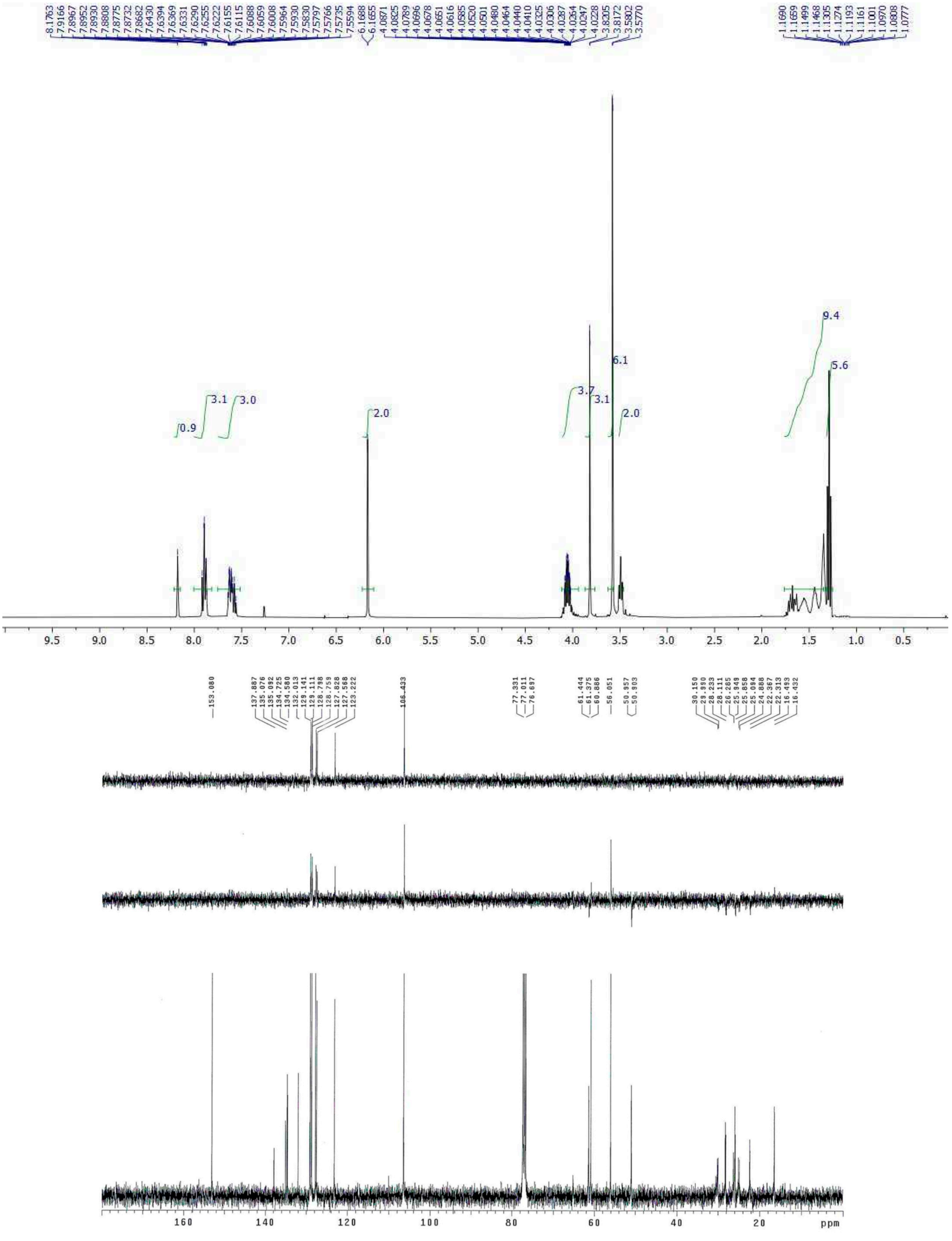

Supp. Fig. S19. ^{1}H and ^{13}C-NMR spectra for compound **3D**.

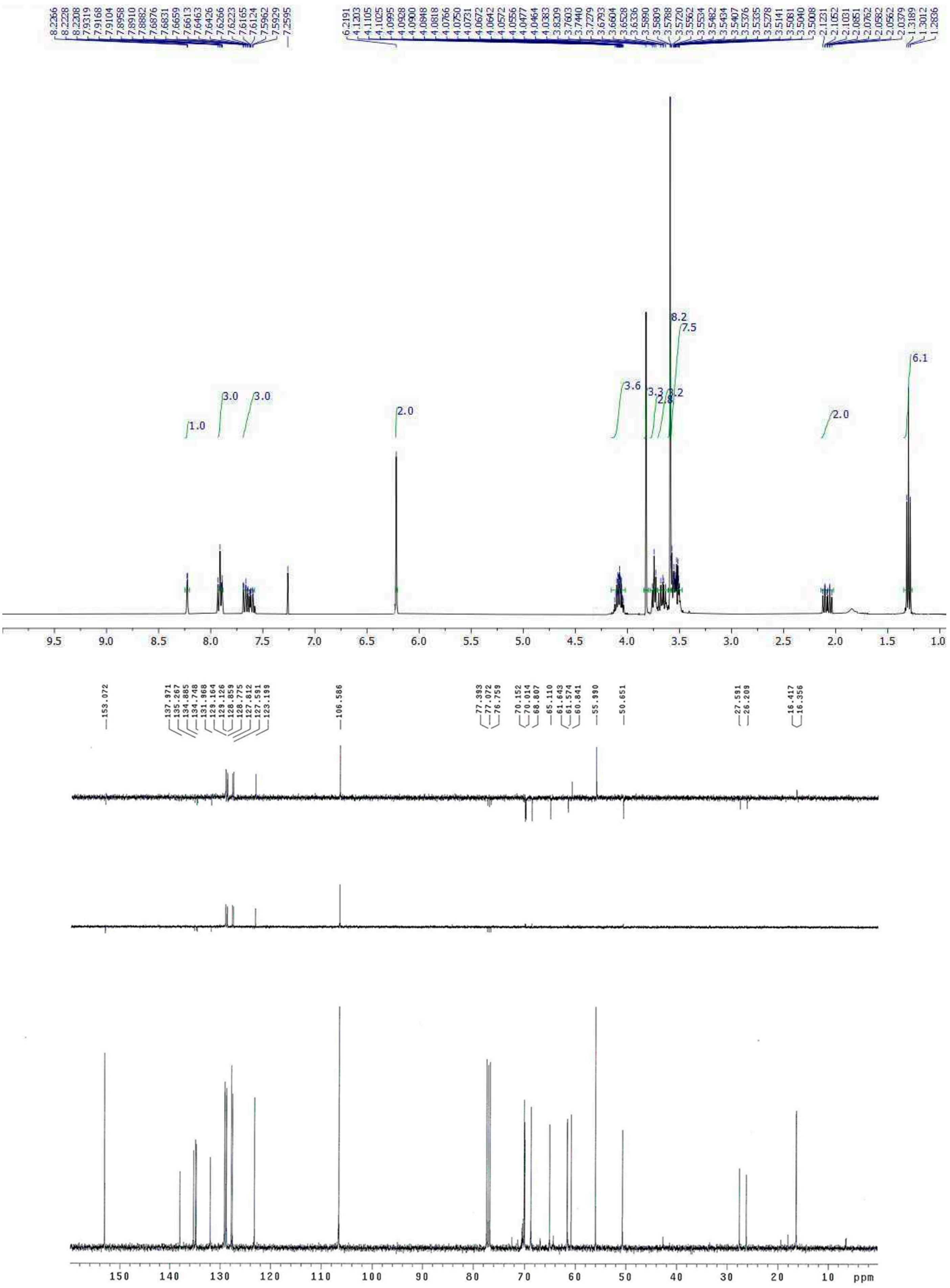

Supp. Fig. S20. ^{1}H and ^{13}C-NMR spectra for compound **3E**.

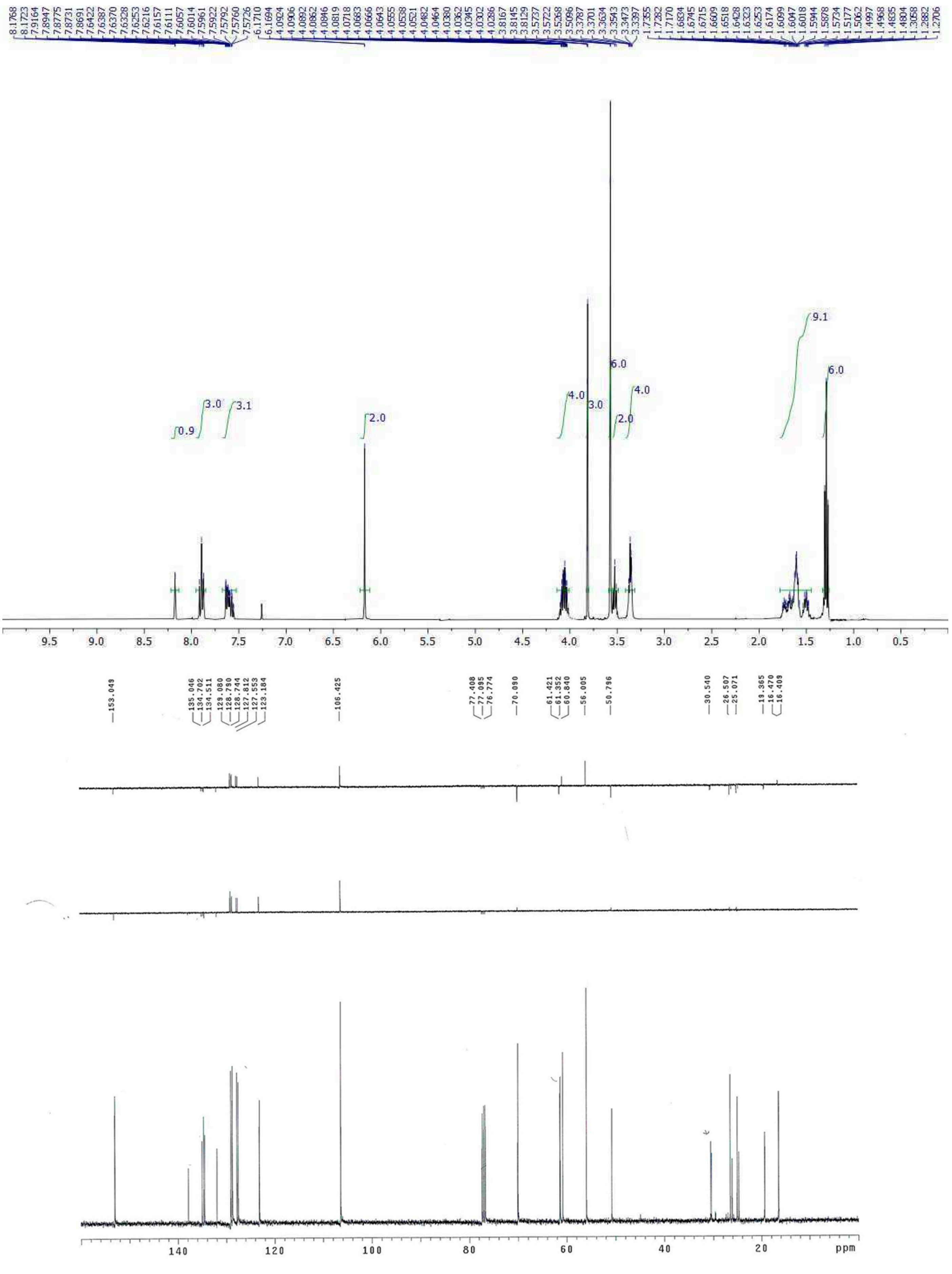

Supp. Fig. S21. ^{1}H and ^{13}C-NMR spectra for compound **3F**.

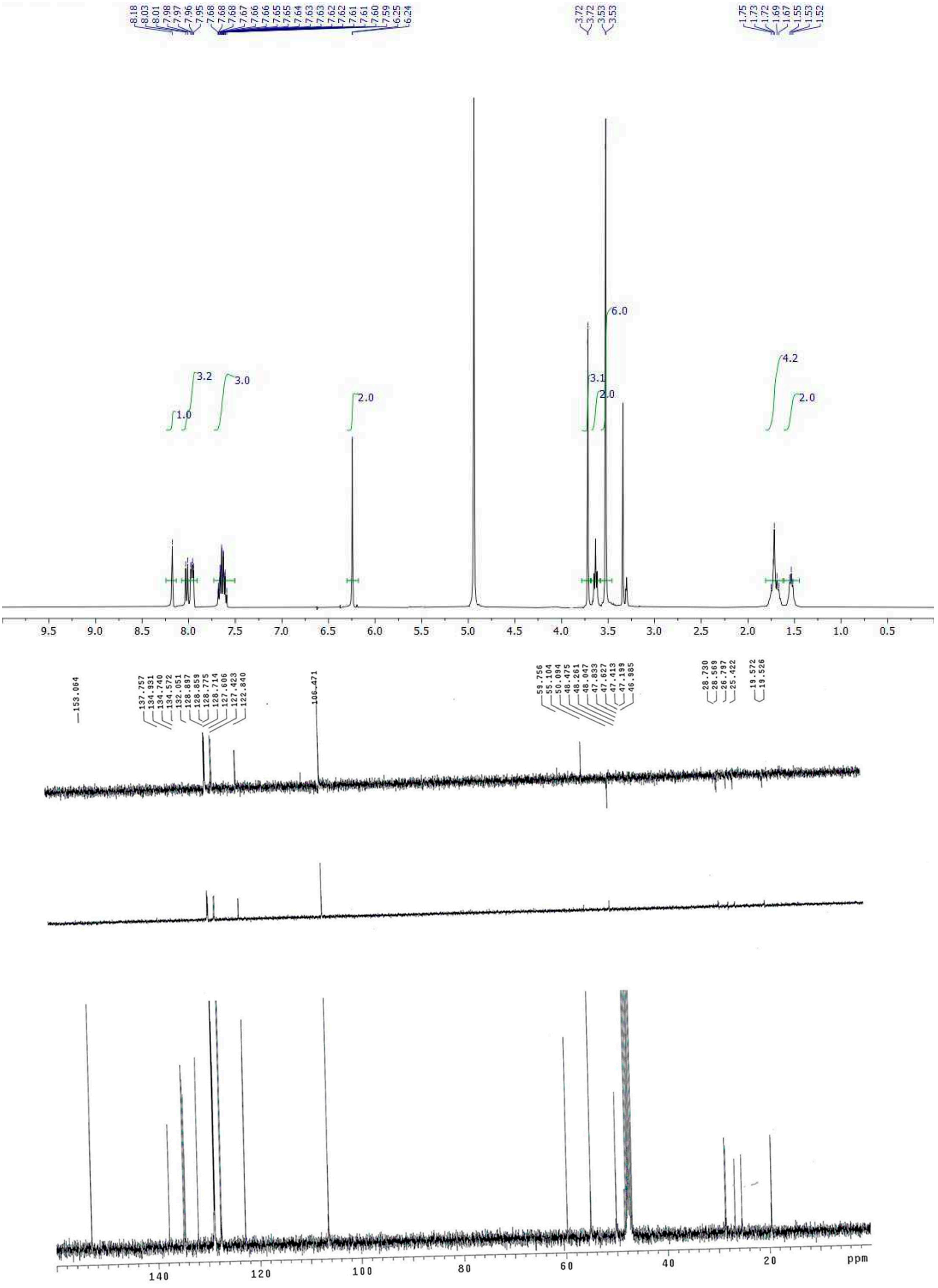

Supp. Fig. S22. ^{1}H and ^{13}C-NMR spectra for compound **4A**.

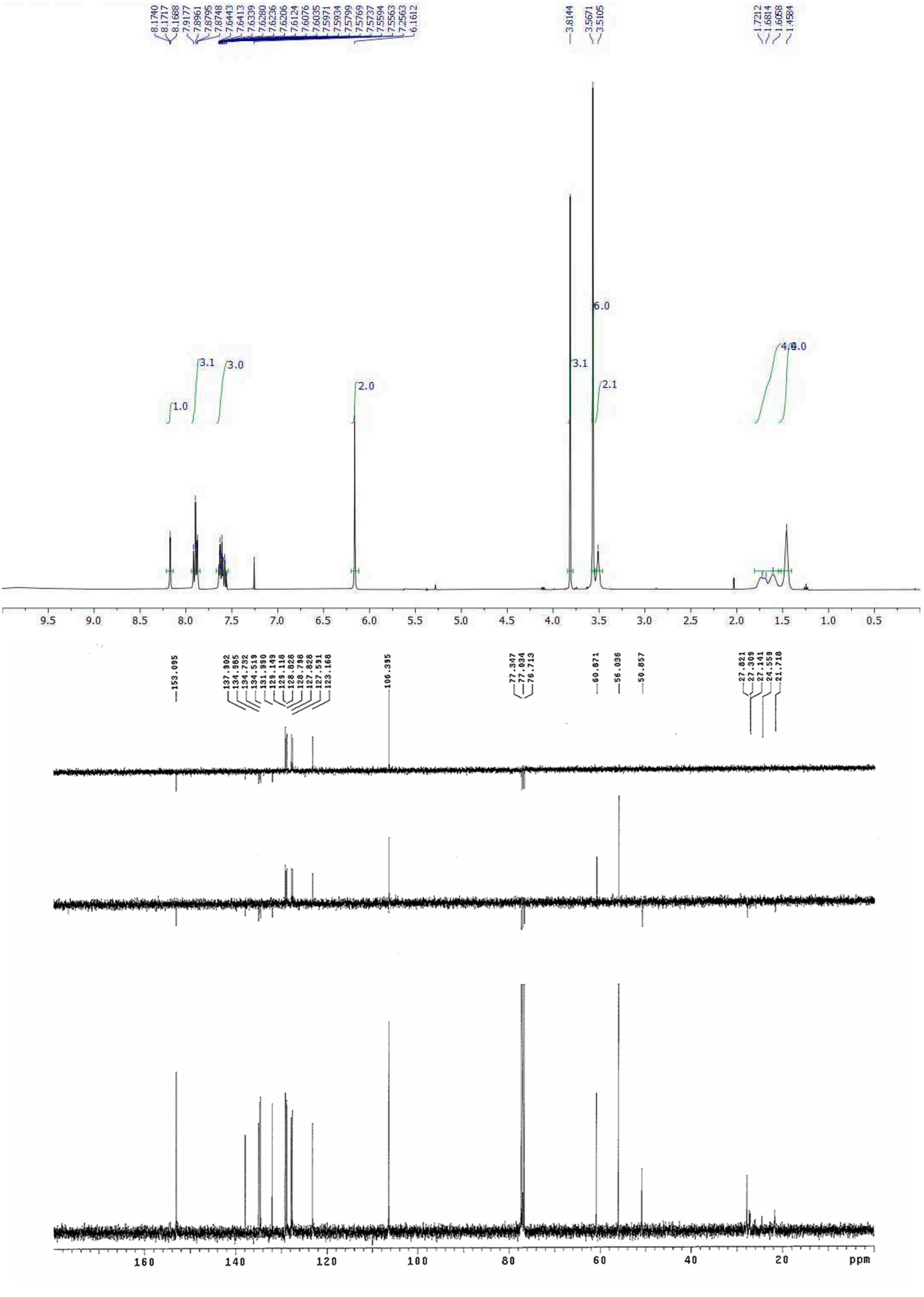

Supp. Fig. S23. ^{1}H and ^{13}C-NMR spectra for compound **4B**.

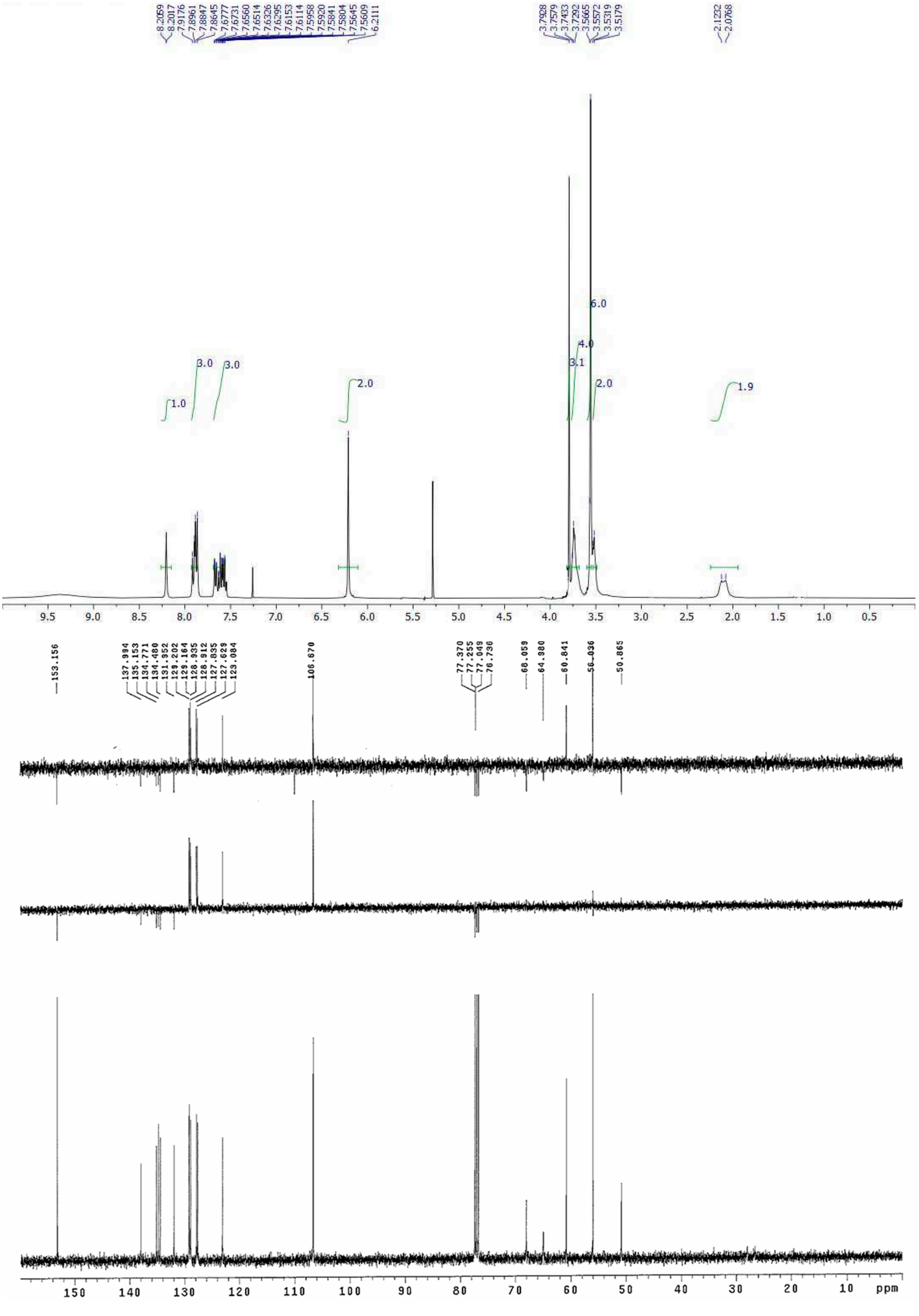

Supp. Fig. S24. ^{1}H and ^{13}C-NMR spectra for compound **4C**.

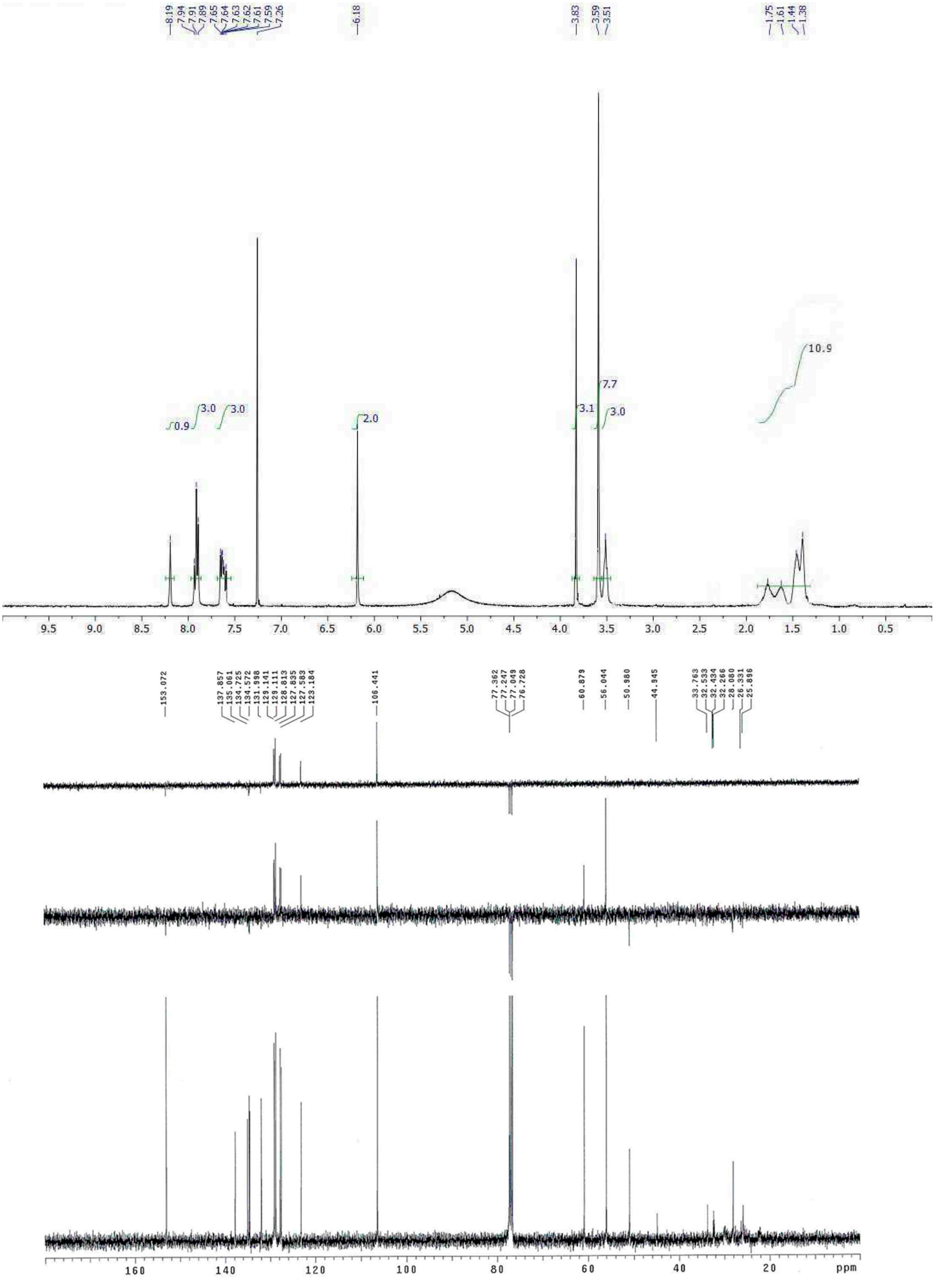

Supp. Fig. S25. ^{1}H and ^{13}C-NMR spectra for compound 4D.

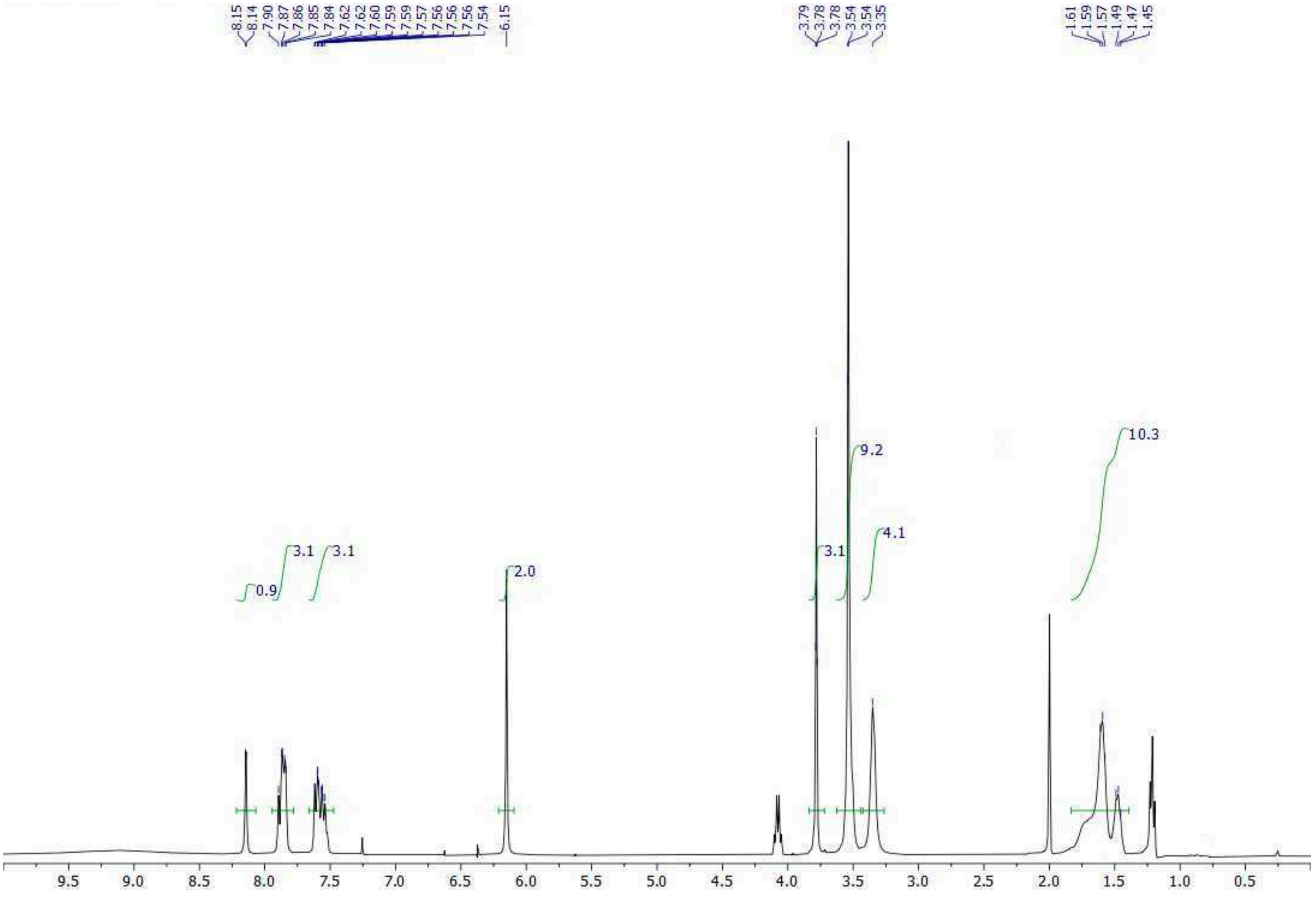

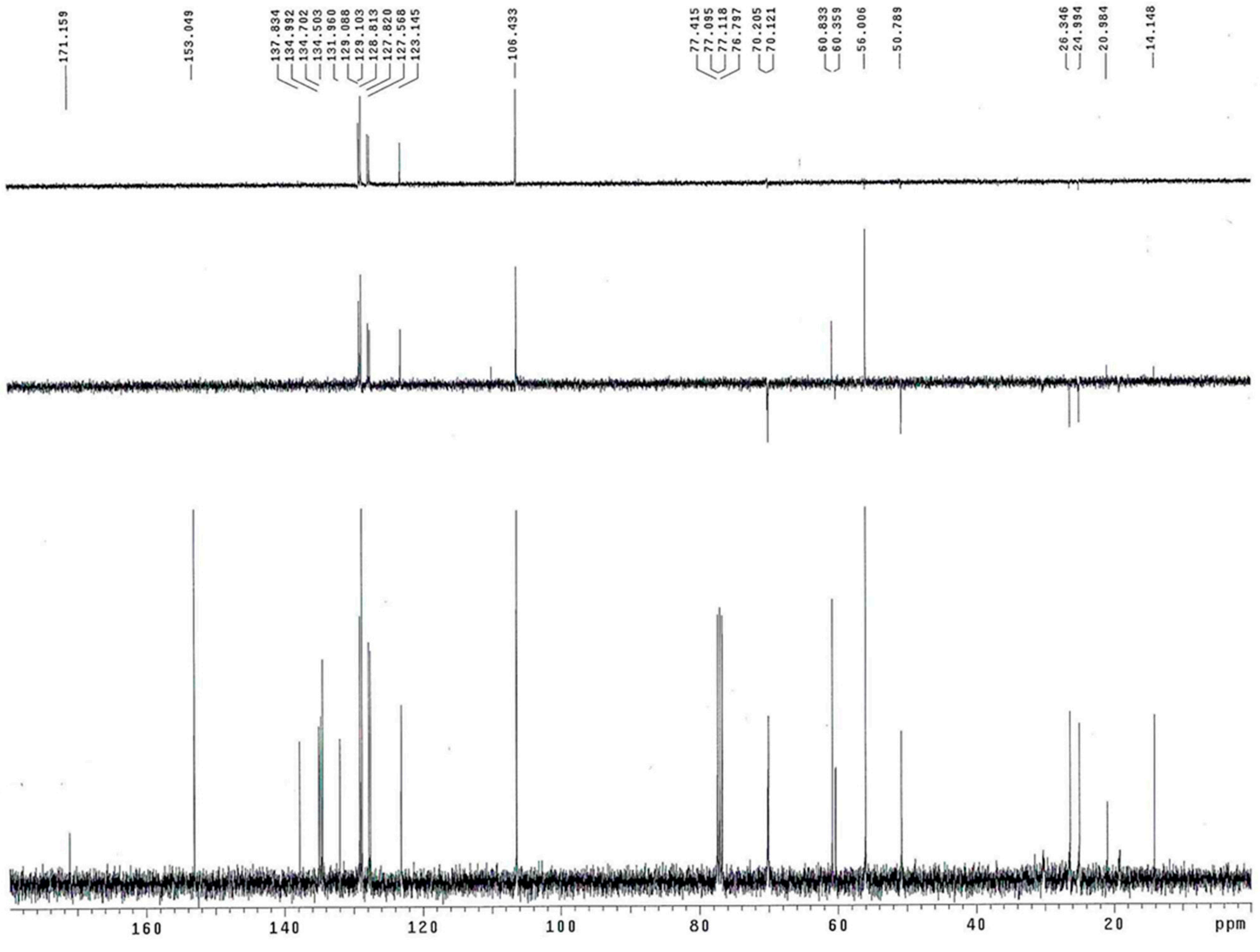

Supp. Fig. S26. ^{1}H and ^{13}C-NMR spectra for compound **4F**.

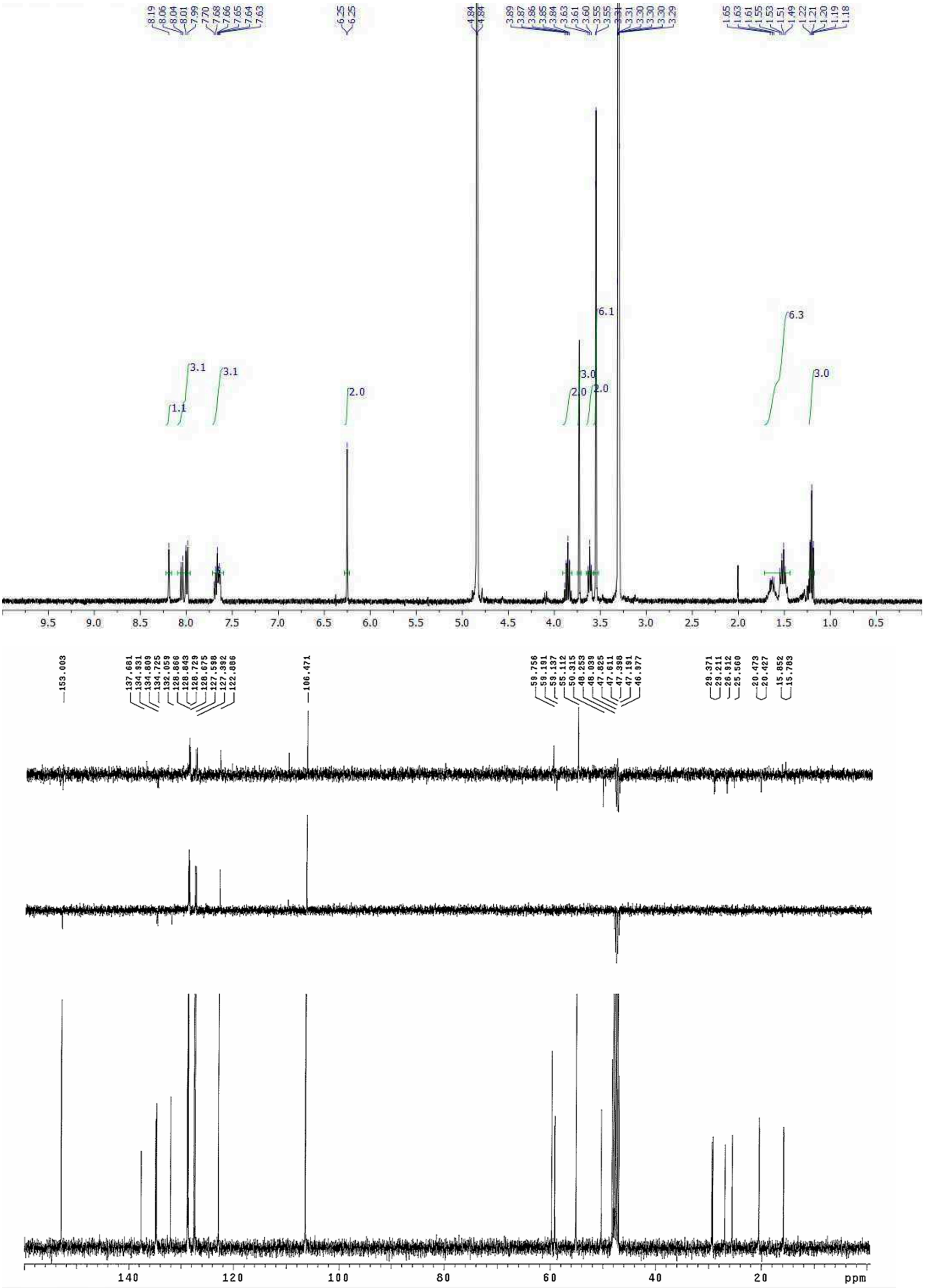

Supp. Fig. S27. ^{1}H and ^{13}C-NMR spectra for compound **5A**.

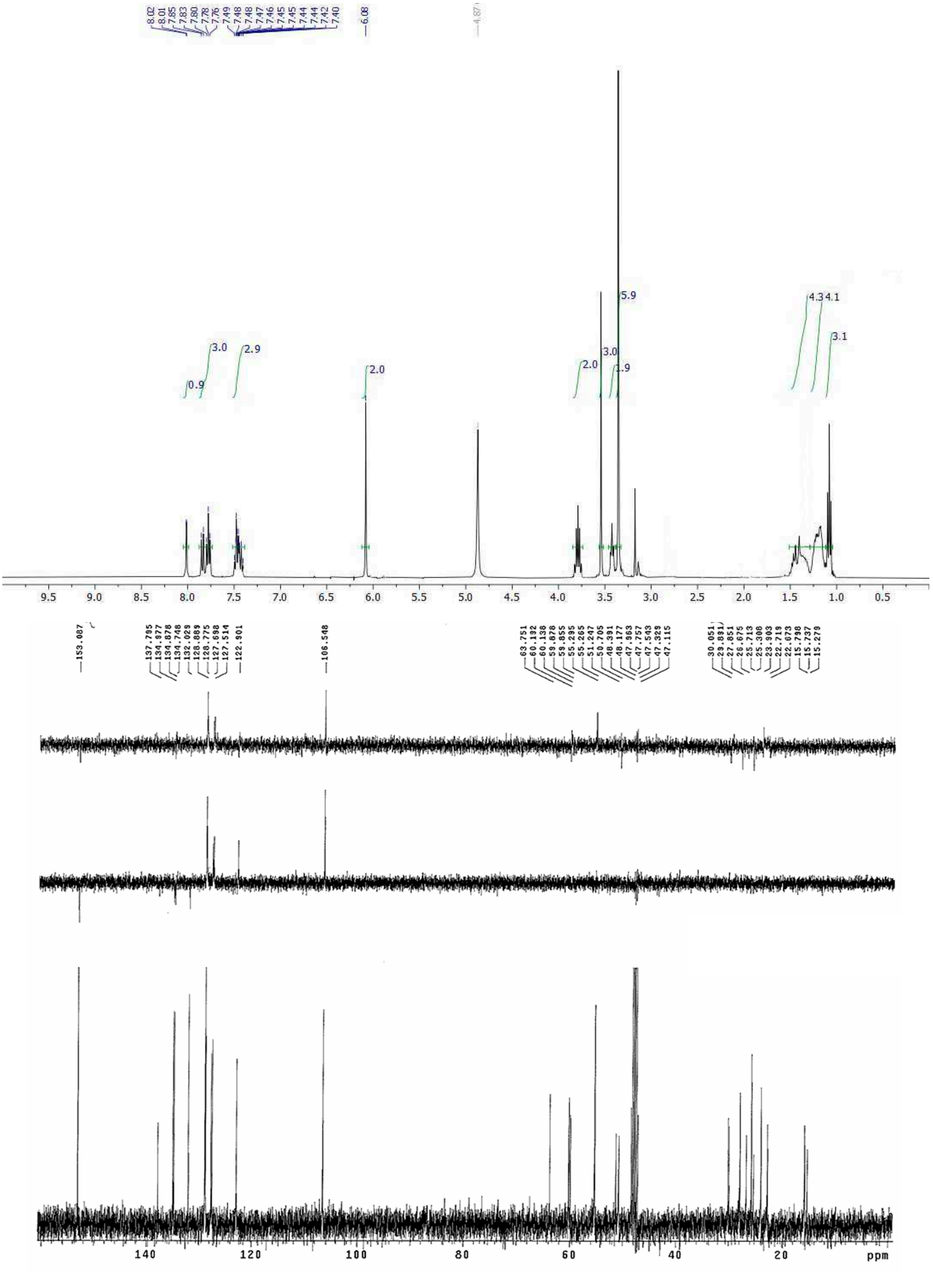

Supp. Fig. S28. ^{1}H and ^{13}C-NMR spectra for compound **5D**.

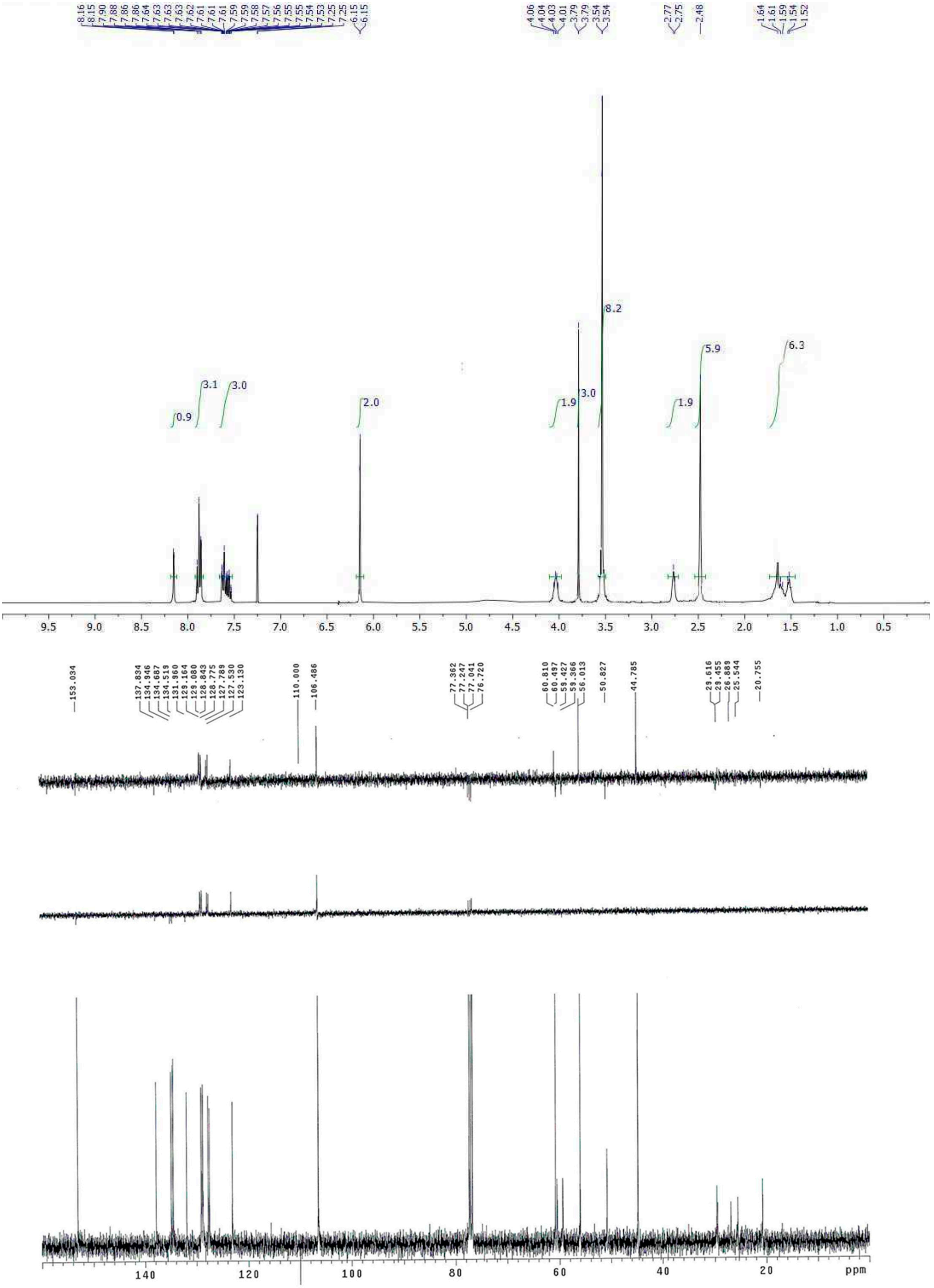

Supp. Fig. S29. ^{1}H and ^{13}C-NMR spectra for compound **6A-DMAE**.

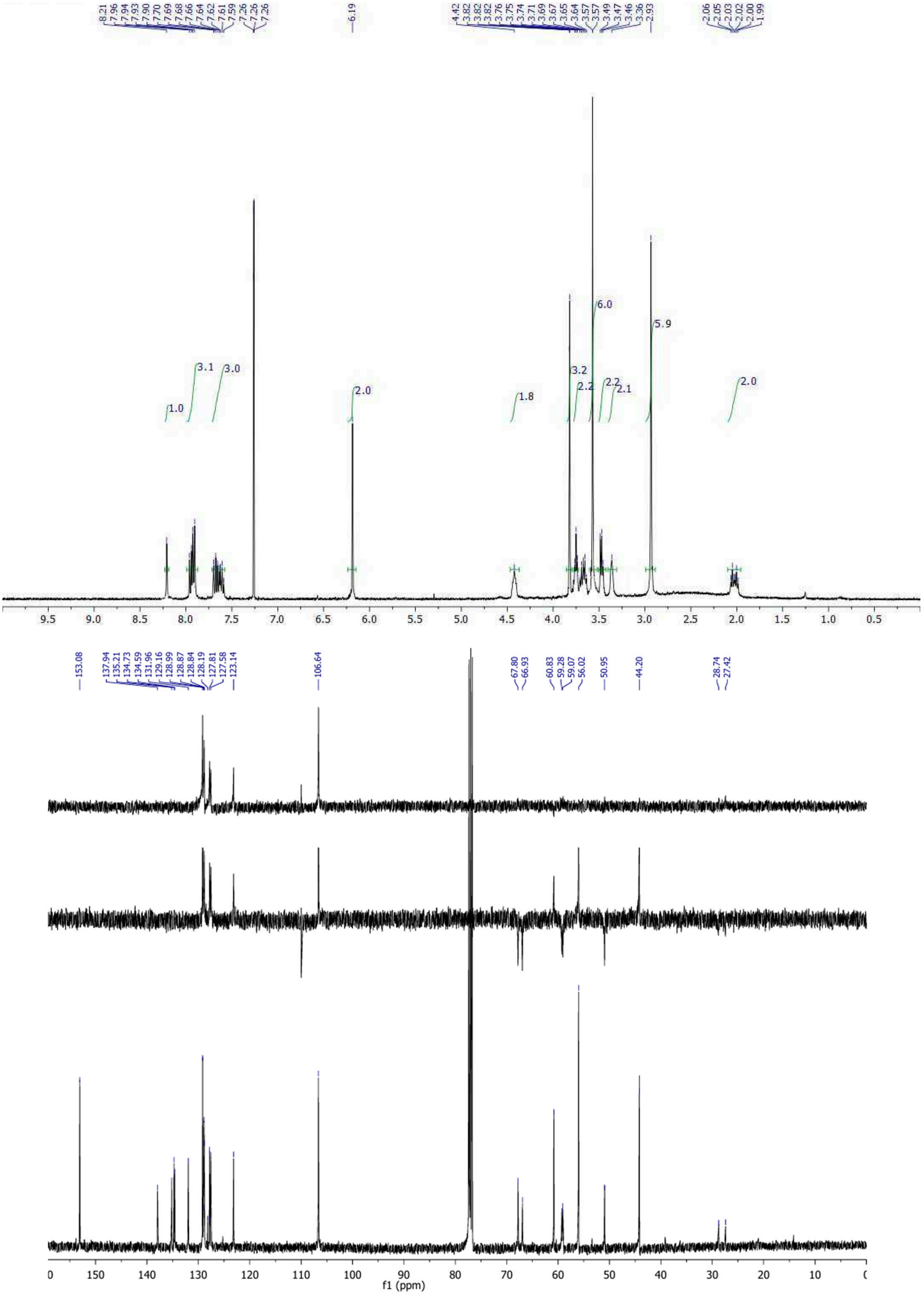

Supp. Fig. S30. ^{1}H and ^{13}C-NMR spectra for compound **6C-DMAE**.

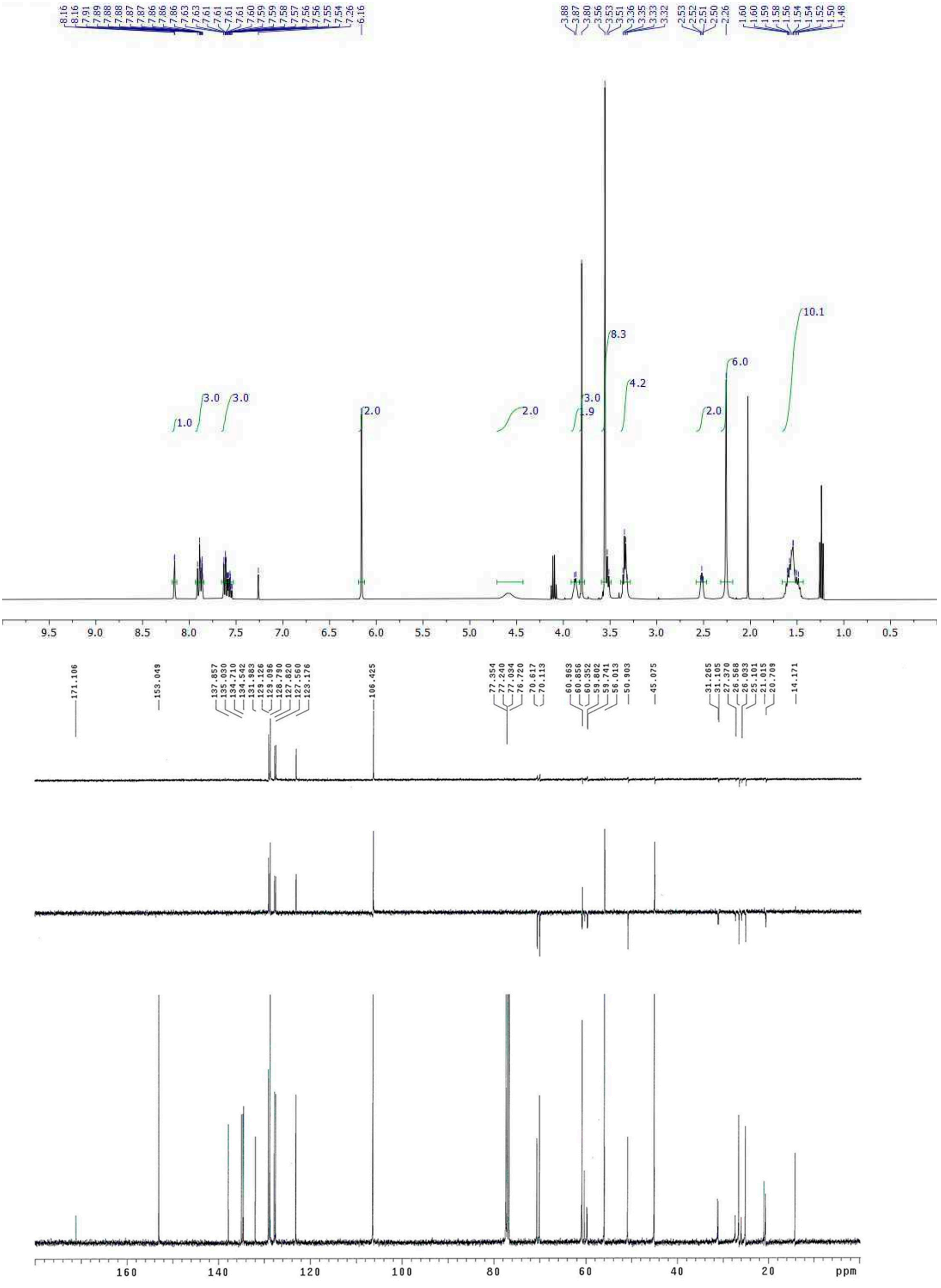

Supp. Fig. S31. ^{1}H and ^{13}C-NMR spectra for compound **6F-DMAE**.

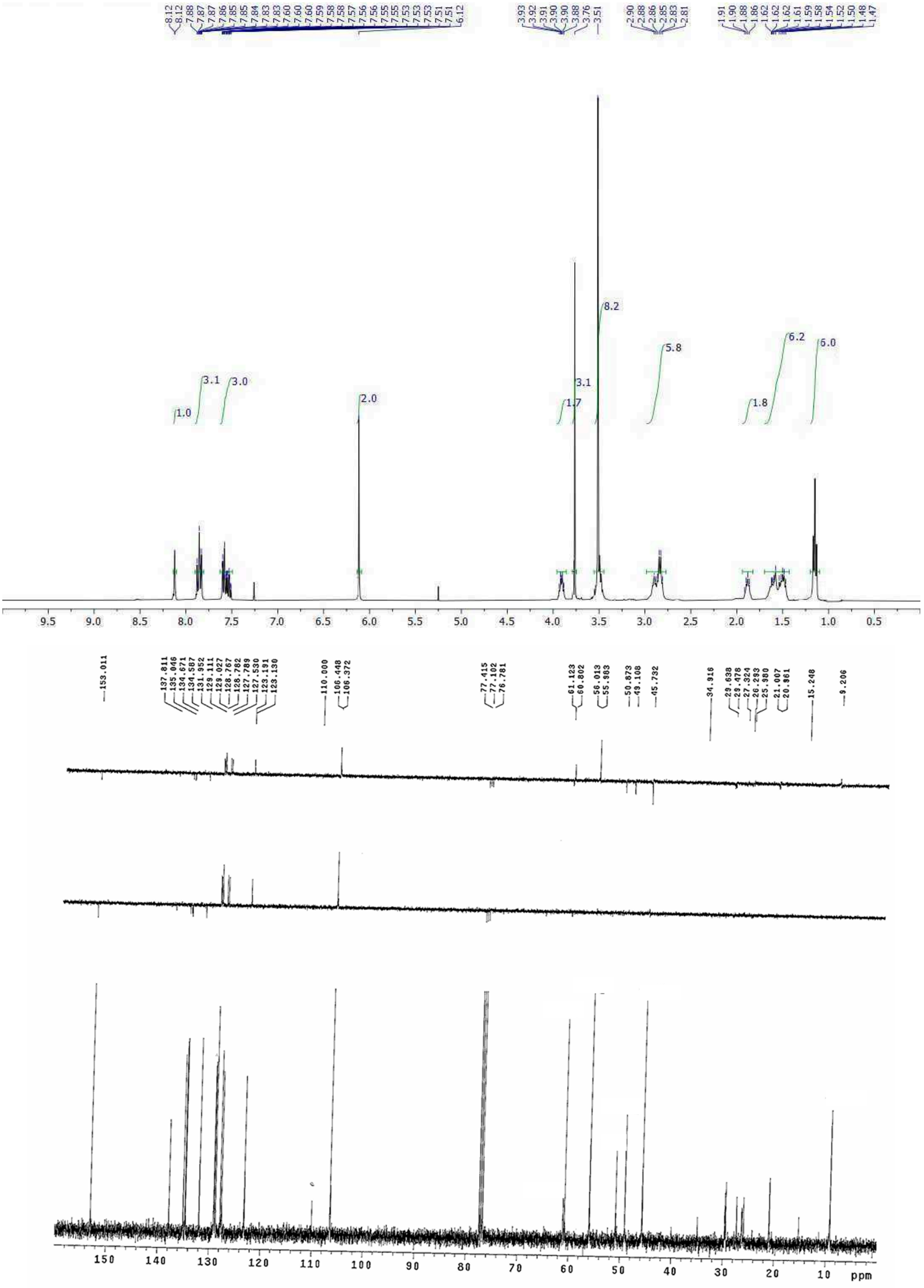

Supp. Fig. S32. ^{1}H and ^{13}C-NMR spectra for compound **6A-DEAP**.

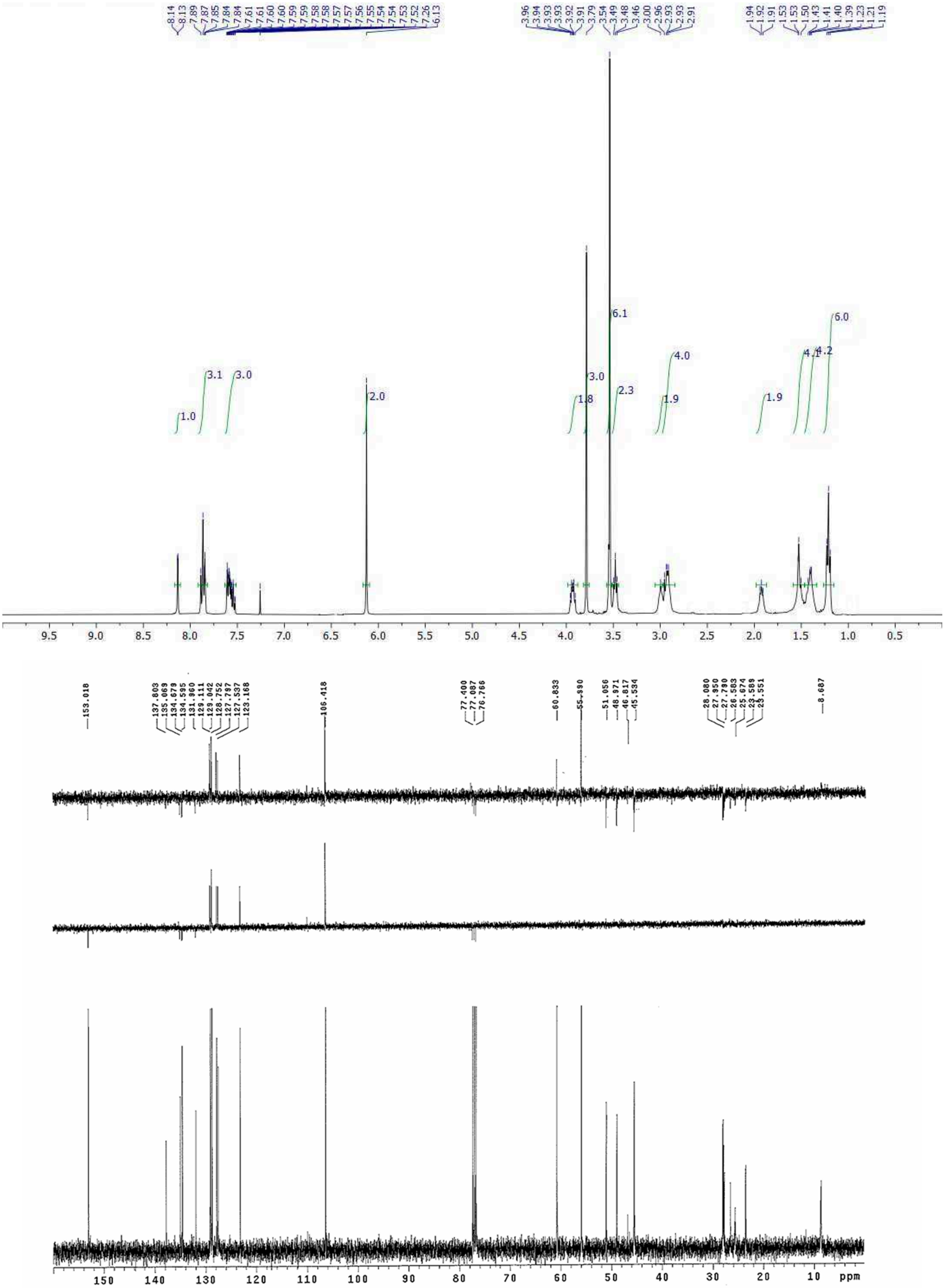

Supp. Fig. S33. ^{1}H and ^{13}C-NMR spectra for compound **6B-DEAP**.

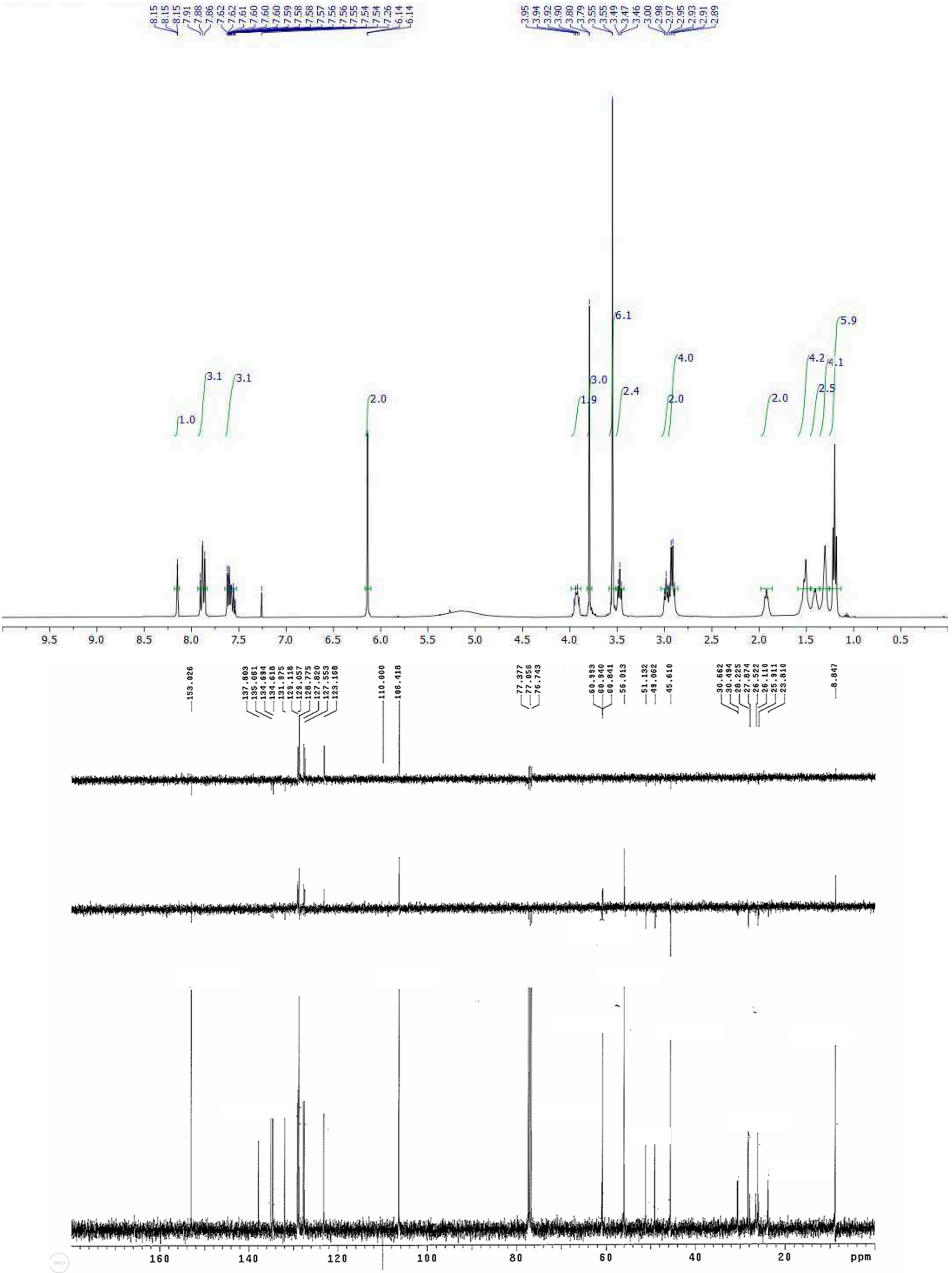

Supp. Fig. S34. ^{1}H and ^{13}C-NMR spectra for compound **6D-DEAP**.

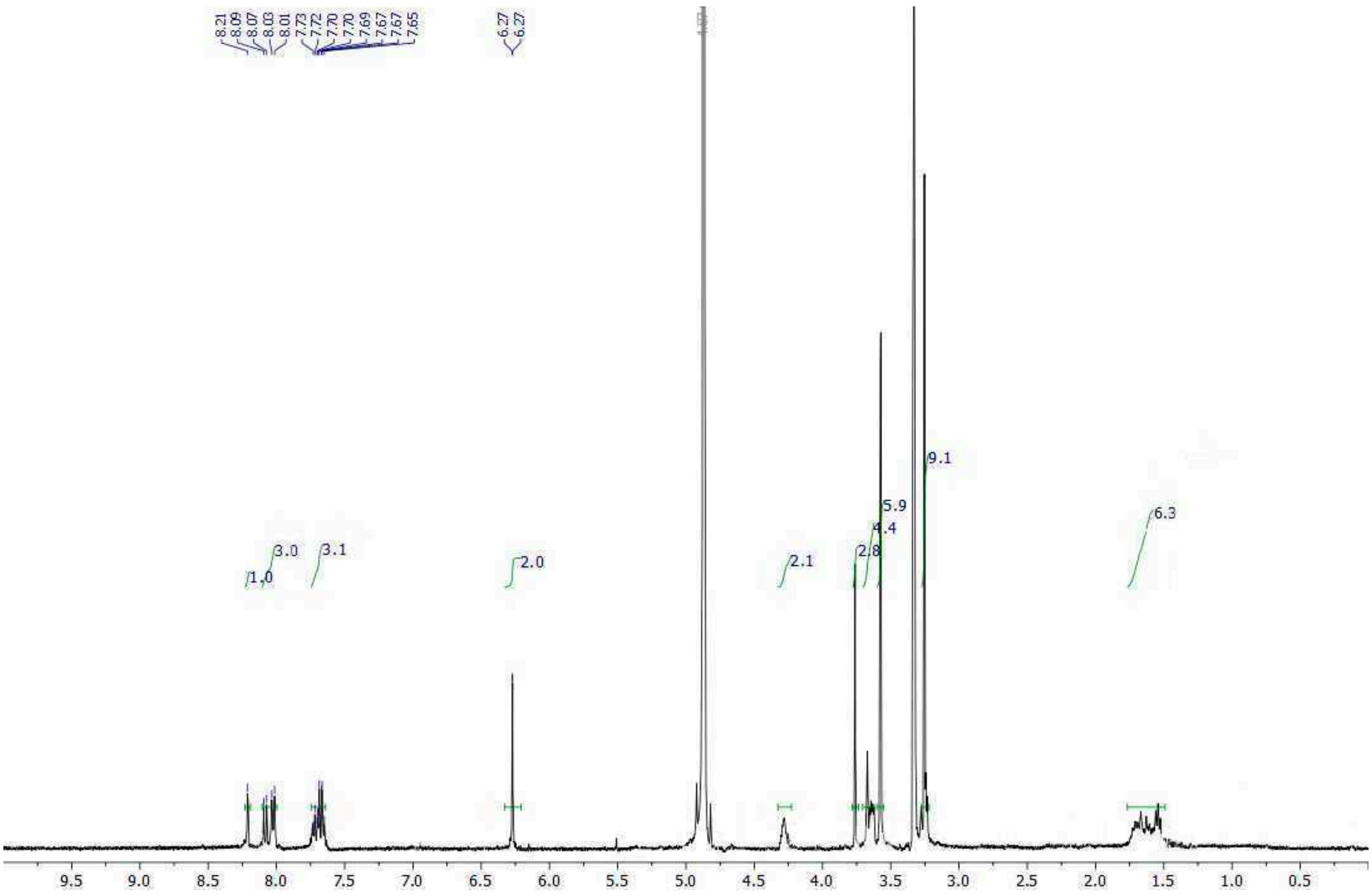

Supp. Fig. S35. ^{1}H NMR spectrum for compound **6A-Ch**.

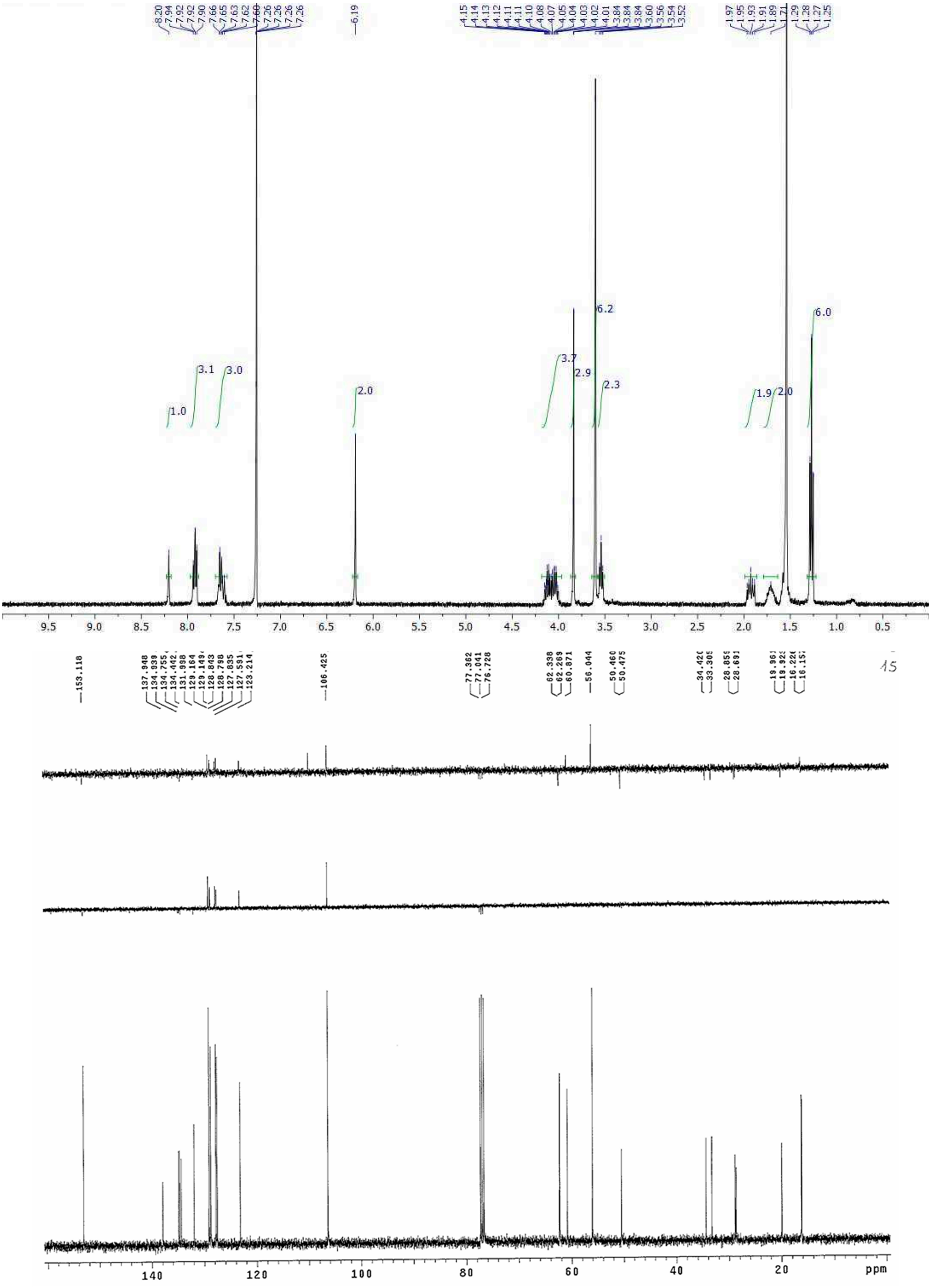

Supp. Fig. S36. ^{1}H and ^{13}C-NMR spectra for compound **7A**.

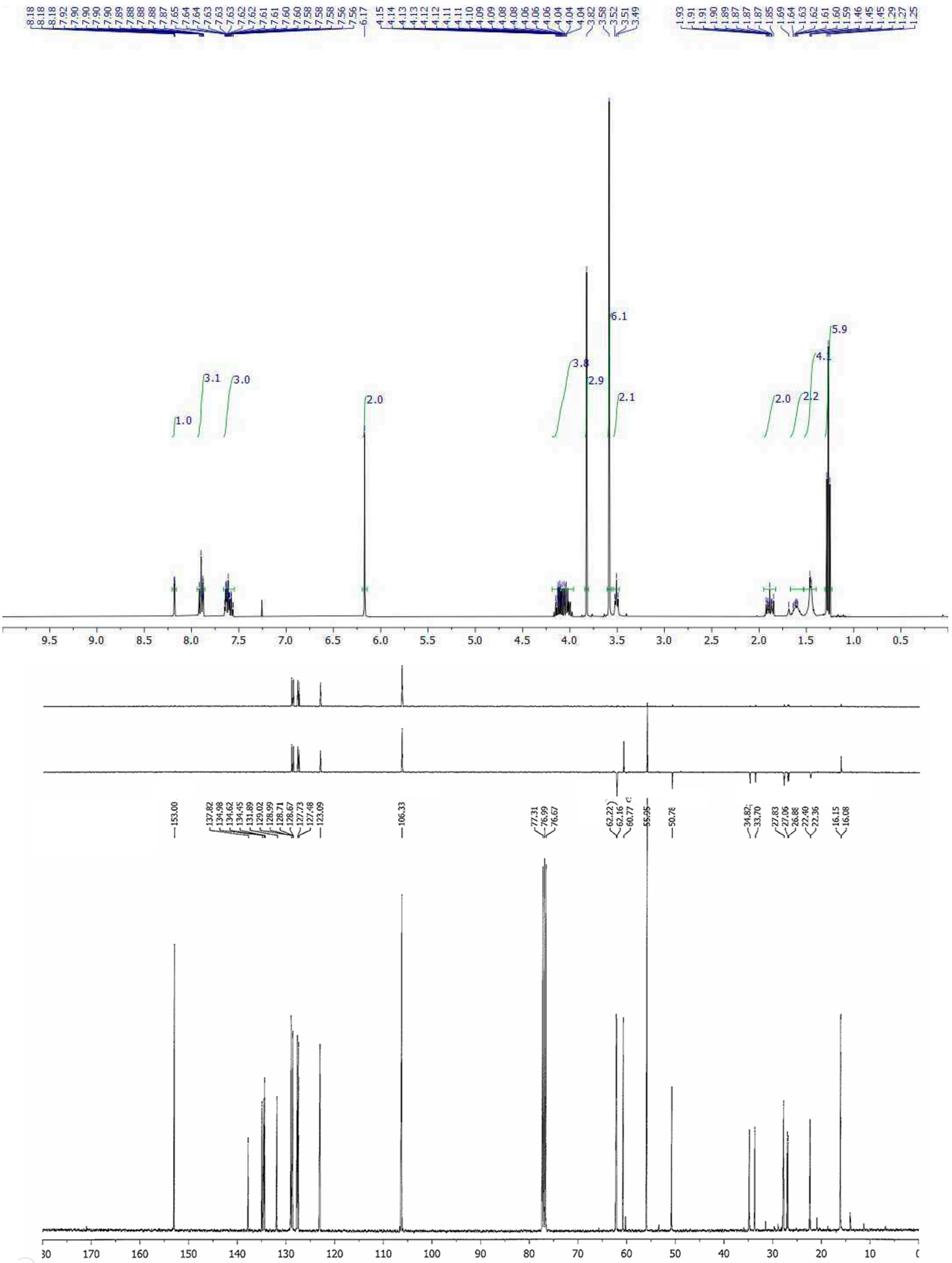

Supp. Fig. S37. ^{1}H and ^{13}C-NMR spectra for compound **7B**.

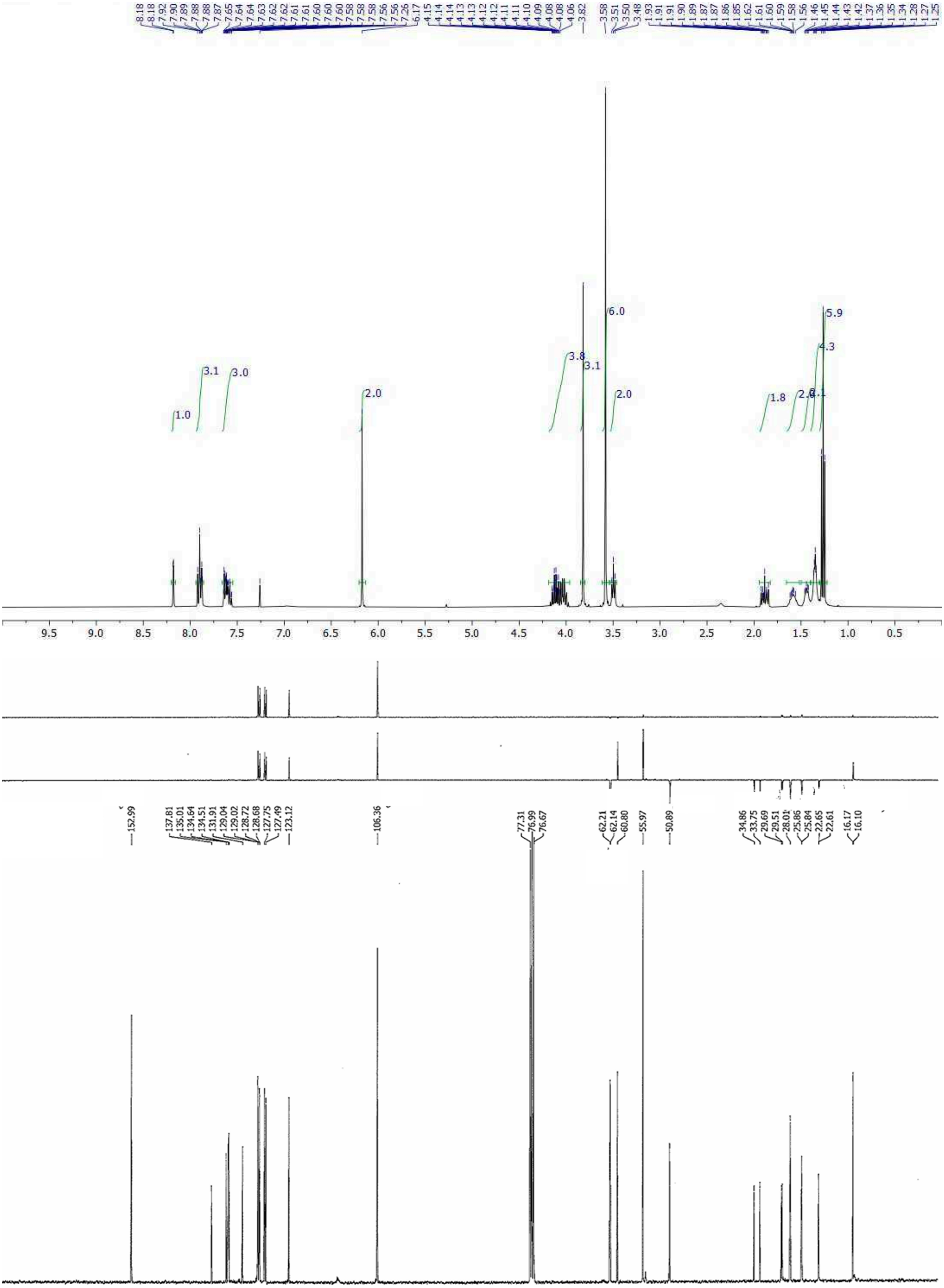

Supp. Fig. S38. ^{1}H and ^{13}C-NMR spectra for compound **7D**.
